# Transcriptional profiling reveals a subset of human breast tumors that retain wt *TP53* but display mutant p53‐associated features

**DOI:** 10.1002/1878-0261.12736

**Published:** 2020-06-23

**Authors:** Gal Benor, Garold Fuks, Suet‐Feung Chin, Oscar M. Rueda, Saptaparna Mukherjee, Sharathchandra Arandkar, Yael Aylon, Carlos Caldas, Eytan Domany, Moshe Oren

**Affiliations:** ^1^ Department of Physics of Complex Systems The Weizmann Institute of Science Rehovot Israel; ^2^ Cancer Research UK Cambridge Institute and Department of Oncology Li Ka Shing Centre University of Cambridge Cambridge UK; ^3^ Department of Molecular Cell Biology The Weizmann Institute of Science Rehovot Israel; ^4^ Advanced Centre for Treatment, Research and Education in Cancer (ACTREC) Tata Memorial Centre Kharghar India

**Keywords:** breast cancer, machine learning, METABRIC, p53 gain of function, pseudomutant p53

## Abstract

*TP53* gene mutations are very common in human cancer. While such mutations abrogate the tumor suppressive activities of the wild‐type (wt) p53 protein, some of them also endow the mutant (mut) protein with oncogenic gain of function (GOF), facilitating cancer progression. Yet, p53 may acquire altered functionality even without being mutated; in particular, experiments with cultured cells revealed that wtp53 can be rewired to adopt mut‐like features in response to growth factors or cancer‐mimicking genetic manipulations. To assess whether such rewiring also occurs in human tumors, we interrogated gene expression profiles and pathway deregulation patterns in the METABRIC breast cancer (BC) dataset as a function of *TP53* gene mutation status. Harnessing the power of machine learning, we optimized a gene expression classifier for ER+Her2‐ patients that distinguishes tumors carrying *TP53* mutations from those retaining wt *TP53*. Interestingly, a small subset of wt *TP53* tumors displayed gene expression and pathway deregulation patterns markedly similar to those of *TP53*‐mutated tumors. Moreover, similar to *TP53*‐mutated tumors, these ‘pseudomutant’ cases displayed a signature for enhanced proliferation and had worse prognosis than typical wtp53 tumors. Notably, these tumors revealed upregulation of genes which, in BC cell lines, were reported to be positively regulated by p53 GOF mutants. Thus, such tumors may benefit from mut p53‐associated activities without having to accrue *TP53* mutations.

## Graphical abstract

In ER+Her2‐ breast cancer (BC), p53 tumor suppressor gene mutations are associated with bad prognosis, relative to tumors that retain wild‐type (wt) p53. Applying machine learning to a large BC dataset reveals a subset of ER+Her2‐ tumors (pseudomutant p53 tumors) which, despite retaining wt p53, display gene expression and pathway deregulation profiles typical of p53‐mutated tumors, correlating with bad prognosis.

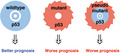

**Abbreviations**
BC: breast cancer
Corr: correlation
FN: false negative
FP: false positive
FSW: forward selection wrapper
GL: gene‐probe lists
GOF: gain of function
GR: gene‐probe ranking
LSVM: linear support vector machine
ML: machine learning
mut: mutant
NGS: next generation sequencing
PDS: pathway deregulation score
PM: pseudomutant
ROC: receiver operating characteristic
SS: Sanger sequencing
wt: wild‐type

## 1 Introduction

One out of eight women is likely to develop breast cancer (BC) during her lifetime (DeSantis *et al*., 2017). BC accounts for 30% of all estimated new cancer cases in women and 25% of all estimated cancer‐related women's deaths. A major challenge in treating BC is its heterogeneity, comprising a large variety of subtypes (Sorlie *et al*., 2001). Understanding each individual patient’s disease better, by its molecular and genomic characterization, is a prerequisite for cancer precision medicine, increasing treatment efficacy while minimizing side effects, and eventually reducing BC mortality (Deng and Nakamura, 2017). With the advent of the big data era, harnessing machine learning (ML) toward the optimization of individualized treatments bears great promise for the future of cancer therapy.

The p53 protein, encoded by the *TP53* tumor suppressor gene, has a central role in safeguarding cells against cancer. Over half of all human cancers carry *TP53* mutations, which may be associated with poor prognosis, increased treatment resistance, and relapse (Silwal‐Pandit *et al*., 2014). The most obvious consequence of such mutations is loss of the tumor suppressive activity of the wtp53 protein. However, some *TP53* mutations may also facilitate cancer progression by endowing the mutant (mut) p53 protein with oncogenic gain of function (GOF) (Brosh and Rotter, 2009). Such GOF, manifested by an increase in proliferation, cell motility, therapy resistance, and more, is driven mainly by interactions of the mut p53 with a variety of other proteins, eventually altering gene expression (Kim *et al*., 2009; Kim and Lozano, 2018). Yet, about half of all human tumors retain wtp53 expression. How do such tumors evade p53's tumor suppressive effects? In some cell types or biological contexts, p53 might lose its tumor suppressive capabilities, alleviating the need to override its effects (Kim *et al*., 2009). Alternatively, p53 may not reach sufficient steady‐state levels because of suppressed transcription of the *TP53* gene (Miller *et al*., 2005), reduced translation, or rapid protein turnover, for example, via augmented MDM2‐mediated degradation (Haupt *et al*., 1997; Kubbutat *et al*., 1997). Additionally, wtp53 may undergo aberrant post‐translational modifications or be excluded from the cell nucleus, depriving it of its ability to act as a transcription factor. All these mechanisms may result in p53 loss of function, equivalent to genetic loss of both wt *TP53* alleles.

Nevertheless, wtp53 may sometimes not only lose its normal activity, but also acquire structural and functional properties resembling those of *bona fide* GOF mut p53 proteins (Furth *et al*., 2015, 2018; Milner, 1995; Trinidad *et al*., 2013; Zhang and Deisseroth, 1994). Such ‘pseudomutant’ (PM) wtp53 may become an active contributor to cancer (Furth *et al*., 2018). An early study by Miller *et al*. (2005), looking at data from 251 breast tumors, identified a group of wtp53 cases that were labeled as mut on the basis of their gene expression patterns.

Of note, the study of Miller *et al* was performed on a mix of tumors of all BC subtypes, analyzed together as one population. However, the relative abundance of *TP53* mutations varies greatly between subtypes; for example, ER+tumors are predominantly wt *TP53*, whereas a large proportion of ER‐ tumors carry *TP53* mutations. Thus, part of the reported differences in transcription profiles between wt and mut p53 were most probably due to their unequal representation in the different BC subtypes. Indeed, the wtp53 transcriptional signature defined by Miller et al was enriched in estrogen‐inducible genes (Miller *et al*., 2005), which might simply reflect the fact that the great majority of wtp53 tumors are ER+.

To avoid a bias introduced by the unequal frequency of *TP53* mutations in the different BC subtypes, we chose to compare the expression patterns of wt and mut *TP53* tumors only within the same subtype. We believe that this approach eliminates the ‘noise’ occurring from comparisons between different subclasses of disease and thus enables rigorous identification of wt *TP53* tumors that indeed behave in a mut‐like manner.

We interrogated each BC subtype separately for the existence of ‘PM’ tumors that harbor wt *TP53* by sequence, but nevertheless exhibit features suggestive of the presence of a mut‐like p53 protein. To that end, we used the METABRIC BC dataset, in which both the transcriptome (Curtis *et al*., 2012; Rueda, 2019) and the p53 mutation status (Pereira *et al*., 2016; Silwal‐Pandit *et al*., 2014) are described for a large number of samples. Using ML, we constructed a classifier that best distinguished wtp53 tumors from those carrying *bone fide TP53* mutations. Subsequently, we searched for tumors that were identified by our classifier as mut, despite harboring wt *TP53*. We found among ER+Her2‐ BCs a subgroup of PM p53 tumors, with a transcriptome resembling that of true mut p53 tumors. Moreover, like ER+Her2‐ BC with authentic p53 mutations, these PM tumors exhibited an enhanced proliferation signature and were associated with worse prognosis. Understanding the mechanisms that underlie the aberrant behavior of p53 in such tumors may provide clues toward individualized treatment, with improved patient outcome.

## 2 Materials and methods

### 2.1 Aims and strategy

#### 2.1.1 Aims

To identify PM tumor samples and to characterize these tumors and patients.

#### 2.1.2 Strategy

Using ML on single probe expression data, we constructed a robust conservative classifier that assigns each sample a (learned) label of either wt or mut p53. False‐positive (FP) samples, which are labeled as mut even though they are wt, are designated as PM.

### 2.2 Data

Probe‐level expression data from the METABRIC dataset (Curtis *et al*., 2012) were filtered and preprocessed for 1928 tumor samples for which we had both expression and *TP53* status (see Data S1). The final expression table, of 34,363 (HT‐12 v3 platform, Illumina_Human_WG‐v3) probes representing 24,369 genes, for 1928 tumor and 144 normal samples, can be found in Appendix S1 (provided upon request). ‘Ground truth’ *TP53* mutation status was from sequencing: from next generation sequencing (NGS) when available (Pereira *et al*., 2016), or from Sanger Sequencing (SS) (Silwal‐Pandit *et al*., 2014). For the learning process, samples without *TP53* mutations and with synonymous mutations were labeled wt, and everything else was labeled mut. In some analyses, we separated missense from all other mutations (the latter are referred to as ‘null’). Samples that were suspected to harbor germline mutations (*n* = 15) were excluded from the analysis (Table S1). Our *TP53* labels, the mutation types and BC subtypes (ER+Her2‐, ER‐Her2‐, and Her2+, as determined by immunohistochemistry), are summarized in Table 1 and given in detail in Appendix S2, which contains also protein change, survival, and treatment information and clinical parameters. Learning, classification, validation, and subsequent analyses were done separately for each clinical subtype.

**Table 1:** Number of samples of each clinical BC subtype and p53 mutation status. METABRIC data used for wt/mutp53 classification: see text for definitions of labels.

Subtype\p53 status	p53 wt	p53 missense	p53 null	Total
ER+Her2‐	1122	153	98	1373
ER‐Her2‐	63	144	108	315
Her2+	78	95	67	240
Total	1263	392	273	1928

### 2.3 Focus on the ER+Her2‐ subtype

Since each BC subtype is a distinct disease with different molecular characteristics, we analyzed tumors from these three subtypes separately and independently. The relative abundance of *TP53* mutations varies greatly between these subtypes, which biases the analysis of the transcriptional effects of such mutations when all subtypes are combined together into a single group (Miller *et al*., 2005). We therefore chose to focus on ER+Her2‐ tumors: This group comprises by far the largest number of samples of the same subtype, ensuring best statistics. ER+Her2‐ tumors exhibit pronounced misbalance toward wt *TP53* (1122 wt and 251 mut samples), whereas the Her2+ and ER‐Her2‐ groups exhibit the opposite misbalance; this also motivated us to focus on the ER+Her2‐ subtype. Analyses that try to compensate the learning algorithm for imbalanced training sets introduce several uncontrolled parameters. To avoid this, we used learning processes that do not correct for imbalance. In the ER+Her2‐ group, lack of compensation for the prevalence of wt samples gives rise to a conservative classifier, with a bias toward a wt call. Hence, we have high confidence in the classification of those wt *TP53* samples whose transcriptome deviated so strongly from wt behavior, that a mut call was generated; confidence in identification of such ‘false positives’(FP) is essential for fulfilling the aim of this analysis. With this said, we also ran learning processes that did compensate for sample misbalance (see Data S2).

### 2.4 Constructing the wt/mutp53 classifier

**Fig. 1:**
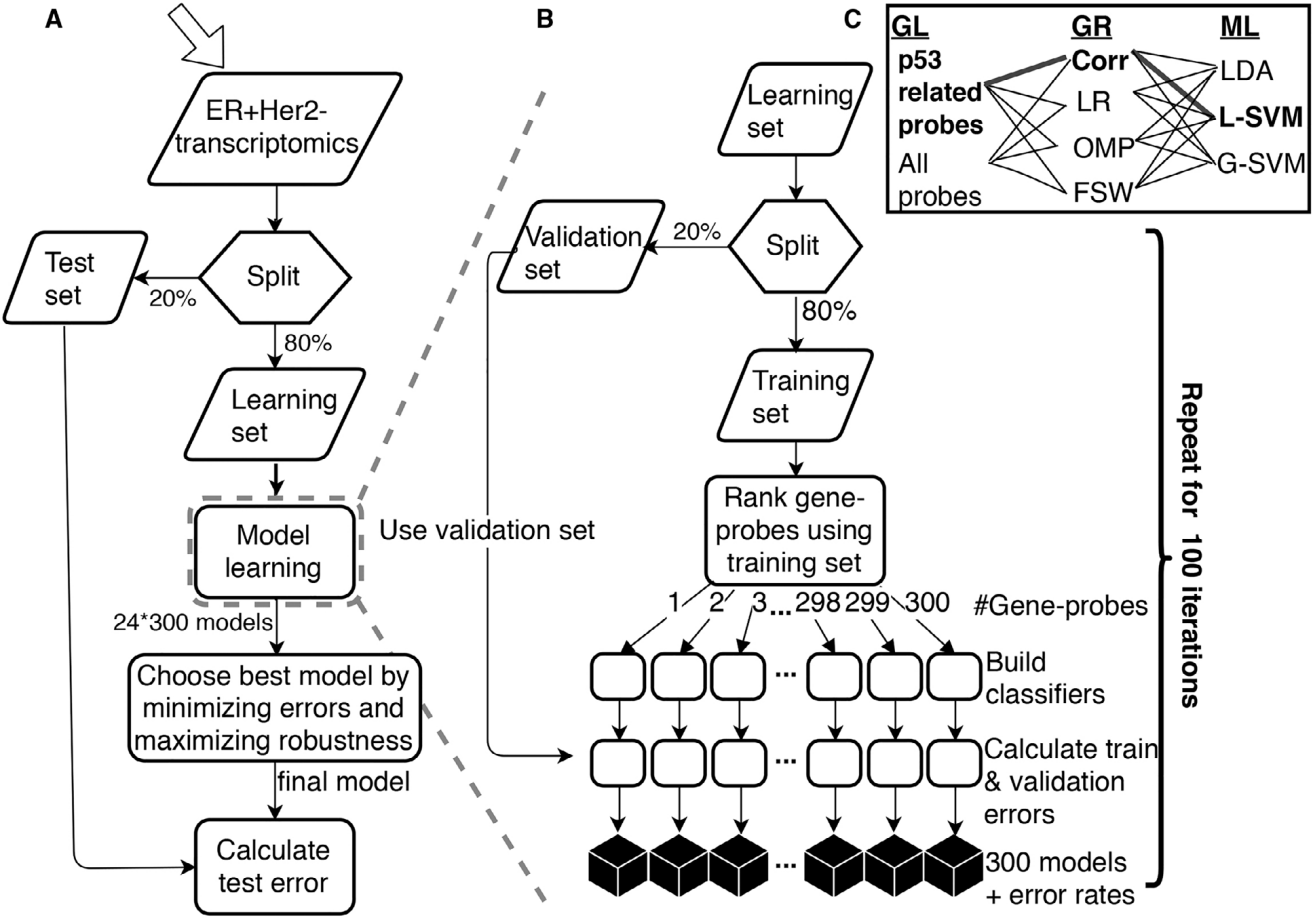
(A) Flowchart of the learning process to create a classifier of wt vs mut *TP53* samples. (B) Detailed description of the central learning module: For each one of the 100 iterations, we ran the learning process for every combination of C. GL (gene‐probe list) , GR (gene‐probe ranking), and ML (machine‐learning method). Each iteration uses a different learning set and corresponding ranked GL, generating a subclassifier of *k* = 1,2,…*N* ≤ 300 genes. Optimal GL*, GR*, ML*, and k* are selected on the basis of the validation error and robustness. The final model consists of the majority vote of C (70 ≤ *C* ≤ 100) subclassifiers based on k* genes and the selected GL*, GR*, and ML* methods. See text for detailed description.

The computational pipeline used to generate an optimal classifier of p53 status on the basis of expression data is presented in Fig. 1.

Step 1 is a 20–80% random split of the samples [separately for the METABRIC Discovery and Validation sets (Curtis *et al*., 2012)] to test and learning sets. The test set is used (only once!) to test the final classifier. The learning set is randomly split to validation (20%) and training (80%) sets. This is repeated 100 times (each split is referred to as an ‘iteration’).

Step 2 is feature selection: Since the number of samples available for learning is about one thousand, the number of variables (probes) used to fit these data must be reduced drastically to avoid overfitting. We started from two gene‐probe lists (GL): (a) knowledge‐based, of p53‐related genes (Allen *et al*., 2014; Schaefer *et al*., 2009) (425 probes, corresponding to 272 genes) and (b) using all 34,363 probes that correspond to 24,369 annotated genes. Next, four gene‐probe ranking (GR) methods were used to create a ranked GL (Fig. 1B): Linear regression (Mining, 2009), correlation (Corr)—ranking by the absolute value of the Pearson Corr coefficient between the gene probes’ expression and the *TP53* status of the learning samples; forward selection wrapper (FSW) (John *et al*., 1994), and orthogonal matching pursuit (Pati *et al*., 1993) (see Data S3). Only those *N* gene probes that passed Benjamini–Hochberg FDR (Benjamini and Hochberg, 1995) at *q* = 0.05 for the property used for ranking (e.g., Corr of expression with *TP53* status) were retained. For each iteration and each GL/GR combination, we generated a list of the top‐ranked *N* ≤ 300 probes. Ranking features is a notoriously unstable process—two slightly different training sets may produce very different lists of top‐ranked genes (Ein‐Dor *et al*., 2005, 2006). Repeating the process 100 times stabilizes the results of feature selection by ranking; robustness was tested according to several criteria (see Data S4, Fig. S1, and Table S2).

Step 3 is learning*:* for each iteration and its associated ranked GL, we learned the p53 labels, using three ML methods: linear discriminant analysis (Duda *et al*., 2001), linear support vector machine (LSVM), and Gaussian support vector machine (Mining, 2009).

Step 4 ‐ Model selection: For every iteration, each learning process (GL, GR, ML combination) was applied using the top‐ranked *k =* 1,2,3 *… N* probes, adding one probe at a time. We call a classifier ‘valid’ if all its *k* probes passed the threshold of FDR = 0.05 (e.g., *P*‐value for Corr). For each *k*, we required to have at least *C = *70 valid classifiers, for which the classification error was calculated for the training set and for the validation set, otherwise we stop at *k‐1*. Finally, the training and validation errors were averaged over the valid classifiers and plotted as a function of the number of probes used. These plots, together with considerations of robustness, were then used to determine the optimal learning process (GL*, GR*, ML*) and k*, the number of probes used, to yield the optimal set of subclassifiers.

Notably, the total number of classifiers that were constructed is on the order of 2×4×3×300×100 = 720,000 (in practice it is smaller; e.g., since the number of valid classifiers may be < 100, the number of probes that passed various filters may be < 300, and not all GL/GR/ML combinations were tested).

Step 5: The final master classifier uses, for a given sample, the expression levels of the k* probes of each of the *70 ≤ C *≤ 100 subclassifiers to produce *C* predicted p53 labels; the majority vote of these constitutes the final deduced p53 status.

Step 6: testing the master classifier. The samples of the test set were submitted to the master classifier and the test error was calculated. Tumors identified as mut are referred to as ‘Positive’; the error is the ratio of (false positives+false negatives)/(total number of classified samples).

Step 7: identification of the PM samples*:* FP samples from the test set were labeled PM. For the learning set, we relied only on misclassification of the validation samples, using the following rule: identify the iterations in which a wt sample was assigned to the validation set; if in the majority of these iterations, it was classified as mut—it is labeled PM.

### 2.5 Validating the classifier on an independent dataset—TCGA

The classifier derived on the basis of METABRIC data was tested on TCGA BC data (Koboldt *et al*., 2012). The TCGA dataset contains 98 mut and 381 wt *TP53* ER+Her2‐ samples, for which expression was measured using RNA‐seq. The manner in which the TCGA data were handled to account for different representations of genes in TCGA and METABRIC is described in Data S5.

### 2.6 Dimensionality reduction

To demonstrate intermixing of the PM group with mut samples, principal component analysis and tSNE (Van Der Maaten and Hinton, 2008) were used.

### 2.7 Characterizing the PM samples; Pathway‐level analysis

The METABRIC samples were studied also on pathway level, using Pathifier (Drier *et al*., 2013; Livshits *et al*., 2015). Lists of genes that belong to various pathways were downloaded from KEGG (Ogata *et al*., 1999), BioCarta (Nishimura, 2001), and the NCI‐Nature curated Pathway Interaction Database (Schaefer *et al*., 2009). All probes that appeared among the top 5000 varying ones (Drier *et al*., 2013) were used in the analysis; hence, some genes were represented by more than a single probe. For each pathway *P* and sample *k*, we derived *D*(*P*,*k*), a Pathway Deregulation Score (PDS), as described (Drier *et al*., 2013). The samples and pathways were sorted using SPIN (Tsafrir *et al*., 2005), to place together groups of similarly deregulated pathways and samples with similar deregulation profiles. Deregulation of pathways in the PM samples was compared to the mutants and to the wt (by sequence and classification), by two‐sided *t*‐tests and Benjamini–Hochberg FDR correction for multiple hypotheses. All the genes belonging to the pathways that were significantly differentially deregulated between the PM group and mutp53 samples were collected. For each corresponding gene‐probe expression, we calculated a two‐sided *t*‐test and FDR correction between the PM and the mutp53 groups.

### 2.8 Clinical characterization

Kaplan–Meier analysis of survival data of the three tumor types was performed by Matlab routine ‘ecdf’ for calculating the curve and ‘survdiff’ for calculating the *P*‐value of log‐rank test.

## 3 Results

### 3.1 The training process

We generated classifiers that distinguish wt *TP53* tumors from mut *TP53* tumors, based on their gene expression profiles. We present here results only for ER+Her2‐ tumors; for the other subtypes, see Data S6, Table S3, and Fig. S2. We assumed that if PM tumors indeed exist, they probably constitute a minority of the wt *TP53* cases, whereas most wt *TP53* cases retain a transcriptional profile distinct from that of true mut *TP53* tumors. For all tested combinations of gene lists (GL), gene ranking (GR), and ML, 100 iterations were performed. Classifiers based on *k* gene probes were constructed by adding one probe at a time, according to their ranking. Note that for ER+Her2‐, we stopped at *k = *138 probes; beyond this, the number of valid classifiers was < 70. For each *k*, GL, GR, and ML combination, the average and standard deviation of the error (training and validation) over the 100 iterations was stored. These error curves, together with considerations of their robustness (Data S4), served to select the optimal classification model (see Data S7 and Fig. S3). The selected model for ER+Her2‐ tumors is presented in Table 2; it uses the p53‐related gene list as GL*, gene probes ranked by Corr to p53 status as GR*, and LSVM as the ML* method of choice. The fact that the p53‐related GL separates well wt *TP53* tumors from those that carry *TP53* mutations supports our assumption that the majority of the former tumors retain a wtp53‐driven transcriptional program.

**Table 2:** Details of the final classifier for ER+Her2‐ tumors and the classification errors for METABRIC and TCGA data (see text).

#Training samples	#Validation samples	#Test samples	#probes k* (mean ± SEM genes)	Selected GL* (GR*)	Selected ML* method	Validation error mean ± SEM	Training error mean ± SEM	Test error (majority vote)	TCGA test error (majority vote)
878	220	275	62 (49.3 ± 0.01)	p53‐related genes (Corr)	Linear SVM	0.116 ± 0.002	0.0868 ± 0.006	0.13	0.11

**Fig. 2:**
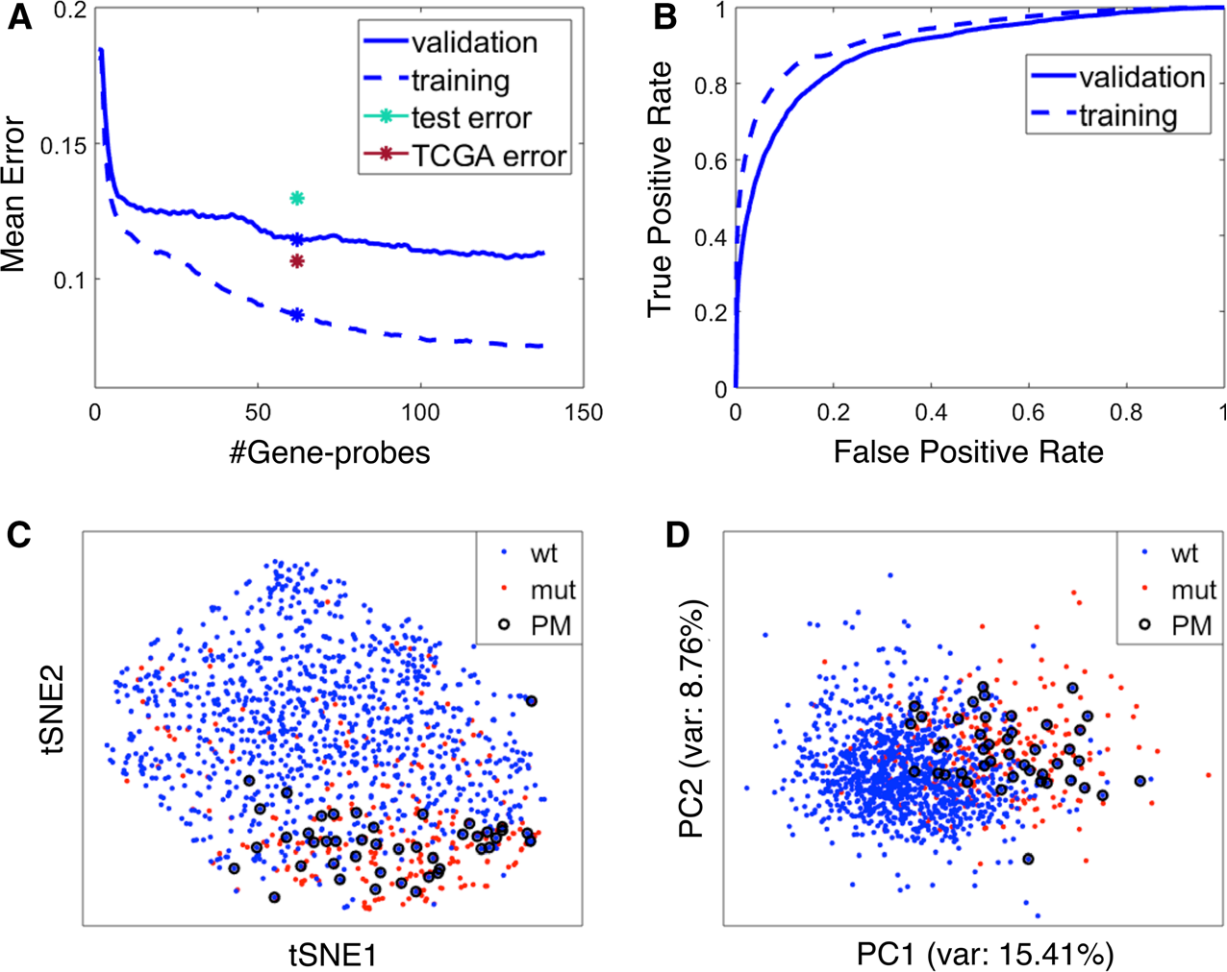
Accuracy and error rates for the chosen model for ER+Her2‐ tumors. (A) Mean error rates of prediction of *TP53* status (mut/wt) for the validation and training sets, using the chosen ML, GR, and GL, for 100 iterations, vs the number of top‐ranked gene probes used. The test (METABRIC) and the TCGA errors of the final model (with 62 gene probes) are marked by green and red asterisks, respectively. The mean validation and training errors of the selected model are marked by blue asterisks. (B) Mean ROC curves for the final model for both validation and training sets. The area under the curve values for the validation curve and the training curve are 0.89 and 0.93, respectively. (C) tSNE dimensional reduction from the space of the 62 most used gene probes (see text) to 2 dimensions. Positions of wt (blue), mut (red) and PM (circled) show that the PM tumors are intermixed with the mut tumors. (D) Same as C, using PCA.

The mean errors (of 70 ≤ *C* ≤ 100 classifiers) plotted as a function of gene‐probe number *k*, and the mean receiver operating characteristic (ROC) curves of the optimal classifiers, are presented in Fig. 2A,B, respectively. We decided to use k* =62 probes, representing, on average, 49.3 ± 0.1 genes (mean ± SEM); this yielded a mean ± SEM validation error of 0.116 ± 0.002 (Table 2). Shorter GLs are more robust, and at 62 probes, we had 100 valid classifiers. Hence, we preferred k* = 62, even though the validation error was slightly higher than for 125 probes (Fig. 2A). The list of 62 probes used for each of the final 100 subclassifiers is in Appendix S3. The ROC curve presented is the average (over all the 100 classifiers) of the ROC curves that were obtained separately by Matlab routine ‘perfcurve’ for each classifier.

### 3.2 Identifying ‘pseudomutant’ p53 tumors

The final wt/mutp53 call, for a previously unseen sample (e.g., from the test set), is obtained as a majority vote of the 100 subclassifiers (Data S8, Table S4, and Fig. S4). The test error of this final classifier was 0.13 (Table 2): Out of 224 wt *TP53* samples, 14 were misclassified as mut and were defined as PM. Of note, since we decided not to correct for the unbalanced learning data (see Materials and methods), our classifier is strongly biased toward wt calls and against mut calls; as a result, out of the 51 mut *TP53* test samples, 22 were falsely classified as wt (false negative, FN). When the imbalance is corrected, this error rate is reduced to 12/51. Appendix S4 lists the FN cases; remarkably, NULL samples were under‐represented in this group, with *P*‐values of 0.043 and 0.1464 (hypergeometric test) for the final classifier and the imbalance‐corrected classifier, respectively, raising the interesting possibility that, in some tumors, mutp53 proteins retain partial wtp53 activity.

We then returned to the training set, to identify additional PM tumors (see Materials and methods, Step 7). Altogether, we found 30 such additional cases (out of 898 wt *TP53* training samples), so that in total 44 out of 1122 (about 4%) wt *TP53* ER+Her2‐ tumors were identified as PM (for patient IDs, see Table S5). This fraction of 4% is very robust: A similar percentage was obtained independently of the GL, GR, and ML methods used (as defined in Constructing the wt/mutp53 classifier) (Data S9 and Table S8). Of note, our classifier is conservative, with a bias against mut p53 calls; hence, the frequency of PM cases may actually be higher. Indeed, a larger number of samples (19 from the test set and 76 from the validation sets, Data S2) were identified as PM when we used a learning process (Table S6) that compensates for the unbalanced learning set (Data S2), bringing the percentage of PM cases up to about 8.5%.

Next, we ranked the gene probes according to the number of their appearances among the top 62 of the 100 iterations and used the top‐ranked 62 probes to represent each sample. Dimensionality reduction to two dimensions (tSNE and PCA) was then applied (Fig. 2C,D). Remarkably, the great majority of PM samples projected onto ‘mut territory’ and were intermixed with authentic mut *TP53* tumors, further displaying the similarity between the transcriptional profiles of the two groups.

It is important to rule out the possibility that misclassification of wt as mut may have been due to questionable sequencing data. In that regard, we note that 34 of the 44 PM tumors were sequenced by both SS and NGS. Of those, 30 were called as wt *TP53* by both methods. Only for four was there a discrepancy; they were called wt by NGS but mut by SS. Of the remainder 10 wt *TP53* cases, eight were sequenced only by NGS and two only by SS (Table S7). The probability of mistakenly identifying a sample as wt by two independent methods is very low, giving good reason to trust that the PM tumors are not mis‐sequenced *TP53* mutants. Deeper curation of the four discordant tumors indicated that three of these (one from the test set and two from the learning set, see Data S10) were complex frameshifts, seen in SS chromatograms but missed by the NGS indel mutation caller. A fourth case had a single indel read in NGS. Importantly, the potential misclassification of this very small number of tumors does not affect our conclusions.

In all but one of the PM cases, mutations were identified in other sequenced genes (Appendix S5). This strongly suggests that the cellularity and sequencing quality of those samples should have been sufficient to detect *TP53* coding region mutations, had they existed in those samples.

### 3.3 ‘Pseudomutants’ in TCGA data

The TCGA ER+Her2‐ BC dataset contains 98 mut and 381 wt *TP53* samples. Gene expression was measured by RNA‐Seq, and some of the METABRIC genes we used for classification had no reported expression levels in TCGA. Consequently, only 42 out of the 100 subclassifiers had TCGA expression data for all their genes, and the majority vote of these 42 subclassifiers was used for wt/mut *TP53* calling. Nevertheless, the TCGA error rate of 0.11 is even lower than that of the METABRIC test error. Through this analysis, 15 PM samples were identified in TCGA, which again constitutes about 4% of all wt *TP53* samples. Thus, our classifier works for a different set of patients, with expression measured by a different platform; this constitutes strong evidence for the robustness of our method. The similar percentage of PM cases indicates that their identification is not an artifact of one particular dataset.

### 3.4 Differential expression of the main gene probes of the classifier between wt and mutp53

Figure S5 shows the expression levels of the main genes (and gene probes) which were used in the final classifier for the ER+HER2‐ subtype, for the mutp53, wtp53, and PM samples. We show separately results for up and downregulated gene probes (in mut vs wt p53) and for each cohort (METABRIC/TCGA). For the ER‐Her2‐ and Her2+ subtypes, expression levels of the corresponding main gene probes are shown in Figs S6 and S7 for the wtp53 and mutp53 samples. For each case tested, the difference in expression between wt and mutp53 was statistically significant.

The list of these main genes and gene probes is in Appendix S6; the manner in which they were selected is described in Data S11.

### 3.5 Pathway deregulation in the PM tumors

**Fig. 3:**
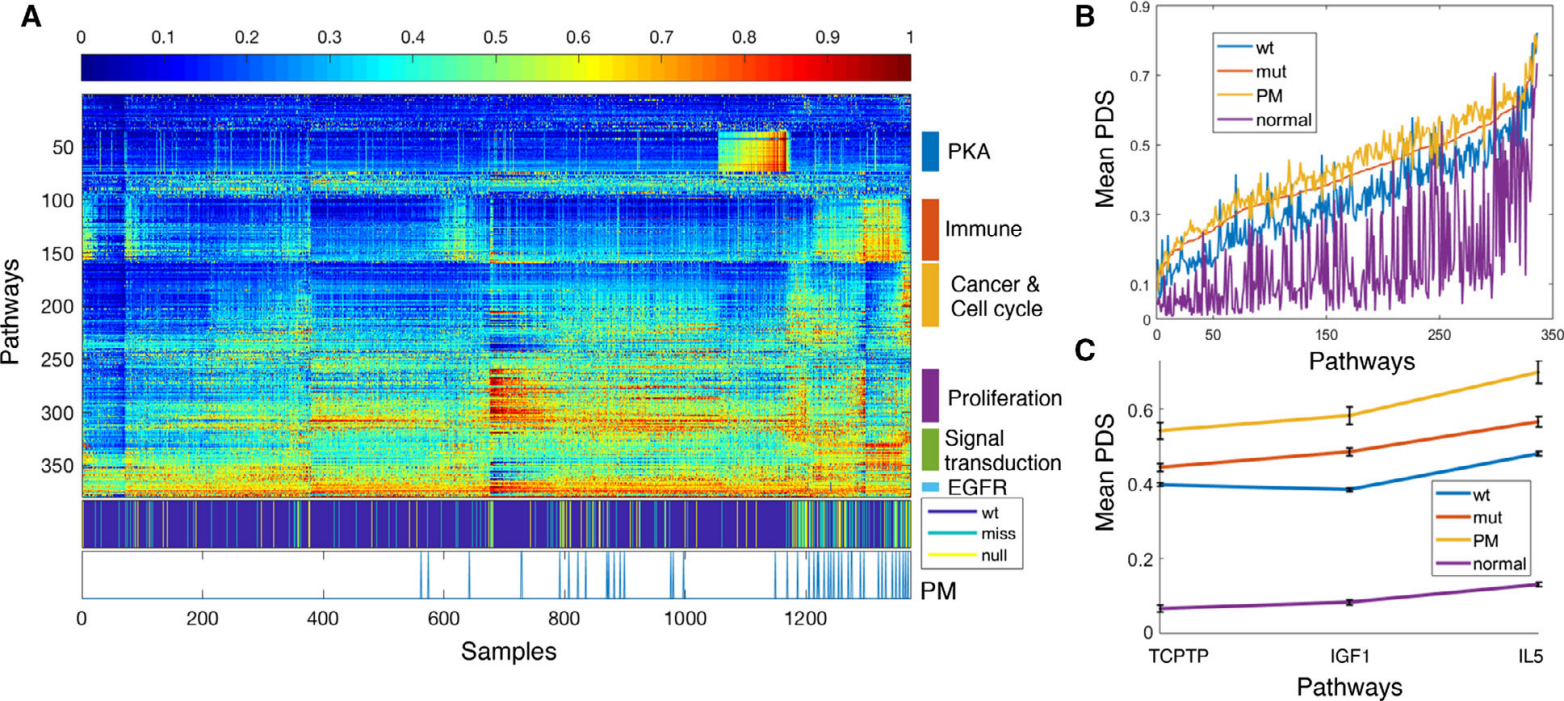
Pathway deregulation‐based analysis of ER+Her2‐ tumors. (A) Heatmap of the PDS of 1373 tumors and 144 normal breast samples, encompassing 379 pathways. Samples and pathways were ordered by SPIN, placing in proximity objects of similar deregulation profiles. The second color bar from the bottom identifies *TP53* mutation status: wt, missense, and null. The bar below it identifies the PM samples; remarkably, these samples cluster in the region dominated by mutants. Different pathway clusters are identified on the right side. (B) Mean PDS of 336 pathways that were differentially deregulated in PM vs wtp53 tumors. Pathways were ordered by their mean PDS in the mut samples. (C) Mean PDS of normal breast tissue and of the wtp53, mutp53, and PM groups, for three pathways that are differentially deregulated between PM and mutp53 tumors.

Moving from single gene‐based transcriptomes to pathway level, we combined the expression profiles the 1373 ER+Her2‐ tumors with those of 144 normal breast samples and calculated the PDS (Drier *et al*., 2013; Livshits *et al*., 2015) of each sample for 379 pathways that passed a threshold of stability. The heatmap of these scores is presented in Fig. 3A. Both samples and pathways are ordered in a manner that places in proximity objects with similar PDS profiles (Tsafrir *et al*., 2005). Again, most PM samples were assigned to a region preferentially occupied by mut *TP53* tumors, indicating that their pathway deregulation profiles resemble those of these tumors.

Next, we looked for pathways displaying significantly different mean PDS in PM vs the rest of the wt *TP53* tumors. Out of 574 tested pathways, 336 passed as differentially deregulated in PM vs wtp53 at an FDR of 0.05. Ordering these pathways by the mean PDS of the mut *TP53* samples, we plotted the mean PDS of the PM, mutp53, and wtp53 tumors and of normal mammary tissue samples (Fig. 3B). Interestingly, the PDS of the wtp53 tumors were closest to normal tissue, while those of the mutp53 cases were higher. Remarkably, the PM tumors had the highest mean pathway deregulation: Nearly all 336 pathways had a higher mean PDS for the PM than for the mut *TP53* cases, indicating an extremely significant overall deregulation difference (on a multipathway level). Thus, at least by PDS assessment, the PM tumors appear to behave as even ‘more mutant’ than true mutants.

**Fig. 4:**
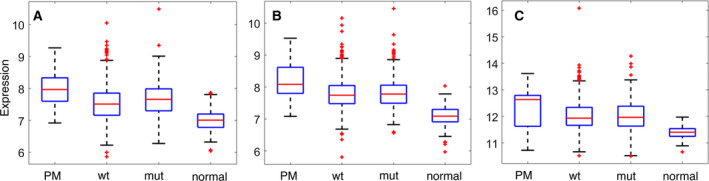
Mean (log) expression values of GRB2 for the different groups of samples. (A) METABRIC, probe ILMN 1742597, PM vs mut; *P* = 0.00035; (B) METABRIC, probe ILMN 1748721, PM vs mut; *P* = 0.00003; (C) TCGA, PM (*n* = 15 samples) vs mut; *P* = 0.11.

Deregulation of cancer‐related pathways can cause wtp53 to adopt mut‐like behavior (Furth *et al*., 2015). We therefore looked for pathways that are preferentially deregulated in the PM samples, relative to mutp53 breast tumors, and therefore may potentially drive wtp53 protein into a PM state. Using an FDR threshold of 0.1, we found three such pathways (Fig. 3C): the IGF1 (BIOCARTA), IL5, and TCPTP pathways (NCI). Altogether, these three pathways comprise 67 genes. When each of the corresponding gene probes was tested individually for differential expression between the PM and mutp53 groups, only two probes, both representing *GRB2*, were significantly differentially expressed. Growth Factor Receptor Bound Protein 2 (GRB2) is an adaptor protein that links growth factor receptors to the Ras signaling pathway. As seen in Fig. 4, its mean expression, in both METABRIC and TCGA, was indeed higher in the PM tumors than in the mut or wtp53 tumors.

### 3.6 Clinical features of the PM tumors

**Fig. 5:**
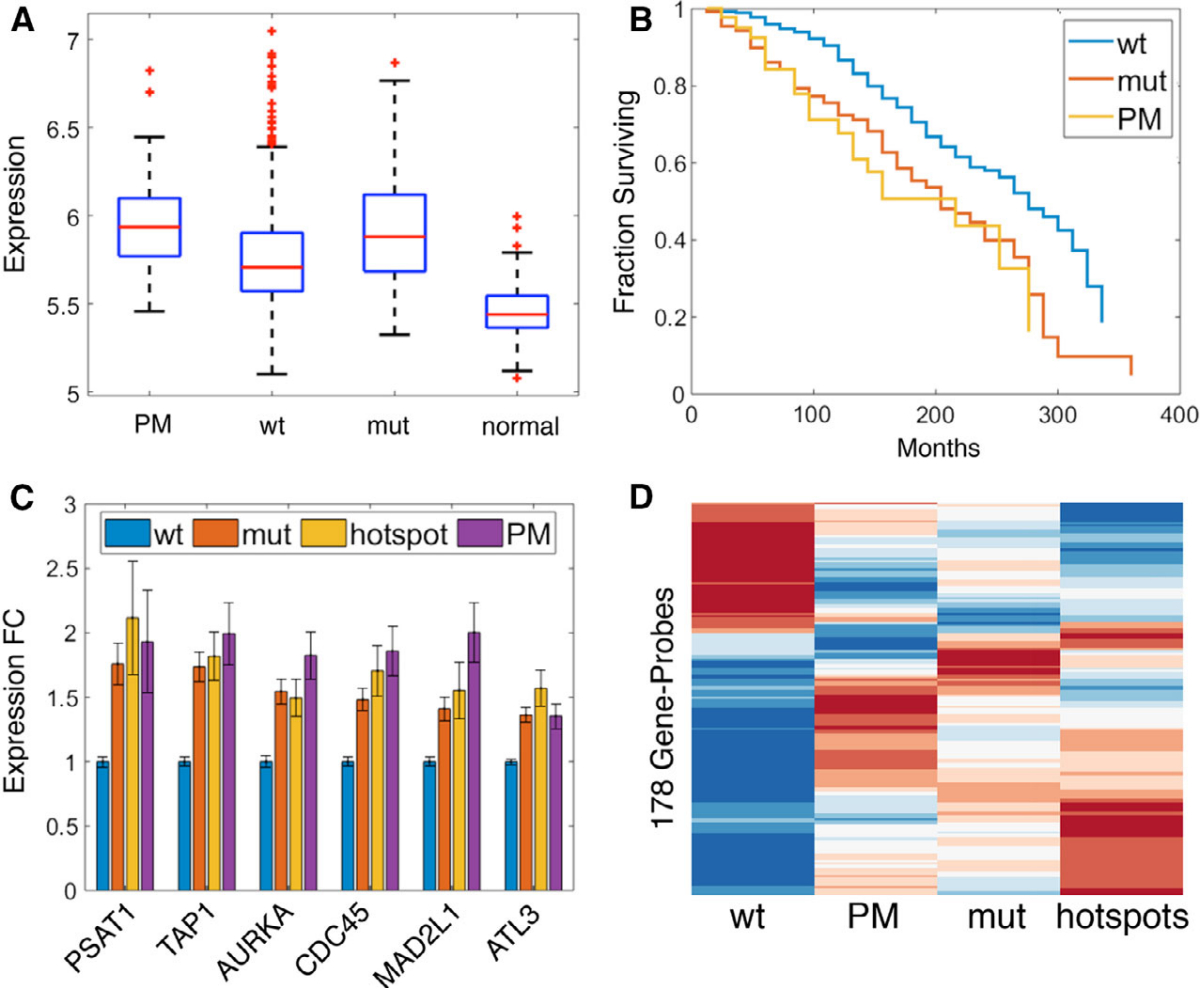
(A) Expression levels of MKI67 in PM, wt, and mutp53 ER+Her2‐ breast tumors and in normal breast tissue samples. (B) Kaplan–Meier survival curves for BC patients bearing PM, wt, or mutp53 ER+Her2‐ tumors, with *P*‐value = 0.00276, calculated by log‐rank test, between the survival of PM vs wt. (C) Expression fold change values in the indicated tumor groups, relative to wtp53 tumors, of six mut p53‐regulated genes. ‘Hotspots’ indicates tumors harboring one of the 10 most common *TP53* hotspot mutations, according to the IARC mutation database (Hainaut *et al*., 1998). (D) Mean expression of 178 gene probes corresponding to mut p53‐regulated genes (Walerych *et al*., 2016) that passed FDR < 0.05 between wt and mutp53, displayed for the wt, PM, mut, and p53 hotspots groups of samples. The gene probes were ordered by hierarchical clustering, using the ‘clustergram’ Matlab function.

MKI67 mRNA levels, indicative of cell proliferation, were significantly higher (*P* = 1.5 × 10^−5^) in the PM group than in the wtp53 group, resembling those of mutp53 samples (Fig. 5A). Hence, PM tumors possess higher average proliferation rates than the remainder of the wt *TP53* tumors (Fig. 5A). Moreover, the prognosis of BC patients with ER+Her2‐ PM tumors is significantly worse than that of patients with ‘conventional’ wtp53 tumors, and similar to that of patients with authentic mut *TP53* tumors, as assessed by overall survival (Fig. 5B) and distant relapse‐free survival (Fig. S9A). In agreement, patients with PM tumors tend to be associated with a greater number of positive lymph nodes than those with wt *TP53* tumors (Fig. S9B). Hence, PM tumors resemble *bona fide* mut *TP53* tumors not only in gene expression and pathway deregulation patterns, but also in clinical features.

Lastly, since common p53 missense mutants can exert oncogenic GOF effects, we asked whether PM p53 may also possess GOF activities in BC. To demonstrate p53 GOF formally, one needs to show that depletion of the tested p53 protein attenuates cancer‐related features. Obviously, this is impractical for archived tumor specimens. Therefore, we took advantage of a study of *TP53*‐mutated BC cell lines (Walerych *et al*., 2016). Silencing the endogenous mutp53, followed by RNA‐Seq analysis, identified a core group of 205 genes that are specifically regulated by mutp53 and contribute to its GOF in BC (Walerych *et al*., 2016). These 205 genes are represented in METABRIC by 337 probes; 178 of these probes separate wt from mut *TP53* cases at FDR < 0.05 (see Fig. 5D for heatmap of their expression for all samples), while 104 separate PM from the other wt cases (with 88 probes shared between these two lists). In Fig. 5C, we display results for the six genes with the highest fold change between the mutp53 and the wtp53 ER+Her2‐ samples in the METABRIC dataset. As expected, all six genes were expressed more abundantly in the mutp53 tumors compared to wt tumors; in most cases, their expression was slightly higher in tumors with hotspot p53 mutations [10 most frequent *TP53* mutations in IARC mutation database (Hainaut *et al*., 1998)], consistent with their proposed contribution to mutp53 GOF. Notably, expression of those genes in the PM tumors was at least as high as in hotspot mutp53 tumors (Fig. 5C). This raises the intriguing possibility that in tumors where wtp53 acquires PM features, it not only loses its tumor suppressive activities but may even gain cancer‐promoting activities.

## 4 Conclusions

Breast cancer is a heterogeneous disease, with diverse subtypes, each driven by distinct molecular and genetic mechanisms. We developed classifiers, separately for each BC subtype, which differentiate well between tumors that retain wtp53 expression and those that have undergone *TP53* mutations. To avoid the confounding effect of the marked differences in percentages of mutp53 in the different subtypes, each subtype must be analyzed as a separate group. The large number of samples in the METABRIC study, with available expression data (Curtis *et al*., 2012), *TP53* sequencing (Pereira *et al*., 2016; Silwal‐Pandit *et al*., 2014), and clinical information enable statistical robustness of subtype‐specific analysis. For the largest group, of ER+Her2‐ patients, even though 275 tumors were set aside as a test set, the number of tumors that remained available to be used for learning was large enough for reliable training and validation. By combining 100 repeats of the entire training process, each with its own random training/validation split, we overcame the lack of stability of ranked GLs that has plagued most single‐gene‐based classifiers (Ein‐Dor *et al*., 2005). We derived a very robust expression‐based classifier, which separated successfully mut from wt *TP53* tumors also in the independent TCGA cohort of patients (Koboldt *et al*., 2012).

Using this approach, we identified a group of ER+Her2‐ patients whose tumors harbor wt *TP53* but display a mut p53‐associated transcriptional program. These ‘PM p53’ tumors resemble authentic *TP53*‐mutated ER+Her2‐ breast tumors not only in their gene expression and pathway deregulation profiles, but also in their highly proliferative nature and shorter patient survival.

What may cause a wt *TP53* tumor to acquire a PM transcriptional profile? Potentially, such an outcome might be obtained by complete loss of p53 expression, rendering these tumors practically p53‐null (Miller *et al*., 2005). However, although we saw a slight reduction in p53 mRNA levels in our PM group relative to the rest of the wtp53 samples (Fig. S8), p53 expression was significantly higher in the PM tumors than in tumors carrying nonsense/frameshift mutations (*P*‐value = 0.0022, two‐sided *t*‐test). Thus, additional mechanisms are likely to contribute to the PM behavior. These might involve deregulation of cancer‐relevant signaling pathways; for example, it was previously shown that deregulation of the Hippo pathway can alter the functionality of wtp53 (Furth *et al*., 2015). Indeed, pathway‐level comparisons (Drier *et al*., 2013) demonstrated that hundreds of biological pathways are significantly more deregulated in PM than in wtp53 tumors. Interestingly, elevated expression of *GRB2* was particularly symptomatic of PM tumors. GRB2 is involved in relaying signaling downstream to numerous growth factor receptors. Hence, although causality between high GRB2 and PM p53 behavior remains to be proven, it resonates well with the early observation that wtp53 protein can be shifted into a mut‐like conformation upon growth factor stimulation (Zhang and Deisseroth, 1994). Additionally, epigenetic changes may also contribute to acquisition of a PM p53 phenotype, as suggested by the mut‐like behavior of wtp53 in cancer‐associated fibroblasts (Arandkar *et al*., 2018).

Elucidation of the molecular mechanisms that drive wtp53 into a PM state may identify potential treatments that can restore wtp53 functionality in tumors harboring PM p53. This may selectively benefit patients whose tumors display PM features in association with bad prognosis. Thus, further elaboration of the underlying mechanisms is highly desirable.

## Conflict of interest

The authors declare no conflict of interest.

## Author contributions

GB, ED, and MO designed research; GB performed research; GF provided computational advice, S‐FC, OMR, and CC provided insights about the data; SM and SA performed RNA quantification; ED, MO, and YA conceived the study; ED and MO provided computational and biological guidance, respectively. GB, ED, and MO wrote the paper.

## Consent to publish

The authors are fully responsible for the contents of this manuscript, and the views and opinions described in the publication reflect solely those of the authors.

## Supporting information


**Appendix S1**. Final expression table of 34,363 (HT‐12 v3 platform, Illumina_Human_WG‐v3) gene‐probes representing 24,369 genes, for 1928 tumor and 144 normal samples.

**Appendix S2**. TP53 labels, the mutation types and breast cancer subtypes (ER+Her2‐, ER‐Her2‐ and Her2+, as determined by Immunohistochemistry), protein change, survival and treatment information and clinical parameters.

**Appendix S3**. List of probes used for each of the final 100 subclassifiers.

**Appendix S4**. List of False Negative cases.

**Appendix S5**. Mutations identified in other sequenced genes.

**Appendix S6**. List of main genes and gene probes used in the classifier for each breast cancer subtype.

**Data S1**. Pre‐processing.
**Data S2**. Imbalanced Learning.
**Data S3**. Forward Selection Wrapper and Orthogonal Matching Pursuit.
**Data S4**. Robustness measurements for both genes and lists.
**Data S5**. TCGA pre‐processing.
**Data S6**. Results of the learning process for ER‐Her2‐ and for Her2+.
**Data S7**. Additional error curves for ER+Her2‐ tumors.
**Data S8**. Additional information about pseudomutant classification for ER+Her2‐ samples.
**Data S9**. Comparison of the number of ER+Her2‐ pseudomutant (PM) samples using varying combinations of ML, GL and GR methods.
**Data S10**. Discrepancy between NGS and Sanger sequencing for TP53 mutations for the PM samples.
**Data S11**. Comparison of the expression of the main gene‐probes, used by the final classifier, between the wtp53 and mutp53 groups.
**Table S1**. Samples suspected to have germline mutations (*n* = 15).
**Table S2**. Robustness of the final model’s gene lists (ER+Her2‐ using p53 related genes). The mean and std of the intersection and the total number of genes appearing among the 100 lists of top 62 ranked probes are displayed.
**Table S3**. The final constellation of ML*, GR*, GL* and k* for ER‐Her2‐ and Her2+and the corresponding error rates.
**Table S4**. The number of ER+Her2‐ samples that were falsely classified at least once during the training phase and the validation phase of the final classifiers. The numbers of mut and wt samples, and percentage of mutants at the training stages (without test data), are shown in the three right columns.
**Table S5**. PM samples (from ER+Her2‐) that were recognized by the final classifier both METABRIC and TCGA datasets.
**Table S6**. Chosen parameters for the final imbalance‐corrected model for ER+Her2‐ and its corresponding errors (training, validation, test, and TCGA), with the number of models having all required genes in the TCGA (out of the 100 subclassifiers).
**Table S7**. Discrepancies between NGS and Sanger sequencing for the 44 pseudomutant (PM) samples.
**Table S8**. About 4% incidence of PM tumors was obtained independently of the GL, GR and ML methods used. The number of FP samples and the percentage of the FP group, revealed using 6 different combinations of ML, GL GR is presented below for ER+Her2‐ subtype. The first line displays the results of the final classifier that was used in the paper.
**Fig**.** S1**. Number of appearances of each gene amongst the 62 top ranked probes, chosen from the p53 related gene list to be used in at least one final subclassifier, during the 100 iterations for ER+Her2‐ tumors.
**Fig**.** S2**. Accuracy and error rates for the chosen models for ER‐Her2‐ and Her2+tumors.
**Fig**.** S3**. Additional error rates for ER+Her2‐ tumors, each based on 100 iterations (and therefore 100 error rates).
**Fig**.** S4**. The number of wtp53 ER+Her2‐ samples from the validation set, which were misclassified as false positive (mutp53 classification) at least once (*n* = 81).
**Fig**.** S5**. Mean expression of the main gene probes (see SI appendix 10) used by the final classifier for predicting p53 state, for ER+Her2‐ subtype in METABRIC (panels A&B) and TCGA (panels C&D) cohorts, shown for the PM, wt and mutp53 groups.
**Fig**.** S6**. Mean expression of the main gene probes (see SI appendix S10) used by the final classifier for predicting p53 state, for ER‐Her2‐ subtype in METABRIC (panels A&B) and TCGA (panels C&D) cohorts, for the wt and mutp53 groups.
**Fig**.** S7**. Mean expression of the main gene probes (see SI appendix 10) used by the final classifier for predicting p53 state, for Her2+subtype in both METABRIC (panels A&B) and TCGA (panels C&D) cohorts, for the wt and mutp53 groups.
**Fig**.** S8**. Comparison of TP53 mRNA expression in the different groups of ER+Her2‐ tumors and in normal human breast tissue.
**Fig**.** S9**. ER+Her2‐ PM patients tend to present with a higher number of positive lymph nodes than wtp53 patients and have a lower probability for relapse‐free survival.

## Data Availability

All data are available in the Appendix.
