# Supplementary material for: Transcriptional profiling reveals a subset of human breast tumors that retain wt TP53 but display mutant p53‐associated features
Source: Mol Oncol. 2020 Jun 23;14(8):1640–52. doi: 10.1002/1878-0261.12736 (PMC7400784; doi:10.1002/1878-0261.12736)
Supplement: Supplementary file 1 — Appendix S1. Final expression table of 34,363 (HT‐12 v3 platform, Illumina_Human_WG‐v3) gene‐probes representing 24,369 genes, for 1928 tumor and 144 normal samples. [file MOL2-14-1640-s001.docx]

**Supplemental File 1**

Final expression table of 34,363 (HT-12 v3 platform, Illumina_Human_WG-v3) gene-probes representing 24,369 genes, for 1928 tumor and 144 normal samples.

The data file is too large to upload onto the website of the journal: it is available upon request from the authors.
