# Supplementary material for: Transcriptional profiling reveals a subset of human breast tumors that retain wt TP53 but display mutant p53‐associated features
Source: Mol Oncol. 2020 Jun 23;14(8):1640–52. doi: 10.1002/1878-0261.12736 (PMC7400784; doi:10.1002/1878-0261.12736)
Supplement: Supplementary file 2 — Appendix S2. TP53 labels, the mutation types and breast cancer subtypes (ER+Her2‐, ER‐Her2‐ and Her2+, as determined by Immunohistochemistry), protein change, survival and treatment information and clinical parameters. [file MOL2-14-1640-s002.pdf]

Supplemental File 2

TP53 labels, the mutation types and breast cancer subtypes (ER+Her2-, ER-Her2- and Her2+, as determined by Immunohistochemistry), protein change, survival and treatment information and clinical parameters.

Row	MB_0362	MB_0346	MB_0386	MB_0574	MB_0185	MB_0503	MB_0641	MB_0201	MB_0218	MB_0316	MB_0189	MB_0891
p53_labels	WT	MUT	MUT	MUT	MUT	WT	WT	MUT	WT	MUT	WT	WT
mutation_type	WT	MISS	NULL	MISS	MISS	WT	WT	MISS	WT	MISS	WT	WT
protein_change		R273C	E349Gfs*30	Y236C	p.P278S			G245D		P151S		
subtype	ERpHER2n	HER2p	ERpHER2n	ERpHER2n	ERpHER2n	ERpHER2n	ERpHER2n	HER2p	ERpHER2n	ERnHER2n	ERpHER2n	ERpHER2n
survival_status	Died of Disease	Died of Disease	Living	Living	Died of Disease	Living	Living	Living	Living	Living	Died of Disease	Living
survival_months	47.03333333	20.43333333	138.1333333	119.8	43.83333333	101.2333333	102.5666667	125.7	131.0666667	182.9	9.066666667	149.4
chemotherapy	YES	NO	NO	NO	NO	NO	NO	YES	YES	NO	YES	NO

MB_0658	MB_0899	MB_0605	MB_0258	MB_0506	MB_0420	MB_0223	MB_0445	MB_0199	MB_0517	MB_0155	MB_0428	MB_0117
MUT	WT	WT	WT	MUT	MUT	WT	WT	WT	WT	WT	WT	WT
MISS	WT	WT	WT	MISS	NULL	WT	WT	WT	WT	WT	WT	WT
R248Q				C135W	R306*							
ERnHER2n	ERpHER2n	ERpHER2n	ERpHER2n	ERnHER2n	ERnHER2n	ERpHER2n	ERpHER2n	ERpHER2n	ERpHER2n	ERpHER2n	ERpHER2n	ERpHER2n
Living	Living	Living	Living	Died of Disease	Living	Died of Disease	Living	Living	Living	Living	Living	Living
97.26666667	175.8	114.6	86.13333333	45.8	76.73333333	81.33333333	132.5333333	144.4	61.9	59.8	55.93333333	2.4
YES	YES	NO	NO	YES	YES	YES	YES	NO	NO	NO	NO	NO

MB_0906	MB_0249	MB_0660	MB_0497	MB_0434	MB_0143	MB_0513	MB_0541	MB_0653	MB_0455	MB_0540
MUT	MUT	MUT	WT	WT	WT	WT	WT	MUT	MUT	WT
MISS	MISS	MISS	WT	WT	WT	WT	WT	MISS	NULL	WT
L111P	Y205S	C141Y						E285K	S166*	
ERnHER2n	ERnHER2n	ERnHER2n	ERpHER2n	HER2p	ERpHER2n	ERpHER2n	ERpHER2n	ERnHER2n	ERpHER2n	ERnHER2n
Died of Other Causes	Living	Died of Disease	Living	Died of Disease	Died of Other Causes	Living	Living	Died of Disease	Living	Living
63.86666667	188.3333333	18.93333333	73.7	45.5	54.33333333	117.0333333	174.2666667	24.8	99.33333333	194.5333333
NO	NO	YES	NO	YES	NO	NO	YES	NO	NO	NO

MB_0384	MB_0637	MB_0157	MB_0443	MB_0584	MB_0292	MB_0322	MB_0501	MB_0401	MB_0140	MB_0606
WT	MUT	MUT	WT	WT	MUT	WT	MUT	MUT	WT	WT
WT	MISS	MISS	WT	WT	NULL	WT	MISS	NULL	WT	WT
	V272G	Y163C			I195Yfs*14		P27L	D148Pfs*21		
ERpHER2n	ERpHER2n	ERnHER2n	ERpHER2n	ERpHER2n	ERnHER2n	ERpHER2n	ERpHER2n	ERnHER2n	ERpHER2n	ERpHER2n
Died of Disease	Living	Living	Living	Living	Died of Other Causes	Died of Other Causes	Living	Died of Disease	Living	Died of Disease
61.1	80.66666667	114.7666667	45.03333333	118.2	49.43333333	50.53333333	71.5	26.26666667	147.9333333	27.46666667
NO	NO	NO	NO	NO	NO	NO	YES	YES	NO	NO

MB_0666	MB_0598	MB_0453	MB_0138	MB_0579	MB_0471	MB_0347	MB_0619	MB_0171	MB_0310	MB_0621	MB_0614
WT	WT	MUT	WT	WT	WT	MUT	WT	WT	WT	WT	WT
WT	WT	NULL	WT	WT	WT	NULL	WT	WT	WT	WT	WT
		Q165*				p.E339X					
ERpHER2n	ERpHER2n	ERnHER2n	ERpHER2n	ERpHER2n	ERpHER2n	ERpHER2n	ERpHER2n	ERpHER2n	ERpHER2n	ERpHER2n	ERpHER2n
Died of Disease	Died of Disease	Died of Disease	Living	Died of Other Causes	Living	Died of Disease	Living	Living	Living	Living	Living
24.33333333	89.36666667	43.13333333	150.5666667	92.76666667	107.4666667	205.5666667	94.23333333	5.433333333	185.1666667	33.56666667	119.3333333
YES	NO	YES	NO	NO	NO	YES	YES	NO	NO	NO	YES

MB_0372	MB_0374	MB_0382	MB_0327	MB_0066	MB_0144	MB_0596	MB_0164	MB_0215	MB_0146	MB_0229	MB_0505
MUT	WT	MUT	WT	WT	WT	WT	MUT	WT	WT	WT	WT
NULL	WT	MISS	WT	WT	WT	WT	MISS	WT	WT	WT	WT
E285*		R337L					R175H				
ERnHER2n	ERpHER2n	ERpHER2n	ERpHER2n	ERpHER2n	ERpHER2n	ERpHER2n	ERnHER2n	ERpHER2n	ERpHER2n	ERpHER2n	ERpHER2n
Died of Disease	Living	Living	Living	Living	Died of Disease	Died of Other Causes	Living	Living	Died of Other Causes	Living	Living
62.76666667	1.433333333	136.4666667	187.9333333	157.4333333	152.0666667	100.4666667	10.83333333	122.2	86	71.5	60.7
YES	NO	YES	NO	NO	NO	NO	YES	NO	NO	NO	NO

MB_0102	MB_0569	MB_0516	MB_0272	MB_0585	MB_0494	MB_0306	MB_0463	MB_0198	MB_0203	MB_0607
MUT	WT	MUT	WT	MUT	MUT	MUT	WT	WT	WT	MUT
MISS	WT	MISS	WT	MISS	MISS	MISS	WT	WT	WT	MISS
R175H		R175H		R175H	P278S	G266E				E285K
ERpHER2n	ERpHER2n	ERnHER2n	ERpHER2n	ERpHER2n	ERnHER2n	ERpHER2n	ERpHER2n	ERpHER2n	ERpHER2n	ERpHER2n
Died of Disease	Died of Disease	Living	Died of Disease	Died of Disease	Died of Disease	Died of Disease	Died of Disease	Living	Died of Other Causes	Living
140.7666667	59.5	114.4666667	122	106.9666667	36.63333333	42.96666667	61.96666667	144.7666667	58.76666667	87.83333333
YES	YES	YES	NO	NO	YES	NO	NO	YES	YES	YES

MB_0631	MB_0363	MB_0427	MB_0519	MB_0371	MB_0380	MB_0221	MB_0348	MB_0261	MB_0576	MB_0385
WT	WT	WT	MUT	WT	MUT	MUT	WT	WT	WT	WT
WT	WT	WT	NULL	WT	MISS	MISS	WT	WT	WT	WT
			X331_splice		N239D	V197G				
ERpHER2n	ERpHER2n	ERpHER2n	ERpHER2n	HER2p	ERpHER2n	ERnHER2n	ERpHER2n	ERpHER2n	ERpHER2n	ERpHER2n
Died of Disease	Died of Disease	Living	Living	Living	Died of Other Causes	Died of Disease	Died of Other Causes	Died of Disease	Died of Disease	Died of Disease
47.43333333	89.9	116.1	110.9666667	131.1333333	69.33333333	20.2	122.7	90.23333333	31	36.43333333
YES	NO	NO	YES	NO	NO	NO	NO	NO	NO	YES

MB_0659	MB_0270	MB_0379	MB_0527	MB_0624	MB_0273	MB_0050	MB_0460	MB_0654	MB_0454	MB_0392
WT	WT	WT	WT	WT	WT	WT	WT	WT	WT	WT
WT	WT	WT	WT	WT	WT	WT	WT	WT	WT	WT
ERnHER2n	ERpHER2n	ERpHER2n	ERpHER2n	ERpHER2n	ERpHER2n	ERpHER2n	ERpHER2n	ERpHER2n	ERpHER2n	ERpHER2n
Died of Disease	Living	Living	Died of Other Causes	Living	Living	Living	Died of Disease	Living	Died of Other Causes	Died of Disease
23.33333333	337.0333333	135.6666667	109.8333333	64.03333333	186.5333333	75.33333333	114	69.4	46.83333333	38.13333333
NO	NO	YES	NO	NO	NO	YES	NO	NO	NO	YES

MB_0336	MB_0467	MB_0349	MB_0378	MB_0176	MB_0429	MB_0397	MB_0571	MB_0426	MB_0135	MB_0112
WT	MUT	WT	MUT	WT	WT	WT	WT	WT	WT	WT
WT	NULL	WT	NULL	WT	WT	WT	WT	WT	WT	WT
	S90Pfs*33		S20Qfs*24							
ERpHER2n	HER2p	ERpHER2n	HER2p	ERpHER2n	ERpHER2n	ERpHER2n	ERpHER2n	ERpHER2n	ERpHER2n	ERpHER2n
Died of Disease	Died of Disease	Died of Other Causes	Died of Disease	Living	Died of Other Causes	Living	Living	Living	Living	Died of Disease
170.3	105.6	146.0333333	97.3	113.4333333	74.46666667	57.23333333	149.8666667	131.1	116.6333333	39.16666667
NO	YES	NO	YES	YES	NO	NO	NO	YES	NO	NO

MB_0352	MB_0644	MB_0601	MB_0568	MB_0328	MB_0325	MB_0358	MB_0413	MB_0158	MB_0636	MB_0145	MB_0195
WT	WT	MUT	WT	WT	MUT	MUT	WT	MUT	MUT	WT	WT
WT	WT	MISS	WT	WT	NULL	NULL	WT	NULL	MISS	WT	WT
		Y126C			R213Dfs*34	X225_splice		p.?	R273H		
ERnHER2n	ERpHER2n	ERpHER2n	ERpHER2n	ERpHER2n	ERpHER2n	ERpHER2n	ERpHER2n	ERnHER2n	ERpHER2n	ERpHER2n	ERpHER2n
Died of Disease	Living	Died of Disease	Living	Died of Disease	Living	Living	Living	Living	Died of Disease	Living	Living
55.2	93.3	29.66666667	181.4666667	125.6	177.5333333	32.86666667	140.0666667	192.3	32.63333333	147.6666667	146
YES	NO	YES	NO	NO	NO	YES	YES	YES	NO	NO	NO

MB_0422	MB_0483	MB_0317	MB_0486	MB_0139	MB_0257	MB_0345	MB_0375	MB_0419	MB_0480
WT	WT	WT	WT	WT	WT	WT	MUT	WT	MUT
WT	WT	WT	WT	WT	WT	WT	NULL	WT	NULL
							A74Sfs*71		F338Rfs*8
ERpHER2n	ERpHER2n	ERpHER2n	ERpHER2n	ERpHER2n	ERpHER2n	ERpHER2n	ERpHER2n	ERpHER2n	ERpHER2n
Died of Other Causes	Died of Disease	Died of Other Causes	Living	Living	Died of Disease	Living	Living	Died of Other Causes	Living
33.8	74.46666667	151.6666667	89.96666667	109.2	91.73333333	72.66666667	143.1666667	104.3	94.56666667
NO	YES	NO	YES	NO	YES	YES	NO	NO	NO

MB_0311	MB_0324	MB_0368	MB_0389	MB_0248	MB_0035	MB_0667	MB_0423	MB_0904	MB_0119	MB_0650
WT	WT	WT	WT	WT	WT	WT	WT	WT	WT	WT
WT	WT	WT	WT	WT	WT	WT	WT	WT	WT	WT
ERpHER2n	ERpHER2n	ERpHER2n	HER2p	ERpHER2n	ERpHER2n	HER2p	ERpHER2n	ERpHER2n	ERpHER2n	ERpHER2n
Died of Other Causes	Died of Disease	Died of Disease	Living	Living	Died of Disease	Died of Disease	Living	Died of Other Causes	Died of Disease	Living
151.1666667	144.9666667	61.9	96.96666667	71	36.26666667	128	138.9333333	144.7	95.86666667	25.53333333
NO	YES	YES	YES	YES	NO	NO	NO	NO	NO	NO

MB_0204	MB_0184	MB_0600	MB_0400	MB_0511	MB_0059	MB_0500	MB_0150	MB_0895	MB_0366	MB_0173	MB_0131	MB_0206
WT	WT	WT	MUT	WT	WT	MUT	MUT	MUT	WT	WT	WT	MUT
WT	WT	WT	MISS	WT	WT	NULL	MISS	MISS	WT	WT	WT	MISS
			R273C			R110Pfs*39	K132N	G244S				C135W
ERpHER2n	ERpHER2n	ERpHER2n	ERnHER2n	ERpHER2n	ERpHER2n	ERnHER2n	ERpHER2n	HER2p	ERpHER2n	ERpHER2n	ERpHER2n	ERnHER2n
Died of Disease	Living	Living	Died of Disease	Living	Living	Living	Living	Died of Other Causes	Living	Living	Died of Disease	Living
24.3	109.0333333	111	22.46666667	61.7	160.9	67.46666667	111.2	43.1	64.23333333	3.766666667	66.63333333	141.1666667
NO	NO	NO	YES	NO	NO	YES	NO	NO	NO	NO	YES	YES

MB_0315	MB_0361	MB_0545	MB_0370	MB_0642	MB_0431	MB_0181	MB_0603	MB_0295	MB_0618	MB_0496
MUT	MUT	MUT	WT	WT	WT	WT	WT	WT	WT	WT
NULL	MISS	MISS	WT	WT	WT	WT	WT	WT	WT	WT
P191Lfs*56	R248Q	C242S								
ERpHER2n	HER2p	ERpHER2n	ERpHER2n	ERpHER2n	ERpHER2n	ERpHER2n	ERpHER2n	ERpHER2n	ERpHER2n	ERpHER2n
Living	Died of Other Causes	Died of Other Causes	Died of Other Causes	Died of Other Causes	Living	Living	Living	Living	Living	Living
186.2	15.06666667	41.16666667	93.5	84.2	85.13333333	85.96666667	121.9666667	164.5	101.9	130.9
YES	NO	NO	YES	NO	NO	NO	NO	NO	NO	YES

MB_0411	MB_0285	MB_0360	MB_0359	MB_0344	MB_0583	MB_0202	MB_0485	MB_0609	MB_0538	MB_0197	MB_0410	MB_0528
MUT	WT	WT	WT	MUT	WT	MUT	MUT	MUT	WT	WT	WT	MUT
NULL	WT	WT	WT	MISS	WT	MISS	MISS	MISS	WT	WT	WT	NULL
G245del				E285K		D281H	C238R	R249S				Q104*
ERpHER2n	ERpHER2n	ERpHER2n	ERpHER2n	ERpHER2n	ERpHER2n	ERpHER2n	ERpHER2n	ERpHER2n	ERpHER2n	ERpHER2n	ERpHER2n	ERpHER2n
Living	Living	Died of Disease	Living	Living	Living	Living	Died of Disease	Died of Disease	Died of Disease	Living	Living	Living
139.1666667	3.366666667	132.5666667	21.6	152.9333333	62.63333333	128.3666667	36.76666667	98.7	24.1	70.73333333	63	193.9666667
YES	NO	NO	NO	NO	NO	YES	YES	YES	YES	YES	NO	YES

MB_0165	MB_0152	MB_0148	MB_0594	MB_0521	MB_0532	MB_0536	MB_0319	MB_0491	MB_0404	MB_0243	MB_0580
MUT	MUT	MUT	WT	WT	WT	MUT	WT	WT	WT	WT	MUT
MISS	NULL	NULL	WT	WT	WT	MISS	WT	WT	WT	WT	NULL
H214R	G266Rfs*74	Q192*				H179R					H214Lfs*33
HER2p	HER2p	HER2p	ERpHER2n	ERpHER2n	ERpHER2n	ERpHER2n	ERpHER2n	ERpHER2n	ERpHER2n	ERpHER2n	ERpHER2n
Died of Disease	Living	Living	Living	Living	Died of Disease	Died of Other Causes	Died of Disease	Living	Living	Living	Died of Disease
47.63333333	63.03333333	1.766666667	44.23333333	212.2	143.1333333	147.3666667	40.7	125.8	63.5	149.7666667	79.36666667
YES	NO	NO	NO	NO	YES	NO	NO	NO	NO	YES	YES

MB_0312	MB_0635	MB_0649	MB_0643	MB_0581	MB_0383	MB_0142	MB_0320	MB_0207	MB_0167	MB_0591	MB_0613
WT	WT	WT	MUT	MUT	WT	WT	WT	WT	MUT	WT	MUT
WT	WT	WT	NULL	MISS	WT	WT	WT	WT	MISS	WT	NULL
			T304Nfs*42	L194R					R282W		E287*
ERpHER2n	ERnHER2n	ERpHER2n	HER2p	ERnHER2n	ERpHER2n	ERpHER2n	ERpHER2n	ERpHER2n	ERpHER2n	ERpHER2n	ERnHER2n
Died of Disease	Living	Living	Died of Other Causes	Died of Disease	Died of Disease	Living	Living	Living	Died of Disease	Died of Disease	Living
169	88	61.43333333	19.73333333	7.866666667	85.73333333	12.4	63.73333333	173.6333333	43.1	117.7666667	48.8
NO	NO	NO	NO	NO	NO	NO	NO	NO	NO	NO	YES

MB_0313	MB_0599	MB_0286	MB_0575	MB_0549	MB_0214	MB_0589	MB_0406	MB_0452	MB_0524	MB_0880	MB_0573	MB_0664
MUT	WT	WT	WT	WT	MUT	WT	WT	MUT	MUT	WT	WT	MUT
MISS	WT	WT	WT	WT	MISS	WT	WT	MISS	MISS	WT	WT	NULL
Y220C					V157F			R248Q	C275Y			E271Gfs*74
ERpHER2n	ERpHER2n	ERpHER2n	ERpHER2n	HER2p	ERnHER2n	HER2p	ERpHER2n	ERpHER2n	ERpHER2n	ERpHER2n	ERpHER2n	ERnHER2n
Living	Living	Living	Living	Living	Living	Living	Died of Disease	Living	Living	Living	Died of Other Causes	Died of Disease
184.7666667	123.2666667	192.6	128.5666667	25.96666667	146.9	125.8	96.9	62.8	80.83333333	1.233333333	163.2	11.06666667
NO	YES	NO	YES	NO	YES	NO	YES	NO	YES	NO	NO	YES

MB_0446	MB_0008	MB_0656	MB_0154	MB_0597	MB_0550	MB_0616	MB_0448	MB_0412	MB_0122	MB_0425
MUT	MUT	MUT	WT	WT	WT	WT	WT	MUT	WT	WT
MISS	MISS	MISS	WT	WT	WT	WT	WT	NULL	WT	WT
C141R	S241F	V216L						T102Pfs*21		
ERnHER2n	ERpHER2n	HER2p	ERpHER2n	ERpHER2n	ERpHER2n	ERpHER2n	ERpHER2n	ERpHER2n	ERpHER2n	ERpHER2n
Living	Died of Disease	Died of Disease	Living	Died of Other Causes	Died of Disease	Died of Other Causes	Died of Disease	Living	Living	Died of Other Causes
72.26666667	41.36666667	19.03333333	114.7666667	113.0666667	132.3333333	29.03333333	29.23333333	136.1666667	138.9	24.4
YES	YES	YES	NO	YES	NO	NO	YES	NO	NO	NO

MB_0314	MB_0356	MB_0440	MB_0398	MB_0438	MB_0449	MB_0162	MB_0593	MB_0301	MB_0628	MB_0283	MB_0106
MUT	WT	WT	MUT	WT	WT	WT	WT	WT	WT	WT	WT
NULL	WT	WT	NULL	WT	WT	WT	WT	WT	WT	WT	WT
X224_splice			D148Hfs*31								
HER2p	ERpHER2n	ERpHER2n	ERpHER2n	HER2p	ERpHER2n	ERpHER2n	HER2p	ERpHER2n	ERpHER2n	ERpHER2n	ERpHER2n
Died of Disease	Living	Living	Died of Disease	Living	Living	Living	Died of Disease	Died of Other Causes	Living	Died of Disease	Living
4.433333333	213.5	100.8333333	43.2	135.1666667	112.5666667	55.76666667	34.66666667	122.7	105.9666667	22.03333333	85.33333333
NO	NO	YES	NO	YES	YES	NO	YES	NO	NO	NO	YES

MB_0341	MB_0474	MB_0394	MB_0373	MB_0247	MB_0225	MB_0424	MB_0209	MB_0115	MB_0136	MB_0542	MB_0354
WT	MUT	WT	MUT	WT	WT	WT	WT	MUT	WT	WT	MUT
WT	MISS	WT	MISS	WT	WT	WT	WT	MISS	WT	WT	MISS
	G245V		Y234H					R175H			H178P
ERpHER2n	ERpHER2n	ERpHER2n	HER2p	ERpHER2n	HER2p	ERnHER2n	ERpHER2n	ERnHER2n	ERpHER2n	ERpHER2n	ERnHER2n
Died of Disease	Died of Disease	Living	Died of Disease	Living	Living	Died of Disease	Living	Died of Disease	Living	Died of Disease	Died of Disease
170.0333333	111.1	64.23333333	27	185.6	212.2	9.833333333	82.36666667	66.73333333	88.2	48.6	11.6
NO	YES	NO	NO	NO	NO	YES	NO	YES	NO	YES	NO

MB_0151	MB_0608	MB_0657	MB_0559	MB_0893	MB_0514	MB_0395	MB_0294	MB_0439	MB_0481	MB_0529	MB_0224
WT	MUT	WT	WT	WT	WT	MUT	WT	WT	MUT	WT	WT
WT	MISS	WT	WT	WT	WT	NULL	WT	WT	NULL	WT	WT
	R175H					H115Afs*34			Y107*		
ERpHER2n	ERnHER2n	ERpHER2n	ERpHER2n	ERnHER2n	ERpHER2n	HER2p	ERnHER2n	ERpHER2n	ERnHER2n	ERpHER2n	ERpHER2n
Died of Disease	Died of Other Causes	Living	Living	Living	Living	Died of Disease	Living	Living	Died of Disease	Died of Disease	Living
49.23333333	63.96666667	30.43333333	161.7666667	175.6333333	13.4	29	72.9	112.1333333	36.4	48.53333333	176.7666667
NO	NO	NO	YES	YES	YES	NO	YES	NO	YES	YES	NO

MB_0302	MB_0126	MB_0220	MB_0192	MB_0121	MB_0239	MB_0364	MB_0232	MB_0884	MB_0238	MB_0194	MB_0882
WT	WT	WT	WT	WT	WT	WT	WT	MUT	MUT	WT	MUT
WT	WT	WT	WT	WT	WT	WT	WT	NULL	MISS	WT	NULL
								V218Hfs*5	R248Q		R213*
ERpHER2n	ERpHER2n	ERnHER2n	ERpHER2n	ERpHER2n	ERpHER2n	ERpHER2n	ERpHER2n	ERpHER2n	ERnHER2n	ERpHER2n	ERpHER2n
Died of Disease	Living	Died of Disease	Living	Living	Died of Disease	Living	Living	Died of Disease	Living	Living	Died of Disease
84.2	127.6333333	39.83333333	144.6333333	152.2	85.56666667	176.6	207.6333333	93.66666667	193.1666667	91.6	42.36666667
NO	NO	NO	NO	NO	NO	YES	NO	YES	YES	YES	NO

MB_0010	MB_0236	MB_0377	MB_0123	MB_0504	MB_0564	MB_0475	MB_0321	MB_0482	MB_0101	MB_0662	MB_0291
MUT	MUT	WT	WT	WT	WT	WT	WT	MUT	WT	MUT	WT
NULL	MISS	WT	WT	WT	WT	WT	WT	MISS	WT	MISS	WT
P67Qfs*56	N239D							R337C		D281G	
ERpHER2n	HER2p	ERpHER2n	ERpHER2n	ERpHER2n	HER2p	ERpHER2n	ERpHER2n	HER2p	ERpHER2n	HER2p	HER2p
Died of Disease	Living	Living	Died of Other Causes	Living	Died of Disease	Living	Living	Died of Disease	Living	Died of Disease	Died of Disease
7.8	205.0333333	134.3666667	114.2333333	130.4666667	37.93333333	130.7	174.6333333	24.86666667	148.0333333	69.8	39.2
NO	YES	NO	NO	NO	YES	NO	YES	YES	NO	NO	YES

MB_0465	MB_0630	MB_0036	MB_0166	MB_0525	MB_0307	MB_0002	MB_0408	MB_0466	MB_0632	MB_0120	MB_0507	MB_0287
MUT	WT	WT	WT	MUT	WT	MUT	WT	WT	WT	WT	WT	WT
MISS	WT	WT	WT	MISS	WT	MISS	WT	WT	WT	WT	WT	WT
R337C				R110P		H178P						
HER2p	HER2p	ERpHER2n	ERpHER2n	ERnHER2n	HER2p	ERpHER2n	ERpHER2n	ERpHER2n	ERpHER2n	HER2p	ERpHER2n	ERpHER2n
Died of Disease	Living	Died of Disease	Living	Living	Living	Living	Living	Living	Living	Died of Disease	Living	Died of Other Causes
28.6	86.8	132.0333333	104.4	65.86666667	177.3333333	84.63333333	101.8333333	43.86666667	56.5	29.06666667	23.8	94.73333333
NO	YES	NO	YES	YES	YES	NO	YES	YES	NO	NO	NO	NO

MB_0869	MB_4633	MB_4627	MB_4004	MB_4708	MB_4618	MB_4641	MB_4622	MB_4634	MB_4688
MUT	WT	WT	WT	WT	WT	WT	MUT	WT	WT
NULL	WT	WT	WT	WT	WT	WT	MISS	WT	WT
R213*							R248Q		
ERnHER2n	ERpHER2n	ERpHER2n	ERpHER2n	ERpHER2n	ERpHER2n	ERpHER2n	ERnHER2n	ERpHER2n	HER2p
Died of Other Causes	Living	Died of Other Causes	Died of Disease	Died of Other Causes	Living	Died of Other Causes	Died of Other Causes	Died of Disease	Died of Disease
112.4	318.2	186.6333333	256.8666667	227.9	267.4	148.5666667	282.8333333	109.6	85.3
YES	NO	NO	NO	NO	NO	NO	YES	NO	NO

MB_4665	MB_4666	MB_4640	MB_4691	MB_4671	MB_4667	MB_4721	MB_4723	MB_4718	MB_4716	MB_4729
WT	MUT	MUT	MUT	WT	MUT	WT	WT	WT	WT	MUT
WT	MISS	MISS	NULL	WT	MISS	WT	WT	WT	WT	MISS
	R248W	I195T	P128Lfs*42		E271K					R158L
ERpHER2n	ERpHER2n	ERnHER2n	ERpHER2n	ERpHER2n	ERnHER2n	ERpHER2n	ERpHER2n	ERpHER2n	ERpHER2n	HER2p
Living	Died of Disease	Living	Died of Disease	Died of Disease	Died of Other Causes	Died of Disease	Living	Died of Disease	Died of Disease	Died of Other Causes
260.2	111.5666667	200.6	282.5666667	34.7	295.3333333	23.73333333	211.9666667	198.3333333	76.13333333	28
NO	NO	NO	NO	NO	NO	NO	NO	NO	NO	NO

MB_4717	MB_4724	MB_4770	MB_4752	MB_4762	MB_5169	MB_4796	MB_4790	MB_4797	MB_4800	MB_5541
MUT	MUT	MUT	MUT	WT	WT	WT	WT	WT	WT	WT
NULL	NULL	MISS	NULL	WT	WT	WT	WT	WT	WT	WT
C124*	I50Mfs*73	R248W	X307_splice							
ERnHER2n	HER2p	HER2p	ERpHER2n	ERpHER2n	ERpHER2n	HER2p	ERpHER2n	ERpHER2n	ERpHER2n	ERpHER2n
Died of Disease	Living	Died of Other Causes	Died of Disease	Living	Died of Disease	Living	Died of Disease	Died of Other Causes	Died of Disease	Living
42.9	146.0666667	152.0666667	125.2666667	252.1	51.96666667	128.2666667	70.46666667	99.36666667	110.9333333	200.1333333
NO	NO	NO	NO	NO	NO	NO	NO	NO	NO	NO

MB_5549	MB_5519	MB_5495	MB_4832	MB_4745	MB_4825	MB_4814	MB_4757	MB_4694	MB_4698	MB_4715
MUT	WT	WT	WT	MUT	WT	MUT	MUT	MUT	WT	MUT
MISS	WT	WT	WT	MISS	WT	MISS	NULL	NULL	WT	MISS
D281H				R248W		R273P	E349*	X125_splice		R273C
HER2p	ERpHER2n	ERpHER2n	ERpHER2n	HER2p	ERpHER2n	ERpHER2n	ERnHER2n	ERpHER2n	ERpHER2n	ERnHER2n
Died of Disease	Died of Other Causes	Living	Died of Disease	Died of Disease	Died of Other Causes	Died of Disease	Died of Disease	Living	Died of Disease	Died of Disease
88.93333333	169.2333333	228.8	141.7333333	42.66666667	214.4333333	73.46666667	27.4	198.3	170.6666667	35.23333333
YES	NO	NO	NO	YES	NO	NO	YES	NO	NO	YES

MB_4685	MB_4712	MB_4672	MB_4704	MB_4655	MB_4651	MB_4661	MB_4649	MB_4642
WT	MUT	WT	WT	MUT	MUT	WT	MUT	WT
WT	MISS	WT	WT	MISS	NULL	WT	NULL	WT
	C176Y			G244C	H193Qfs*53		Q331*	
ERpHER2n	ERpHER2n	ERpHER2n	ERpHER2n	ERpHER2n	ERpHER2n	ERpHER2n	ERpHER2n	ERpHER2n
Died of Other Causes	Died of Other Causes	Died of Other Causes	Died of Disease	Died of Other Causes	Died of Disease	Living	Died of Other Causes	Died of Disease
43.3	249.5333333	220.9333333	64	99	30.3	234.2333333	16.83333333	285.7
NO	NO	NO	NO	NO	NO	NO	NO	NO

MB_4674	MB_4648	MB_5197	MB_5152	MB_5201	MB_5189	MB_5281	MB_5212	MB_5172
WT	WT	WT	WT	WT	MUT	WT	WT	WT
WT	WT	WT	WT	WT	MISS<br>P278R	WT	WT	WT
ERpHER2n	ERpHER2n	ERpHER2n	ERpHER2n	ERpHER2n	ERpHER2n	ERnHER2n	HER2p	HER2p
Died of Disease	Died of Other Causes	Died of Disease	Died of Other Causes	Died of Other Causes	Died of Disease	Living	Died of Other Causes	Living
81.06666667	168.5333333	223.8333333	115.9333333	96.96666667	111.9666667	79.3	179.1	184.2666667
NO	NO	NO	NO	NO	NO	NO	NO	YES

MB_5183	MB_5185	MB_5022	MB_5014	MB_4994	MB_5017	MB_4982	MB_5004	MB_4986	MB_5327	MB_5341
WT	WT	WT	WT	WT	MUT	MUT	MUT	WT	MUT	WT
WT	WT	WT	WT	WT	MISS	NULL	NULL	WT	NULL	WT
					G262V	S166Hfs*4	I255del		C141*	
ERpHER2n	ERpHER2n	HER2p	ERpHER2n	ERpHER2n	ERpHER2n	ERnHER2n	ERpHER2n	ERpHER2n	HER2p	ERpHER2n
Died of Other Causes	Died of Disease	Died of Disease	Living	Died of Other Causes	Living	Died of Disease	Living	Died of Disease	Living	Living
118.5333333	77.46666667	118.6	213.3666667	174.1333333	144.9333333	19.16666667	216.7333333	79.8	163.5333333	250.8333333
NO	NO	NO	NO	NO	NO	NO	YES	NO	NO	NO

MB_5328	MB_5318	MB_5287	MB_5324	MB_4682	MB_4710	MB_4701	MB_4709	MB_4686	MB_4706
WT	MUT	WT	WT	WT	WT	WT	WT	WT	WT
WT	NULL	WT	WT	WT	WT	WT	WT	WT	WT
	C229Yfs*10								
ERpHER2n	ERpHER2n	ERpHER2n	ERpHER2n	ERpHER2n	ERpHER2n	ERpHER2n	ERpHER2n	ERpHER2n	ERpHER2n
Died of Other Causes	Died of Disease	Died of Other Causes	Died of Other Causes	Died of Disease	Living	Living	Living	Died of Disease	Died of Other Causes
14.2	14.16666667	124	190.1	139.5333333	57.66666667	127.5333333	146.7666667	48.43333333	55.66666667
NO	NO	NO	NO	NO	NO	NO	NO	NO	NO

MB_4719	MB_4702	MB_4908	MB_4871	MB_4906	MB_4911	MB_4866	MB_4858	MB_4862	MB_4872	MB_4887
MUT	WT	MUT	WT	WT	MUT	WT	MUT	MUT	WT	MUT
MISS	WT	NULL	WT	WT	MISS	WT	NULL	NULL	WT	NULL
E11Q		Q317*			C238Y		R342*	P390Lfs*32		I255Nfs*9
ERpHER2n	ERpHER2n	HER2p	HER2p	ERpHER2n	ERnHER2n	ERpHER2n	HER2p	ERpHER2n	ERpHER2n	ERpHER2n
Died of Other Causes	Living	Died of Disease	Died of Disease	Died of Other Causes	Died of Disease	Living	Died of Other Causes	Living	Living	Died of Disease
81.56666667	300.8666667	47.9	39.8	233.8666667	31.43333333	224.3	129.3333333	187.3	157.1	35.4
NO	NO	NO	NO	NO	YES	NO	NO	NO	NO	NO

MB_4867	MB_4888	MB_4929	MB_4945	MB_4930	MB_4894	MB_4898	MB_4670	MB_5013	MB_4977	MB_4967
WT	MUT	MUT	MUT	MUT	MUT	WT	WT	WT	WT	WT
WT	NULL	MISS	MISS	MISS	MISS	WT	WT	WT	WT	WT
	E258*	E285K	R273C	F134C	H179Y					
ERpHER2n	ERnHER2n	HER2p	ERnHER2n	HER2p	ERpHER2n	ERpHER2n	ERpHER2n	ERpHER2n	ERpHER2n	ERpHER2n
Living	Living	Died of Disease	Died of Disease	Living	Living	Died of Disease	Died of Disease	Died of Other Causes	Died of Disease	Died of Other Causes
221.9	230.5	40	20.13333333	58.43333333	232.4	117.9	67.8	251.2	216.7333333	130.7
NO	NO	NO	YES	NO	NO	NO	NO	NO	NO	NO

MB_4981	MB_4003	MB_4968	MB_5052	MB_5049	MB_5041	MB_5044	MB_5072	MB_4171	MB_5053	MB_5045
WT	MUT	WT	MUT	WT	MUT	WT	MUT	WT	WT	MUT
WT	NULL	WT	NULL	WT	MISS	WT	MISS	WT	WT	NULL
	M246*		S183*		R337C		N239I			A74Pfs*49
ERpHER2n	ERpHER2n	ERpHER2n	ERnHER2n	ERpHER2n	ERnHER2n	ERpHER2n	ERnHER2n	ERpHER2n	ERpHER2n	ERpHER2n
Died of Disease	Died of Disease	Living	Died of Other Causes	Living	Living	Living	Died of Disease	Died of Disease	Died of Other Causes	Died of Other Causes
180.7666667	106.5666667	78.7	34.56666667	255.3	173.9333333	139.4333333	50.23333333	184.7	150.6	168.3
NO	NO	NO	NO	NO	NO	NO	YES	NO	NO	NO

MB_5116	MB_5120	MB_5074	MB_5119	MB_5114	MB_4230	MB_4154	MB_4737	MB_4764	MB_4735
WT	MUT	WT	MUT	MUT	WT	MUT	WT	WT	WT
WT	NULL	WT	MISS	MISS	WT	NULL	WT	WT	WT
	P223Afs*2		H193R	R273L		V217Gfs*29			
ERpHER2n	HER2p	ERpHER2n	ERpHER2n	HER2p	ERpHER2n	ERpHER2n	ERpHER2n	ERpHER2n	ERpHER2n
Died of Disease	Died of Disease	Died of Other Causes	Living	Died of Disease	Died of Other Causes	Died of Other Causes	Living	Living	Living
71.76666667	27.8	221.6	59.76666667	15.36666667	115.6	49.56666667	122.2	245.5	297.8
NO	YES	NO	NO	NO	NO	NO	NO	NO	NO

MB_4730	MB_4733	MB_4758	MB_5033	MB_4741	MB_4732	MB_5305	MB_5256	MB_5273	MB_5236	MB_5238
WT	MUT	MUT	WT	WT	MUT	WT	WT	WT	MUT	WT
WT	MISS	MISS	WT	WT	MISS	WT	WT	WT	NULL	WT
	G154S	R248W			I195T				C242Afs*5	
ERpHER2n	ERnHER2n	ERnHER2n	ERpHER2n	ERpHER2n	ERnHER2n	ERpHER2n	ERpHER2n	ERpHER2n	ERnHER2n	HER2p
Died of Other Causes	Died of Disease	Living	Died of Disease	Died of Disease	Living	Died of Disease	Died of Disease	Died of Disease	Living	Died of Disease
131.3	19.73333333	211.9333333	52.73333333	56.5	149.8666667	149.4333333	108.0666667	60.86666667	166.6666667	44.13333333
NO	YES	NO	NO	NO	YES	NO	NO	NO	YES	YES

MB_5233	MB_5244	MB_5253	MB_5260	MB_4998	MB_4993	MB_5001	MB_5084	MB_4969
WT	WT	WT	WT	WT	MUT	WT	WT	WT
WT	WT	WT	WT	WT	NULL	WT	WT	WT
					R174Sfs*67			
ERpHER2n	ERpHER2n	ERpHER2n	ERpHER2n	ERpHER2n	ERnHER2n	ERpHER2n	ERpHER2n	ERpHER2n
Died of Other Causes	Died of Disease	Died of Other Causes	Died of Other Causes	Died of Disease	Died of Disease	Died of Disease	Died of Disease	Died of Other Causes
209.0333333	216.8666667	102.0666667	202.1	65.56666667	75.23333333	81.8	198.1	198.1
NO	NO	NO	NO	NO	YES	NO	NO	NO

MB_4999	MB_5011	MB_4959	MB_4599	MB_4616	MB_4623	MB_4644	MB_4869	MB_4878	MB_4851
WT	WT	WT	WT	WT	MUT	MUT	WT	WT	WT
WT	WT	WT	WT	WT	MISS	NULL	WT	WT	WT
					A276P	*394T			
ERpHER2n	ERpHER2n	ERpHER2n	ERpHER2n	ERpHER2n	ERpHER2n	HER2p	ERpHER2n	HER2p	ERpHER2n
Living	Died of Other Causes	Died of Disease	Living	Died of Disease	Died of Other Causes	Died of Disease	Died of Disease	Died of Disease	Died of Disease
187.9333333	80.5	117.5666667	191.1666667	90.13333333	292.0333333	90	29.3	22.46666667	90.26666667
NO	NO	NO	NO	NO	NO	NO	NO	NO	NO

MB_4233	MB_4937	MB_4934	MB_4899	MB_4912	MB_4935	MB_4933	MB_4900	MB_4941	MB_5221	MB_5139
MUT	WT	WT	WT	WT	WT	WT	MUT	WT	WT	WT
NULL	WT	WT	WT	WT	WT	WT	MISS	WT	WT	WT
N268Lfs*75							A159V			
ERpHER2n	ERpHER2n	ERpHER2n	ERpHER2n	ERpHER2n	HER2p	ERpHER2n	ERpHER2n	ERpHER2n	ERpHER2n	ERpHER2n
Died of Disease	Died of Disease	Living	Living	Died of Disease	Living	Living	Living	Died of Disease	Died of Other Causes	Died of Other Causes
36.36666667	77.23333333	70.23333333	275.7333333	50	194.2	272.9	224.6	58.63333333	131.3	89.96666667
NO	NO	NO	NO	NO	YES	NO	NO	NO	NO	NO

MB_5222	MB_5097	MB_5338	MB_5315	MB_5195	MB_5226	MB_5232	MB_5160	MB_5126	MB_5124
WT	MUT	MUT	MUT	MUT	WT	MUT	WT	MUT	WT
WT	MISS	MISS	MISS	MISS	WT	NULL	WT	MISS	WT
	R248W	S215G	N239D	K132E		S314*		M237I	
ERnHER2n	ERpHER2n	ERpHER2n	HER2p	HER2p	ERpHER2n	ERnHER2n	ERpHER2n	ERnHER2n	ERpHER2n
Died of Other Causes	Living	Died of Disease	Died of Disease	Died of Disease	Living	Died of Other Causes	Died of Disease	Died of Disease	Died of Disease
111.8333333	61.6	51.66666667	43.03333333	196.8666667	200.6	211.2	87.73333333	27.3	124.2
NO	NO	NO	YES	NO	NO	NO	NO	YES	NO

MB_4855	MB_5279	MB_4173	MB_5145	MB_4944	MB_4961	MB_5121	MB_4956	MB_4886
MUT	WT	WT	MUT	WT	WT	WT	WT	MUT
MISS	WT	WT	NULL	WT	WT	WT	WT	NULL
V272L			S185Tfs*62					S260Pfs*85
ERpHER2n	ERpHER2n	ERpHER2n	ERnHER2n	ERpHER2n	ERpHER2n	ERpHER2n	ERpHER2n	HER2p
Died of Other Causes	Died of Other Causes	Died of Other Causes	Died of Disease	Died of Other Causes	Living	Died of Disease	Died of Disease	Died of Disease
41.46666667	68.76666667	160.3333333	159.2333333	237.2666667	219.4666667	114.9	69.3	48.16666667
NO	NO	NO	NO	NO	NO	NO	NO	YES

MB_4950	MB_4965	MB_4962	MB_4952	MB_5267	MB_5266	MB_5396	MB_4938	MB_5351	MB_5347	MB_5312
WT	WT	WT	MUT	WT	MUT	MUT	WT	WT	WT	WT
WT	WT	WT	MISS	WT	MISS	NULL	WT	WT	WT	WT
			G266V		R248W	Q192*				
ERpHER2n	ERpHER2n	ERpHER2n	HER2p	ERpHER2n	HER2p	ERpHER2n	ERnHER2n	HER2p	ERpHER2n	HER2p
Died of Disease	Died of Other Causes	Died of Disease	Living	Died of Disease	Died of Other Causes	Living	Died of Disease	Living	Died of Disease	Died of Disease
150.6	263.6	197.8333333	184.4	71.06666667	83.63333333	165.6666667	70.6	202.1	211.9	37.9
NO	NO	NO	NO	NO	NO	NO	YES	NO	NO	YES

MB_5311	MB_5284	MB_4148	MB_5078	MB_5088	MB_5064	MB_5107	MB_5066	MB_5068	MB_5061	MB_5070	MB_5059
WT	WT	WT	MUT	WT	WT	WT	WT	WT	WT	WT	WT
WT	WT	WT	NULL	WT	WT	WT	WT	WT	WT	WT	WT
			X187_splice								
ERpHER2n	ERpHER2n	ERpHER2n	HER2p	ERpHER2n	ERpHER2n	ERpHER2n	ERpHER2n	ERpHER2n	ERpHER2n	ERnHER2n	ERpHER2n
Died of Disease	Died of Other Causes	Died of Disease	Died of Disease	Died of Other Causes	Living	Died of Disease	Living	Living	Living	Living	Living
57.66666667	65.33333333	63.03333333	87.1	199.3666667	91.5	82.63333333	94.7	182.5	39.43333333	213.9	257.6333333
NO	NO	NO	NO	NO	NO	NO	NO	NO	NO	YES	NO

MB_4266	MB_4276	MB_4771	MB_4739	MB_5331	MB_4743	MB_4785	MB_4778	MB_4763	MB_4779	MB_4849
WT	MUT	WT	WT	WT	WT	WT	WT	MUT	WT	WT
WT	NULL	WT	WT	WT	WT	WT	WT	MISS	WT	WT
	H178Pfs*2							R248Q		
ERpHER2n	HER2p	ERpHER2n	ERpHER2n	HER2p	ERpHER2n	ERpHER2n	ERpHER2n	HER2p	ERpHER2n	ERpHER2n
Died of Disease	Died of Disease	Living	Died of Other Causes	Died of Disease	Living	Died of Disease	Died of Other Causes	Living	Living	Died of Disease
33.9	132.2	297.2333333	161.6666667	124.1333333	230.1666667	121.6666667	206.1333333	102.1	101.5666667	45.33333333
NO	NO	NO	NO	NO	NO	NO	NO	YES	NO	NO

MB_4787	MB_4767	MB_4784	MB_5043	MB_4992	MB_5020	MB_5019	MB_5015	MB_5048	MB_5035
WT	WT	WT	WT	WT	WT	MUT	WT	WT	WT
WT	WT	WT	WT	WT	WT	NULL	WT	WT	WT
						S183*			
ERpHER2n	ERpHER2n	ERpHER2n	ERpHER2n	ERnHER2n	ERpHER2n	ERnHER2n	ERpHER2n	ERpHER2n	ERpHER2n
Died of Other Causes	Died of Disease	Died of Other Causes	Died of Disease	Living	Died of Disease	Died of Disease	Died of Other Causes	Died of Disease	Died of Disease
81.06666667	47.13333333	145.7333333	128.3666667	165.3666667	84.83333333	37.8	252.3	86.36666667	213.2
NO	NO	NO	NO	NO	NO	NO	NO	NO	NO

MB_5398	MB_5377	MB_5291	MB_5403	MB_5224	MB_5369	MB_5404	MB_5397	MB_5378	MB_5388	MB_5395
WT	WT	WT	WT	WT	WT	WT	MUT	MUT	WT	WT
WT	WT	WT	WT	WT	WT	WT	MISS	MISS	WT	WT
							Y163C	R248Q		
ERpHER2n	ERpHER2n	ERpHER2n	ERpHER2n	ERpHER2n	ERpHER2n	ERpHER2n	ERpHER2n	ERpHER2n	ERpHER2n	ERpHER2n
Living	Died of Disease	Living	Died of Other Causes	Died of Disease	Died of Disease	Died of Disease	Died of Disease	Died of Disease	Died of Other Causes	Living
213	102	153.5333333	95.73333333	99.4	39.3	74.73333333	172.8666667	12.26666667	146.9333333	165.1666667
NO	YES	NO	NO	NO	NO	NO	NO	YES	NO	NO

MB_5365	MB_5389	MB_5393	MB_5361	MB_5392	MB_5147	MB_4140	MB_5138	MB_5123	MB_5065
WT	WT	WT	MUT	MUT	WT	WT	MUT	WT	MUT
WT	WT	WT	MISS	MISS	WT	WT	MISS	WT	MISS
			L194R	M246V			R273H		C275F
ERpHER2n	ERpHER2n	ERpHER2n	ERpHER2n	ERnHER2n	HER2p	ERpHER2n	ERnHER2n	ERpHER2n	ERnHER2n
Died of Disease	Died of Other Causes	Died of Other Causes	Died of Disease	Living	Living	Died of Other Causes	Died of Disease	Died of Disease	Living
23.03333333	124.7666667	154	15.36666667	237.2666667	20.26666667	114.5	37	90.3	184.8
NO	NO	NO	NO	NO	NO	NO	YES	NO	NO

MB_4127	MB_5058	MB_5050	MB_5027	MB_5060	MB_5062	MB_5383	MB_5101	MB_5258	MB_5268
MUT	MUT	WT	WT	WT	WT	WT	MUT	MUT	WT
NULL	MISS	WT	WT	WT	WT	WT	MISS	MISS	WT
H214_E221del	L194P						F134V	M246R	
HER2p	ERnHER2n	ERpHER2n	ERpHER2n	ERpHER2n	HER2p	ERpHER2n	ERpHER2n	ERnHER2n	ERpHER2n
Died of Other Causes	Died of Disease	Living	Living	Died of Other Causes	Living	Died of Disease	Died of Disease	Died of Other Causes	Living
258.1666667	178.7333333	184.3333333	189.8666667	150.1	168.2666667	113.6666667	34.33333333	101.0666667	186.1333333
YES	YES	NO	NO	NO	NO	NO	NO	NO	NO

MB_5261	MB_5272	MB_5264	MB_5259	MB_5306	MB_5270	MB_5411	MB_5490	MB_5421	MB_5348	MB_5429
WT	MUT	WT	MUT	WT	WT	MUT	WT	MUT	MUT	WT
WT	MISS	WT	MISS	WT	WT	MISS	WT	MISS	NULL	WT
	H193Y		R175H			H179L		C242Y	I195Sfs*52	
ERpHER2n	ERpHER2n	ERpHER2n	HER2p	ERpHER2n	ERpHER2n	HER2p	ERpHER2n	ERnHER2n	ERnHER2n	ERpHER2n
Died of Other Causes	Died of Disease	Living	Died of Disease	Living	Died of Other Causes	Died of Disease	Died of Disease	Living	Living	Died of Other Causes
116.4666667	201.9333333	199.3	46.16666667	159.7333333	187.8333333	26	51.2	194.5666667	169.6666667	30.7
NO	NO	NO	YES	NO	NO	YES	NO	NO	YES	NO

MB_5405	MB_5414	MB_5489	MB_5417	MB_5481	MB_5409	MB_5299	MB_5408	MB_5475	MB_5505	MB_5100
WT	MUT	WT	MUT	MUT	MUT	MUT	WT	WT	WT	MUT
WT	MISS	WT	NULL	NULL	MISS	MISS	WT	WT	WT	NULL
	H193L		F54Kfs*67	E298*	K132E	C176S				R306*
ERpHER2n	HER2p	ERpHER2n	HER2p	ERpHER2n	HER2p	ERnHER2n	ERnHER2n	ERpHER2n	ERpHER2n	ERnHER2n
Living	Died of Other Causes	Living	Living	Died of Disease	Living	Died of Disease	Living	Died of Other Causes	Died of Disease	Died of Disease
117.6	140.6	216.9666667	107.0666667	46.36666667	198.1333333	16.7	101.4	2.533333333	164.6	19.9
NO	NO	NO	YES	NO	YES	YES	NO	NO	NO	YES

MB_5502	MB_5485	MB_4293	MB_4289	MB_5293	MB_5381	MB_4834	MB_5498	MB_4749	MB_4818
MUT	WT	WT	WT	WT	MUT	WT	MUT	WT	WT
MISS	WT	WT	WT	WT	NULL	WT	NULL	WT	WT
A159D					E198*		V197Gfs*50		
ERpHER2n	ERpHER2n	ERpHER2n	HER2p	ERpHER2n	HER2p	ERpHER2n	ERpHER2n	ERpHER2n	ERpHER2n
Living	Died of Other Causes	Died of Other Causes	Died of Disease	Died of Disease	Died of Disease	Died of Disease	Living	Died of Other Causes	Died of Other Causes
189.7333333	31.8	219.1666667	96.83333333	208.9666667	201.8333333	36.16666667	225.1666667	51.46666667	76.23333333
NO	NO	NO	NO	NO	YES	NO	NO	NO	NO

MB_4805	MB_4809	MB_5418	MB_5441	MB_5424	MB_5296	MB_5446	MB_5093	MB_5350	MB_5230	MB_5206
WT	MUT	WT	MUT	WT	MUT	WT	WT	WT	WT	WT
WT	NULL	WT	MISS	WT	NULL	WT	WT	WT	WT	WT
	Q144*		C135F		R213*					
ERpHER2n	ERpHER2n	HER2p	HER2p	ERpHER2n	HER2p	ERnHER2n	ERpHER2n	ERpHER2n	ERpHER2n	ERpHER2n
Died of Disease	Living	Living	Died of Other Causes	Died of Disease	Died of Disease	Died of Disease	Died of Other Causes	Died of Disease	Living	Living
129.8	286.0666667	207.1666667	9.7	83.53333333	13.8	162.8333333	142.4333333	53.63333333	176.5	205.7333333
NO	NO	NO	NO	NO	NO	YES	NO	NO	NO	NO

MB_4687	MB_5433	MB_5442	MB_5427	MB_5491	MB_5493	MB_5451	MB_5454	MB_5360	MB_5229
MUT	WT	MUT	MUT	WT	MUT	WT	MUT	WT	MUT
NULL	WT	MISS	MISS	WT	NULL	WT	MISS	WT	MISS
P318Afs*19		R249G	K120E		G262Efs*9		R282W		L111Q
ERpHER2n	ERpHER2n	ERnHER2n	ERnHER2n	ERpHER2n	ERpHER2n	ERpHER2n	ERpHER2n	ERpHER2n	HER2p
Died of Other Causes	Died of Disease	Living	Died of Other Causes	Died of Disease	Died of Disease	Living	Died of Disease	Died of Other Causes	Died of Disease
74.1	176.3666667	205.8	155.7333333	65.83333333	57.23333333	189.9	50.66666667	161.1333333	33.96666667
NO	NO	YES	NO	NO	NO	NO	NO	NO	YES

MB_4827	MB_5455	MB_5370	MB_5290	MB_5366	MB_5382	MB_5402	MB_5288
WT	WT	WT	WT	WT	WT	WT	MUT
WT	WT	WT	WT	WT	WT	WT	MISS
							E285K
ERpHER2n	ERpHER2n	ERpHER2n	ERpHER2n	ERpHER2n	ERpHER2n	ERpHER2n	ERpHER2n
Died of Other Causes	Died of Other Causes	Died of Other Causes	Died of Other Causes	Living	Died of Other Causes	Died of Other Causes	Died of Other Causes
45.7	61.8	119.3	36.93333333	243.1666667	116.2333333	107.7666667	128.4
NO	NO	NO	NO	NO	NO	NO	NO

MB_5384	MB_5358	MB_4357	MB_4991	MB_5401	MB_5390	MB_5412	MB_5193	MB_5182	MB_5157	MB_5205
WT	WT	MUT	WT	WT	MUT	WT	WT	WT	MUT	MUT
WT	WT	MISS	WT	WT	NULL	WT	WT	WT	MISS	MISS
		K132N			L111Afs*11				V173L	K132E
ERpHER2n	ERpHER2n	ERpHER2n	ERpHER2n	ERpHER2n	ERnHER2n	ERpHER2n	ERpHER2n	ERpHER2n	ERpHER2n	ERpHER2n
Died of Other Causes	Died of Disease	Died of Disease	Living	Living	Died of Disease	Living	Died of Other Causes	Died of Other Causes	Died of Disease	Living
71.16666667	28.83333333	114.0333333	126.8666667	70.16666667	30.93333333	220.2333333	54.26666667	134.2666667	18.23333333	185.3
NO	NO	NO	NO	NO	YES	NO	NO	NO	NO	YES

MB_5161	MB_5292	MB_5211	MB_5218	MB_5209	MB_5227	MB_5213	MB_5166	MB_5163	MB_5188
WT	MUT	WT	WT	MUT	WT	MUT	MUT	WT	WT
WT	MISS	WT	WT	MISS	WT	MISS	MISS	WT	WT
	R175H			R110P		N131I	R280K		
ERpHER2n	HER2p	ERpHER2n	ERpHER2n	ERnHER2n	ERpHER2n	ERnHER2n	HER2p	ERpHER2n	ERpHER2n
Living	Died of Disease	Died of Other Causes	Living	Died of Other Causes	Died of Other Causes	Died of Disease	Died of Disease	Died of Other Causes	Died of Disease
175.3333333	48.43333333	247.8333333	254.9666667	175.1	187.0333333	43.4	80.73333333	212.2	19.4
NO	NO	NO	NO	NO	NO	YES	NO	NO	NO

MB_5143	MB_5329	MB_5322	MB_5310	MB_4000	MB_5240	MB_5228	MB_5117	MB_5176
WT	MUT	WT	WT	WT	WT	MUT	WT	WT
WT	MISS	WT	WT	WT	WT	NULL	WT	WT
	R280T					X225_splice		
ERpHER2n	ERpHER2n	ERpHER2n	ERpHER2n	ERpHER2n	ERpHER2n	ERpHER2n	ERpHER2n	ERpHER2n
Died of Other Causes	Died of Disease	Died of Disease	Died of Other Causes	Died of Other Causes	Died of Other Causes	Died of Other Causes	Living	Died of Other Causes
127.9333333	45.73333333	102.3	153.3	28.06666667	103.8333333	180.7333333	209.2666667	112.6333333
NO	YES	NO	NO	NO	NO	NO	NO	NO

MB_5215	MB_5223	MB_5179	MB_5330	MB_5144	MB_5196	MB_5200	MB_5583	MB_5601	MB_5632
WT	MUT	MUT	WT	WT	WT	WT	WT	WT	WT
WT	MISS	NULL	WT	WT	WT	WT	WT	WT	WT
	Y234C	R213*							
ERpHER2n	ERnHER2n	ERpHER2n	ERpHER2n	ERpHER2n	ERpHER2n	ERpHER2n	ERpHER2n	ERpHER2n	ERpHER2n
Died of Disease	Died of Disease	Died of Other Causes	Died of Other Causes	Living	Died of Disease	Died of Disease	Living	Died of Other Causes	Died of Disease
118.7	144.6666667	246.6	114.5333333	213.0333333	94.03333333	52.3	111.3666667	214.8	38.16666667
NO	YES	NO	NO	NO	NO	NO	NO	NO	NO

MB_5616	MB_5604	MB_5589	MB_5582	MB_5625	MB_5571	MB_5596	MB_5590	MB_5599	MB_5629	MB_5579
MUT	WT	WT	WT	MUT	WT	WT	MUT	WT	WT	WT
MISS	WT	WT	WT	NULL	WT	WT	MISS	WT	WT	WT
Y220C				p.E68X			S127F			
ERnHER2n	ERpHER2n	ERpHER2n	ERpHER2n	ERnHER2n	ERpHER2n	ERpHER2n	ERpHER2n	ERpHER2n	ERpHER2n	ERpHER2n
Living	Living	Living	Living	Died of Disease	Died of Disease	Died of Disease	Living	Living	Died of Other Causes	Died of Disease
180.6333333	145.6333333	185.1333333	222.3333333	32.3	153.8666667	167.9333333	17.83333333	224.8666667	103.8	90.6
YES	NO	NO	NO	YES	NO	NO	NO	NO	NO	NO

MB_5623	MB_5654	MB_5617	MB_5638	MB_5635	MB_5642	MB_5647	MB_4018
WT	WT	WT	WT	WT	WT	WT	WT
WT	WT	WT	WT	WT	WT	WT	WT
ERpHER2n	ERpHER2n	ERpHER2n	ERpHER2n	ERpHER2n	ERpHER2n	ERpHER2n	ERpHER2n
Died of Other Causes	Died of Other Causes	Died of Other Causes	Died of Other Causes	Died of Disease	Died of Other Causes	Died of Other Causes	Died of Disease
137.8	173.0333333	128.2	195.7	21.06666667	156.8	30.8	105.6666667
NO	NO	NO	NO	NO	NO	NO	NO

MB_5584	MB_5626	MB_5602	MB_5653	MB_5576	MB_5575	MB_5646	MB_5634	MB_5591	MB_5567
MUT	WT	MUT	WT	WT	WT	WT	MUT	WT	WT
NULL	WT	NULL	WT	WT	WT	WT	NULL	WT	WT
P301Qfs*44		W91*					N239*		
ERpHER2n	ERpHER2n	ERnHER2n	ERpHER2n	ERpHER2n	ERpHER2n	ERpHER2n	ERnHER2n	ERpHER2n	ERpHER2n
Died of Other Causes	Living	Living	Living	Living	Died of Other Causes	Died of Other Causes	Died of Disease	Died of Other Causes	Died of Other Causes
91.1	82.96666667	224.4333333	194.6	194	117.6666667	167.1	62.9	155.3666667	178.5666667
NO	NO	YES	NO	NO	NO	NO	NO	NO	NO

MB_5651	MB_5603	MB_5622	MB_5597	MB_5613	MB_5580	MB_5645	MB_5548	MB_5526	MB_5531
MUT	WT	WT	WT	MUT	WT	WT	MUT	MUT	MUT
MISS	WT	WT	WT	NULL	WT	WT	MISS	MISS	NULL
I195T				Y205_T211del			P278R	R273C	R333Vfs*12
ERnHER2n	ERpHER2n	ERpHER2n	ERpHER2n	ERpHER2n	ERpHER2n	ERpHER2n	ERnHER2n	ERnHER2n	ERnHER2n
Died of Disease	Died of Other Causes	Living	Died of Other Causes	Died of Other Causes	Died of Other Causes	Died of Other Causes	Living	Living	Living
21	171.6333333	182.9333333	32.73333333	212.3666667	71.63333333	22.23333333	25.33333333	151.0666667	183.9666667
YES	NO	NO	NO	NO	NO	NO	NO	NO	NO

MB_5468	MB_5474	MB_5514	MB_5302	MB_5510	MB_5473	MB_5484	MB_5432	MB_5525	MB_5562	MB_5497	MB_5551
MUT	MUT	WT	WT	WT	WT	WT	WT	MUT	WT	WT	MUT
NULL	MISS	WT	WT	WT	WT	WT	WT	MISS	WT	WT	NULL
S106Rfs*41	L111P							R248Q			R213*
HER2p	HER2p	ERpHER2n	ERpHER2n	ERpHER2n	ERpHER2n	ERpHER2n	ERpHER2n	ERpHER2n	ERpHER2n	ERpHER2n	ERnHER2n
Living	Living	Living	Living	Died of Disease	Died of Other Causes	Living	Died of Other Causes	Living	Died of Other Causes	Living	Living
99.36666667	226.7	182.9	191.1	103.1	125.9	238.5	98.5	2	102.9666667	237.5	182.2333333
YES	YES	NO	NO	NO	NO	NO	NO	NO	NO	NO	NO

MB_5300	MB_5560	MB_5550	MB_5520	MB_5529	MB_5554	MB_5301	MB_4421	MB_4408	MB_4146	MB_5534
WT	MUT	WT	MUT	MUT	WT	WT	WT	MUT	WT	WT
WT	MISS	WT	MISS	NULL	WT	WT	WT	NULL	WT	WT
	R175H		C141Y	C275Lfs*70				R196*		
ERpHER2n	ERnHER2n	ERpHER2n	ERpHER2n	ERnHER2n	ERpHER2n	ERpHER2n	ERpHER2n	ERnHER2n	ERnHER2n	ERpHER2n
Living	Living	Died of Other Causes	Died of Disease	Died of Disease	Living	Living	Died of Other Causes	Died of Other Causes	Died of Disease	Died of Disease
190.1666667	186.6	171.3	16.3	14.8	229.8333333	241.2333333	264.7666667	206.5666667	15.63333333	79.1
NO	NO	NO	NO	YES	NO	NO	NO	NO	YES	NO

MB_5521	MB_5482	MB_5483	MB_5532	MB_5556	MB_5518	MB_5486	MB_5511	MB_5422	MB_5535	MB_5477	MB_5472
WT	MUT	MUT	WT	WT	MUT	WT	MUT	WT	MUT	WT	WT
WT	MISS	MISS	WT	WT	NULL	WT	MISS	WT	NULL	WT	WT
	R337C	R248W			G374Afs*43		C238Y		X261_splice		
ERpHER2n	ERpHER2n	HER2p	ERpHER2n	ERpHER2n	ERpHER2n	ERpHER2n	ERnHER2n	ERpHER2n	HER2p	ERpHER2n	ERpHER2n
Living	Died of Disease	Died of Disease	Died of Disease	Living	Died of Disease	Died of Disease	Living	Died of Disease	Died of Disease	Living	Died of Disease
124.8	17.66666667	42.43333333	79.86666667	225.4	30.86666667	123.3	158.9666667	46.06666667	120.1333333	110.4666667	19.56666667
NO	YES	YES	NO	NO	NO	NO	NO	NO	YES	NO	NO

MB_5540	MB_5184	MB_5294	MB_5565	MB_5040	MB_4801	MB_5499	MB_5459	MB_5243
MUT	WT	MUT	MUT	WT	WT	WT	WT	MUT
MISS	WT	MISS	NULL	WT	WT	WT	WT	NULL
P278S		R175H	N200Ifs*47					G262_N263del
HER2p	ERpHER2n	ERnHER2n	ERnHER2n	ERpHER2n	ERpHER2n	ERpHER2n	HER2p	ERpHER2n
Died of Disease	Died of Other Causes	Living	Died of Other Causes	Died of Other Causes	Died of Other Causes	Living	Died of Disease	Died of Other Causes
48.43333333	89.33333333	195.9333333	194.1	79.16666667	130.3666667	123.7333333	90.8	38.43333333
NO	NO	YES	YES	NO	NO	NO	NO	NO

MB_4120	MB_4966	MB_5457	MB_4011	MB_5478	MB_4822	MB_4017	MB_5018	MB_4791
WT	WT	WT	MUT	WT	WT	WT	WT	MUT
WT	WT	WT	NULL	WT	WT	WT	WT	MISS
			X261_splice					E285K
ERpHER2n	ERpHER2n	ERpHER2n	ERpHER2n	ERpHER2n	ERpHER2n	ERpHER2n	ERpHER2n	ERpHER2n
Died of Other Causes	Died of Disease	Died of Disease	Living	Died of Other Causes	Died of Disease	Died of Other Causes	Died of Other Causes	Died of Other Causes
217.1	49.3	164.5666667	193.1666667	34.3	91.63333333	139.3	108.6	173.8333333
NO	NO	NO	NO	NO	NO	NO	NO	NO

MB_5118	MB_4806	MB_4970	MB_2964	MB_2963	MB_2957	MB_2954	MB_2916	MB_2725	MB_2711	MB_2730
WT	WT	WT	MUT	MUT	MUT	WT	WT	WT	WT	WT
WT	WT	WT	MISS	MISS	MISS	WT	WT	WT	WT	WT
			R248W	R267G	S241C					
ERpHER2n	ERpHER2n	ERpHER2n	ERpHER2n	ERnHER2n	ERnHER2n	ERpHER2n	ERpHER2n	ERpHER2n	ERpHER2n	ERpHER2n
Living	Died of Other Causes	Died of Disease	Died of Disease	Died of Disease	Living	Died of Disease	Living	Living	Living	Living
211.7333333	201.7666667	88.8	9.6	227.8333333	262.1333333	5.5	266.9333333	236.9333333	201.4666667	133.7333333
NO	NO	NO	NO	NO	YES	NO	NO	NO	NO	NO

MB_2669	MB_2705	MB_2728	MB_2708	MB_2735	MB_3035	MB_3064	MB_3060	MB_3049	MB_3037	MB_3063	MB_3083
WT	WT	WT	MUT	WT	WT	WT	WT	WT	WT	MUT	WT
WT	WT	WT	MISS	WT	WT	WT	WT	WT	WT	NULL	WT
			T155N							R213*	
ERpHER2n	ERpHER2n	ERpHER2n	ERpHER2n	ERpHER2n	ERpHER2n	ERpHER2n	ERpHER2n	ERpHER2n	ERpHER2n	ERnHER2n	ERpHER2n
Died of Disease	Living	Died of Disease	Died of Disease	Living	Living	Died of Disease	Died of Disease	Living	Living	Died of Disease	Died of Disease
73.7	163.7333333	51.4	133.2333333	274.5	260.7333333	165.1666667	44.6	259.9666667	98.83333333	28.56666667	81.1
NO	NO	YES	NO	YES	NO	NO	NO	NO	NO	YES	NO

MB_3046	MB_3067	MB_3351	MB_3367	MB_3361	MB_3412	MB_3371	MB_3429	MB_3360	MB_3403	MB_3350	MB_3355
MUT	MUT	WT	MUT	MUT	WT	WT	WT	MUT	WT	WT	MUT
NULL	MISS	WT	NULL	NULL	WT	WT	WT	MISS	WT	WT	NULL
L111Wfs*12	R175H		P191Lfs*53	R333Vfs*12				P250L			X187_splice
ERnHER2n	ERnHER2n	ERpHER2n	ERpHER2n	HER2p	ERpHER2n	ERpHER2n	ERpHER2n	HER2p	ERpHER2n	ERpHER2n	HER2p
Died of Other Causes	Living	Living	Living	Died of Disease	Living	Living	Living	Died of Disease	Living	Died of Other Causes	Living
73.83333333	259.5333333	241.8666667	179.4333333	68.2	228.8	241.2666667	243.5	26.76666667	138.3333333	227.8	109
NO	YES	NO	NO	NO	NO	NO	NO	NO	NO	NO	YES

MB_3329	MB_3467	MB_3341	MB_3298	MB_3357	MB_3388	MB_3365	MB_2749	MB_2754	MB_2752
MUT	MUT	WT	WT	MUT	MUT	WT	WT	WT	WT
MISS	NULL	WT	WT	MISS	MISS	WT	WT	WT	WT
G245V	C229Yfs*10			G245S	R249M				
HER2p	HER2p	ERpHER2n	ERpHER2n	ERpHER2n	ERpHER2n	ERpHER2n	ERpHER2n	ERnHER2n	ERpHER2n
Died of Disease	Died of Disease	Died of Disease	Living	Died of Disease	Died of Other Causes	Living	Died of Other Causes	Died of Disease	Died of Disease
37.96666667	38.8	148.7666667	242.5666667	38.03333333	228.6	241.6	97.76666667	203.5333333	81.93333333
NO	YES	NO	NO	NO	NO	NO	NO	NO	NO

MB_2745	MB_2747	MB_2760	MB_2742	MB_2750	MB_2753	MB_2966	MB_2953	MB_2969	MB_3014	MB_2947
WT	WT	WT	MUT	WT	MUT	WT	MUT	WT	MUT	WT
WT	WT	WT	MISS	WT	NULL	WT	MISS	WT	NULL	WT
			L111P		K139Nfs*9		Y234C		E339Gfs*6	
ERpHER2n	ERpHER2n	ERpHER2n	HER2p	ERpHER2n	ERnHER2n	ERpHER2n	ERpHER2n	ERpHER2n	ERnHER2n	ERpHER2n
Died of Disease	Died of Other Causes	Died of Disease	Died of Disease	Died of Other Causes	Living	Living	Died of Disease	Died of Other Causes	Living	Living
168.9666667	234.3333333	118.0333333	24.9	145.4333333	274.4	265	30.13333333	172.9	262.6333333	112
NO	NO	NO	YES	NO	YES	NO	NO	NO	YES	NO

MB_2951	MB_2960	MB_2970	MB_2971	MB_2933	MB_3479	MB_3490	MB_3487	MB_3459	MB_3476	MB_3492
WT	WT	MUT	WT	WT	WT	WT	WT	WT	MUT	WT
WT	WT	NULL	WT	WT	WT	WT	WT	WT	MISS	WT
		C124Lfs*25							C238G	
ERpHER2n	ERpHER2n	ERpHER2n	ERpHER2n	ERpHER2n	ERpHER2n	ERpHER2n	ERpHER2n	ERpHER2n	ERnHER2n	ERpHER2n
Living	Died of Other Causes	Died of Disease	Living	Died of Other Causes	Living	Died of Disease	Living	Living	Died of Other Causes	Died of Disease
263.3666667	222.2	252.8666667	264.2333333	161.9333333	231.6666667	102.4	237.1333333	241.4	159.0666667	118.1333333
NO	NO	NO	NO	NO	NO	NO	NO	NO	NO	NO

MB_3452	MB_3303	MB_3328	MB_3300	MB_3277	MB_3254	MB_3275	MB_3272	MB_3271	MB_3292	MB_3840
MUT	WT	WT	WT	MUT	WT	WT	MUT	MUT	MUT	MUT
NULL	WT	WT	WT	MISS	WT	WT	NULL	NULL	NULL	MISS
C229Yfs*10				R273C			I255Sfs*90	D49Mfs*4	R306*	R110H
ERpHER2n	ERpHER2n	ERpHER2n	ERpHER2n	ERnHER2n	ERpHER2n	ERpHER2n	HER2p	ERnHER2n	ERnHER2n	ERpHER2n
Died of Disease	Living	Died of Other Causes	Living	Died of Disease	Living	Died of Disease	Died of Disease	Died of Other Causes	Living	Living
119.8666667	243.9	191.1333333	247	32.93333333	123.9	41.53333333	22.66666667	65.46666667	217.7666667	287.2333333
NO	NO	NO	NO	YES	NO	NO	NO	NO	NO	NO

MB_2536	MB_2564	MB_2513	MB_2617	MB_2614	MB_2632	MB_2556	MB_2624	MB_2867	MB_2922	MB_2917
WT	WT	MUT	MUT	WT	MUT	WT	WT	WT	MUT	MUT
WT	WT	MISS	MISS	WT	MISS	WT	WT	WT	NULL	NULL
		L344R	H179R		G199V				P152Rfs*18	X126_splice
ERpHER2n	ERpHER2n	HER2p	ERpHER2n	ERpHER2n	HER2p	ERnHER2n	ERpHER2n	ERpHER2n	ERnHER2n	ERnHER2n
Died of Other Causes	Living	Died of Disease	Died of Disease	Died of Disease	Died of Disease	Died of Other Causes	Living	Living	Died of Disease	Died of Disease
47.9	285.4333333	59.7	89.1	64.93333333	19.1	220.9	128.5333333	269.3333333	14.4	31.93333333
NO	NO	NO	NO	YES	YES	NO	NO	NO	YES	YES

MB_2858	MB_2854	MB_2853	MB_3235	MB_3165	MB_3171	MB_3167	MB_3222	MB_3706	MB_3497	MB_3488
MUT	MUT	WT	MUT	MUT	WT	WT	WT	MUT	WT	MUT
MISS	MISS	WT	MISS	NULL	WT	WT	WT	NULL	WT	NULL
R267P	F134L		R248W	R213*				R213*		Q52Lfs*68
ERpHER2n	ERpHER2n	ERpHER2n	HER2p	ERpHER2n	ERpHER2n	ERpHER2n	ERpHER2n	ERnHER2n	HER2p	HER2p
Died of Other Causes	Living	Living	Living	Living	Died of Disease	Living	Died of Other Causes	Died of Disease	Died of Disease	Died of Disease
141.5666667	270.4333333	269.6333333	236.1333333	221.2333333	219.6666667	136.9333333	23.93333333	38.8	15.5	27.46666667
NO	NO	NO	NO	YES	NO	NO	NO	NO	YES	YES

MB_3711	MB_3548	MB_3506	MB_3525	MB_3606	MB_3453	MB_3545	MB_3470	MB_2765	MB_2763	MB_2767	MB_2790
WT	WT	WT	WT	MUT	MUT	MUT	MUT	WT	WT	WT	WT
WT	WT	WT	WT	NULL	MISS	NULL	MISS	WT	WT	WT	WT
				Q331Rfs*14	G262V	D393Tfs*29	R248G				
ERpHER2n	ERpHER2n	ERpHER2n	ERpHER2n	HER2p	ERnHER2n	ERpHER2n	HER2p	ERpHER2n	ERpHER2n	ERpHER2n	ERpHER2n
Living	Died of Other Causes	Living	Living	Living	Died of Disease	Died of Disease	Died of Disease	Died of Other Causes	Living	Living	Living
222.1	234.5333333	240.8333333	236.7	110.6333333	32.83333333	232.7333333	62.53333333	227.8666667	275.6	271.3333333	116.9333333
NO	NO	NO	NO	NO	YES	NO	YES	NO	NO	NO	NO

MB_2769	MB_2842	MB_2772	MB_2779	MB_2774	MB_2983	MB_3005	MB_2977	MB_2996	MB_2999	MB_2994
WT	MUT	WT	MUT	WT	MUT	WT	WT	WT	WT	WT
WT	MISS	WT	MISS	WT	NULL	WT	WT	WT	WT	WT
	L194R		G245C		Y107*					
ERpHER2n	ERnHER2n	ERpHER2n	ERpHER2n	ERpHER2n	HER2p	ERpHER2n	ERpHER2n	ERpHER2n	ERpHER2n	HER2p
Living	Died of Disease	Died of Other Causes	Living	Died of Other Causes	Died of Other Causes	Living	Died of Other Causes	Living	Living	Living
160	19.73333333	150.7333333	275.2333333	131.6666667	180.8333333	253.0666667	110.6	191.2333333	262	254.5
NO	YES	NO	NO	NO	NO	NO	NO	NO	NO	NO

MB_3025	MB_2984	MB_3002	MB_2845	MB_2912	MB_2844	MB_2847	MB_2849	MB_2851	MB_2848	MB_2863	MB_2840
MUT	MUT	WT	WT	MUT	MUT	MUT	MUT	MUT	WT	WT	WT
NULL	MISS	WT	WT	NULL	NULL	NULL	NULL	NULL	WT	WT	WT
N239*	N239S			V157Tfs*26	I162delinsRL	X225_splice	T155Pfs*23	E294*			
HER2p	HER2p	ERpHER2n	ERpHER2n	ERnHER2n	ERpHER2n	HER2p	ERnHER2n	ERpHER2n	ERpHER2n	ERpHER2n	ERpHER2n
Living	Living	Died of Other Causes	Living	Living	Living	Died of Disease	Died of Disease	Died of Disease	Died of Other Causes	Living	Living
256	261.2	140.5666667	264.7666667	267.4	146.7333333	39.53333333	43.2	159	227.7333333	258.3333333	269
YES	NO	NO	NO	YES	NO	NO	NO	NO	NO	NO	NO

MB_2843	MB_3013	MB_3058	MB_3016	MB_3057	MB_3008	MB_3021	MB_3032	MB_3007	MB_3033	MB_3026
MUT	WT	WT	MUT	MUT	WT	WT	WT	WT	WT	WT
MISS	WT	WT	NULL	NULL	WT	WT	WT	WT	WT	WT
S241C			R342Efs*3	A138Pfs*32						
ERpHER2n	ERpHER2n	ERnHER2n	ERpHER2n	ERnHER2n	ERpHER2n	ERpHER2n	ERpHER2n	ERpHER2n	ERpHER2n	ERpHER2n
Died of Other Causes	Living	Living	Living	Died of Disease	Living	Died of Other Causes	Living	Died of Disease	Died of Disease	Living
231.5333333	238.3666667	254.2666667	258.8666667	32.03333333	149.6	217.5666667	250.1333333	114.9	70.4	261.2
NO	NO	YES	NO	YES	NO	NO	NO	NO	NO	NO

MB_3006	MB_3439	MB_3437	MB_3395	MB_3386	MB_3382	MB_3379	MB_3430	MB_3389	MB_3378	MB_3110
WT	WT	WT	MUT	MUT	WT	WT	WT	WT	WT	WT
WT	WT	WT	MISS	MISS	WT	WT	WT	WT	WT	WT
			Y220C	R248Q						
ERpHER2n	ERpHER2n	ERpHER2n	ERnHER2n	HER2p	HER2p	HER2p	ERpHER2n	ERpHER2n	ERpHER2n	ERpHER2n
Died of Disease	Living	Living	Living	Died of Disease	Living	Died of Other Causes	Died of Other Causes	Living	Died of Other Causes	Living
15.7	188.7333333	108.7666667	243.7666667	55.63333333	136.2333333	113.8333333	219.7666667	240.7	87.53333333	100.7333333
NO	NO	NO	NO	YES	NO	NO	NO	NO	NO	NO

MB_3079	MB_3103	MB_3102	MB_3085	MB_3122	MB_3104	MB_3123	MB_3121	MB_2835	MB_2838	MB_2801
WT	WT	WT	WT	MUT	WT	MUT	WT	WT	WT	MUT
WT	WT	WT	WT	MISS	WT	MISS	WT	WT	WT	NULL
				P278T		S241Y				R213*
ERpHER2n	HER2p	ERpHER2n	ERpHER2n	ERpHER2n	HER2p	ERnHER2n	ERpHER2n	ERpHER2n	ERpHER2n	ERpHER2n
Living	Died of Disease	Living	Died of Disease	Living	Died of Disease	Living	Died of Other Causes	Died of Other Causes	Living	Living
149.6	28.73333333	258.3333333	210.9666667	257.7666667	75.36666667	167.4333333	131.3333333	55.03333333	270.3	271.2666667
NO	NO	NO	NO	NO	NO	YES	NO	NO	NO	NO

MB_2834	MB_2796	MB_2857	MB_2819	MB_2781	MB_2793	MB_2827	MB_2814	MB_2791	MB_2803	MB_2797
MUT	MUT	MUT	WT	WT	WT	MUT	WT	WT	WT	WT
MISS	MISS	NULL	WT	WT	WT	NULL	WT	WT	WT	WT
I232T	Y220S	R306*				X224_splice				
ERnHER2n	ERpHER2n	ERnHER2n	ERpHER2n	ERpHER2n	ERpHER2n	ERnHER2n	ERpHER2n	ERpHER2n	ERpHER2n	ERpHER2n
Died of Other Causes	Died of Disease	Living	Living	Died of Other Causes	Living	Living	Died of Other Causes	Living	Died of Disease	Living
153.8333333	87.7	250.6666667	270.5666667	40.43333333	267.2333333	235.6666667	231.0333333	271.9333333	85.86666667	252.9666667
NO	NO	YES	NO	NO	NO	YES	NO	NO	NO	NO

MB_2795	MB_2786	MB_3850	MB_3838	MB_3707	MB_3600	MB_3824	MB_3823	MB_3781	MB_2642	MB_2686	MB_2616	MB_2634
WT	WT	WT	WT	MUT	WT	MUT	WT	WT	WT	WT	MUT	WT
WT	WT	WT	WT	MISS	WT	NULL	WT	WT	WT	WT	MISS	WT
				E285K		I162Tfs*14					R248W	
ERpHER2n	HER2p	HER2p	ERpHER2n	ERpHER2n	ERpHER2n	ERpHER2n	ERpHER2n	ERpHER2n	ERpHER2n	ERpHER2n	ERpHER2n	ERpHER2n
Living	Died of Disease	Living	Died of Disease	Died of Disease	Living	Died of Disease	Living	Living	Died of Disease	Living	Died of Disease	Living
272.1	68.26666667	72.3	134.4666667	228.7666667	214.7	126.4666667	222.5	224.5666667	45.16666667	177.2666667	219.1666667	274.0333333
NO	NO	NO	NO	NO	NO	NO	NO	NO	YES	NO	NO	NO

MB_2613	MB_3842	MB_3866	MB_3874	MB_3871	MB_3702	MB_3105	MB_3852	MB_3854	MB_3865	MB_3228	MB_3028	MB_3502
WT	WT	MUT	MUT	WT	MUT	WT	MUT	WT	WT	WT	MUT	MUT
WT	WT	NULL	MISS	WT	MISS	WT	MISS	WT	WT	WT	MISS	MISS
		R196*	R110H		L257Q		L194R				R248Q	R273H
ERpHER2n	ERpHER2n	HER2p	ERpHER2n	ERpHER2n	ERnHER2n	ERpHER2n	HER2p	ERpHER2n	ERpHER2n	ERpHER2n	HER2p	ERnHER2n
Living	Living	Living	Living	Died of Other Causes	Living	Living	Living	Living	Living	Living	Died of Disease	Living
163.4	226.0666667	225.5	186.4333333	172.8	230.4666667	252.3333333	225.5	99.23333333	199.9666667	252	42.56666667	229.3333333
NO	NO	NO	NO	NO	YES	NO	NO	NO	NO	NO	NO	YES

MB_3567	MB_3752	MB_3450	MB_0476	MB_0610	MB_0451	MB_0133	MB_0048	MB_0083	MB_0053	MB_0056	MB_0068	MB_0079
MUT	MUT	WT	MUT	WT	WT	WT	WT	WT	WT	WT	WT	MUT
MISS	NULL	WT	MISS	WT	WT	WT	WT	WT	WT	WT	WT	MISS
Y126N	R196*		R175H									R273C
ERnHER2n	ERnHER2n	ERpHER2n	ERpHER2n	ERpHER2n	ERpHER2n	ERpHER2n	HER2p	ERpHER2n	ERpHER2n	ERpHER2n	ERpHER2n	ERnHER2n
Living	Died of Disease	Living	Living	Living	Living	Living	Living	Died of Disease	Living	Living	Living	Died of Disease
236.0333333	75.33333333	240.4666667	130.4333333	76.7	50.06666667	151	103.8333333	86.06666667	161.0666667	62.86666667	103.1333333	28.5
YES	NO	NO	YES	NO	NO	YES	YES	NO	NO	NO	NO	YES

MB_0108	MB_0006	MB_0014	MB_0022	MB_0039	MB_0062	MB_0054	MB_0081	MB_0071	MB_0099	MB_0064	MB_0107
MUT	WT	WT	WT	WT	MUT	WT	WT	WT	WT	WT	MUT
MISS	WT	WT	WT	WT	NULL	WT	WT	WT	WT	WT	MISS
G245S					S303Efs*3						P177R
ERpHER2n	ERpHER2n	ERpHER2n	ERpHER2n	ERpHER2n	ERnHER2n	ERpHER2n	ERpHER2n	ERpHER2n	ERpHER2n	ERpHER2n	ERpHER2n
Died of Disease	Living	Living	Died of Other Causes	Living	Living	Living	Living	Died of Other Causes	Died of Disease	Living	Living
42.7	164.9333333	164.3333333	99.53333333	163.5333333	153.9666667	160.3	69.5	131	132.1	108.9333333	158.0333333
YES	YES	YES	NO	NO	YES	NO	NO	NO	YES	NO	NO

MB_4681	MB_4626	MB_4639	MB_4673	MB_4711	MB_4607	MB_4669	MB_4015	MB_2931	MB_2927
WT	WT	WT	WT	MUT	WT	WT	MUT	MUT	WT
WT	WT	WT	WT	NULL	WT	WT	NULL	NULL	WT
				W91*			E180*	H178Pfs*3	
ERpHER2n	HER2p	ERpHER2n	ERpHER2n	HER2p	ERpHER2n	ERpHER2n	ERnHER2n	ERpHER2n	ERpHER2n
Died of Disease	Died of Disease	Died of Disease	Died of Disease	Died of Disease	Living	Died of Other Causes	Died of Disease	Died of Other Causes	Died of Disease
251.8	143	119	87	31.46666667	121.7333333	119.4666667	11.3	228.1	263.7
NO	NO	NO	NO	YES	NO	NO	NO	NO	NO

MB_2919	MB_2944	MB_2929	MB_2932	MB_2724	MB_2712	MB_3062	MB_3050	MB_4722	MB_4731
WT	MUT	MUT	WT	WT	WT	MUT	MUT	WT	MUT
WT	MISS	MISS	WT	WT	WT	NULL	NULL	WT	MISS
	S127F	R181C				N239_S240insT	Q192*		R282W
ERpHER2n	ERpHER2n	ERnHER2n	ERpHER2n	ERnHER2n	ERpHER2n	ERnHER2n	ERpHER2n	ERpHER2n	HER2p
Died of Other Causes	Died of Disease	Died of Other Causes	Living	Died of Disease	Died of Other Causes	Living	Died of Other Causes	Living	Died of Disease
240.0333333	27.2	262.8666667	108.4333333	108.0666667	234.4333333	146.3666667	255.1	86.1	44.86666667
NO	NO	YES	NO	NO	NO	NO	NO	NO	YES

MB_4725	MB_4707	MB_4643	MB_3363	MB_3383	MB_3344	MB_3381	MB_3297	MB_3301	MB_3088	MB_2764	MB_2744
MUT	MUT	MUT	WT	MUT	WT	WT	MUT	WT	WT	WT	WT
MISS	MISS	MISS	WT	MISS	WT	WT	MISS	WT	WT	WT	WT
R273G	T125K	R342P		R175H			R342P				
HER2p	ERnHER2n	HER2p	ERpHER2n	ERnHER2n	ERpHER2n	ERpHER2n	ERnHER2n	ERpHER2n	ERpHER2n	ERpHER2n	ERpHER2n
Died of Disease	Living	Died of Disease	Died of Disease	Died of Disease	Living	Living	Living	Living	Living	Died of Disease	Living
98.76666667	221.2	41.46666667	10.86666667	23.2	241.2666667	236.6333333	236.0666667	248.7666667	256.5	270.1333333	275.6333333
NO	NO	NO	NO	YES	NO	NO	YES	NO	NO	NO	NO

MB_7114	MB_7118	MB_7113	MB_7130	MB_7149	MB_7140	MB_7148	MB_7208	MB_7170	MB_7174	MB_7252
MUT	WT	WT	WT	WT	WT	WT	WT	WT	MUT	MUT
NULL	WT	WT	WT	WT	WT	WT	WT	WT	MISS	NULL
C242Afs*5									Y220C	F113del
ERnHER2n	ERpHER2n	ERpHER2n	ERpHER2n	ERpHER2n	ERpHER2n	ERpHER2n	ERnHER2n	ERpHER2n	ERpHER2n	ERnHER2n
Living	Living	Died of Other Causes	Died of Disease	Died of Other Causes	Died of Disease	Died of Disease	Died of Disease	Living	Living	Died of Other Causes
123.2666667	140.5666667	136.7	102.9666667	44.63333333	90.8	19	45.93333333	155.4	140.7666667	35.7
NO	NO	NO	NO	NO	NO	YES	YES	NO	NO	NO

MB_7244	MB_7187	MB_7251	MB_7173	MB_3001	MB_2952	MB_7097	MB_7104	MB_7099	MB_7069
WT	MUT	WT	WT	MUT	WT	WT	MUT	WT	MUT
WT	MISS	WT	WT	MISS	WT	WT	MISS	WT	NULL
	C242S			R273H			R175H		P153Afs*28
ERpHER2n	HER2p	HER2p	ERpHER2n	ERnHER2n	ERpHER2n	ERpHER2n	ERpHER2n	ERpHER2n	HER2p
Living	Died of Disease	Died of Disease	Died of Other Causes	Living	Died of Other Causes	Died of Disease	Died of Other Causes	Died of Other Causes	Living
184.1666667	146.8333333	21.56666667	63.53333333	263.2333333	189.1	65.16666667	122.8333333	163.1666667	114.6
NO	YES	NO	NO	YES	NO	YES	NO	NO	NO

MB_7066	MB_7100	MB_7067	MB_7073	MB_7063	MB_7102	MB_7068	MB_7075	MB_7053	MB_7074	MB_7057
WT	WT	MUT	MUT	WT	WT	MUT	WT	MUT	WT	MUT
WT	WT	MISS	NULL	WT	WT	MISS	WT	NULL	WT	MISS
		L194R	G302Rfs*4			R273C		R306*		S241F
ERnHER2n	ERpHER2n	HER2p	HER2p	ERpHER2n	ERpHER2n	HER2p	ERpHER2n	ERpHER2n	ERpHER2n	ERnHER2n
Living	Died of Other Causes	Living	Died of Other Causes	Living	Died of Other Causes	Living	Living	Living	Living	Died of Disease
89.76666667	156.3333333	105	137.9333333	86.6	104.1	98.46666667	111.6333333	122.1333333	108.1666667	43.83333333
YES	NO	NO	YES	NO	NO	NO	NO	NO	NO	YES

MB_7109	MB_7051	MB_7071	MB_7106	MB_7059	MB_7107	MB_7076	MB_7111	MB_7015	MB_7026	MB_7013
WT	MUT	WT	WT	MUT	WT	WT	MUT	WT	WT	WT
WT	MISS	WT	WT	NULL	WT	WT	MISS	WT	WT	WT
	P278S			E298*			R273H			
ERpHER2n	ERpHER2n	ERpHER2n	ERpHER2n	HER2p	ERpHER2n	ERpHER2n	ERpHER2n	ERpHER2n	ERpHER2n	ERpHER2n
Living	Died of Disease	Living	Died of Disease	Living	Died of Disease	Died of Other Causes	Died of Disease	Living	Died of Other Causes	Living
117.5333333	85.93333333	102.0666667	37.5	119.3333333	56.93333333	142.6666667	30.16666667	78.16666667	111.7	46.43333333
NO	NO	NO	NO	YES	NO	NO	NO	NO	NO	NO

MB_7023	MB_7024	MB_7014	MB_7017	MB_7019	MB_3510	MB_3500	MB_3396	MB_3435	MB_7186	MB_7142
MUT	WT	MUT	MUT	WT	WT	MUT	MUT	WT	WT	WT
MISS	WT	NULL	MISS	WT	WT	NULL	NULL	WT	WT	WT
H193R		M44Cfs*79	A159V			W53*	A69Vfs*54			
ERnHER2n	ERpHER2n	ERpHER2n	ERnHER2n	ERpHER2n	ERpHER2n	ERnHER2n	ERnHER2n	HER2p	ERpHER2n	ERpHER2n
Died of Disease	Living	Living	Living	Died of Other Causes	Died of Other Causes	Living	Living	Died of Disease	Living	Died of Other Causes
27.96666667	70.26666667	68.7	135.3333333	80.73333333	59.6	239.3	226.7333333	25.43333333	4.866666667	175.9
YES	NO	NO	YES	NO	NO	NO	YES	YES	NO	NO

MB_7196	MB_7171	MB_7145	MB_7132	MB_7189	MB_7137	MB_7181	MB_7155	MB_7138	MB_3295	MB_3218
MUT	WT	MUT	WT	MUT	WT	WT	MUT	WT	MUT	MUT
NULL	WT	NULL	WT	NULL	WT	WT	NULL	WT	MISS	MISS
X224_splice		E51*		P177_C182del			C242Afs*5		P278R	L137Q
ERpHER2n	ERpHER2n	ERnHER2n	ERpHER2n	ERpHER2n	ERpHER2n	ERpHER2n	ERnHER2n	ERpHER2n	ERpHER2n	ERnHER2n
Died of Disease	Died of Other Causes	Living	Living	Died of Disease	Living	Living	Living	Died of Other Causes	Died of Other Causes	Living
107.1	160.4	138.3333333	131.6	143.6	179.8	157.5333333	149.7666667	123	158.5333333	248.7666667
NO	NO	YES	NO	YES	NO	NO	YES	NO	NO	YES

MB_7232	MB_7281	MB_7270	MB_7231	MB_7220	MB_7277	MB_7280	MB_7227	MB_7278	MB_2610
WT	MUT	MUT	WT	WT	WT	WT	WT	WT	WT
WT	MISS	NULL	WT	WT	WT	WT	WT	WT	WT
	R175G	N131del							
ERpHER2n	HER2p	ERnHER2n	ERpHER2n	ERpHER2n	ERpHER2n	ERpHER2n	ERpHER2n	ERpHER2n	ERpHER2n
Living	Died of Disease	Living	Died of Other Causes	Died of Other Causes	Living	Living	Died of Other Causes	Died of Other Causes	Living
177.6	49.53333333	175.1666667	199.0333333	183.2	187.0333333	195.5333333	63.2	241.3	280.7
NO	YES	YES	NO	NO	NO	NO	NO	NO	NO

MB_2517	MB_2896	MB_2901	MB_2895	MB_2900	MB_2939	MB_7008	MB_7002	MB_7000	MB_7006
MUT	WT	WT	MUT	MUT	WT	WT	WT	WT	WT
MISS	WT	WT	NULL	MISS	WT	WT	WT	WT	WT
L111P			R306*	K132N					
HER2p	ERpHER2n	ERpHER2n	HER2p	ERpHER2n	HER2p	ERnHER2n	ERpHER2n	ERpHER2n	ERpHER2n
Died of Other Causes	Died of Other Causes	Died of Disease	Died of Disease	Died of Other Causes	Died of Other Causes	Died of Other Causes	Living	Living	Living
268.4333333	237.3666667	218.3	196.5666667	20.66666667	101.2666667	50.9	57.93333333	97.4	109.9333333
NO	NO	NO	NO	NO	NO	NO	NO	NO	NO

MB_7007	MB_7011	MB_7001	MB_7009	MB_7003	MB_7005	MB_7010	MB_3153	MB_3211	MB_3181	MB_3252	MB_3266
MUT	WT	WT	WT	WT	WT	WT	MUT	WT	MUT	WT	MUT
MISS	WT	WT	WT	WT	WT	WT	MISS	WT	NULL	WT	MISS
E224D							S241F		R342*		Y220C
ERnHER2n	ERpHER2n	HER2p	ERnHER2n	ERpHER2n	ERpHER2n	ERpHER2n	ERnHER2n	ERnHER2n	ERpHER2n	ERpHER2n	ERpHER2n
Living	Died of Other Causes	Living	Died of Disease	Died of Other Causes	Living	Living	Living	Living	Died of Disease	Living	Died of Disease
58.6	70.06666667	102.7	54.76666667	91.23333333	45.33333333	74.03333333	227.9333333	145.5	178.1666667	99.7	51.2
YES	NO	NO	YES	NO	NO	NO	YES	NO	NO	NO	NO

MB_7082	MB_7093	MB_7083	MB_7091	MB_7065	MB_7085	MB_7096	MB_7018	MB_7088	MB_7055	MB_7080	MB_7161
MUT	WT	MUT	MUT	WT	WT	WT	WT	MUT	MUT	WT	WT
MISS	WT	MISS	MISS	WT	WT	WT	WT	MISS	NULL	WT	WT
C135W		G154V	Y236C					M246V	R306*		
HER2p	ERpHER2n	ERpHER2n	ERpHER2n	ERpHER2n	ERpHER2n	ERpHER2n	ERpHER2n	HER2p	ERnHER2n	ERpHER2n	ERpHER2n
Living	Died of Other Causes	Living	Living	Living	Living	Living	Died of Disease	Living	Died of Disease	Living	Living
98.26666667	125.8333333	105.4666667	110.2666667	107.8666667	110.1	124	86.53333333	123.5333333	9.133333333	91.23333333	121.5333333
YES	YES	YES	NO	NO	NO	NO	YES	NO	NO	NO	NO

MB_7115	MB_7123	MB_7176	MB_7121	MB_7172	MB_7164	MB_7127	MB_7128	MB_7157	MB_7092	MB_7135
WT	WT	WT	WT	WT	WT	WT	WT	WT	WT	WT
WT	WT	WT	WT	WT	WT	WT	WT	WT	WT	WT
HER2p	ERpHER2n	ERpHER2n	ERnHER2n	ERpHER2n	ERpHER2n	ERpHER2n	HER2p	ERpHER2n	ERpHER2n	HER2p
Died of Other Causes	Living	Died of Other Causes	Died of Other Causes	Living	Living	Living	Died of Other Causes	Living	Living	Died of Disease
141.7333333	137.6666667	194.4	88.83333333	149.3	211.4	135.3333333	60.26666667	219.6333333	122.4	26.73333333
NO	NO	NO	YES	NO	NO	NO	NO	NO	NO	YES

MB_3754	MB_3536	MB_3748	MB_3530	MB_3582	MB_3528	MB_3576	MB_2770	MB_2758	MB_2771
WT	MUT	MUT	WT	WT	WT	MUT	WT	MUT	MUT
WT	NULL	NULL	WT	WT	WT	NULL	WT	MISS	NULL
	Q192*	R306*				M340Ifs*7		V197E	L111Ffs*40
ERpHER2n	ERpHER2n	ERpHER2n	ERpHER2n	ERpHER2n	HER2p	ERpHER2n	ERpHER2n	HER2p	ERnHER2n
Died of Disease	Died of Disease	Living	Died of Other Causes	Died of Other Causes	Living	Died of Disease	Living	Died of Disease	Died of Disease
92.73333333	86.23333333	228	112.9666667	229.3666667	113.6666667	24.86666667	274.3666667	35	44.83333333
NO	NO	NO	NO	NO	NO	NO	NO	YES	NO

MB_7152	MB_7143	MB_7197	MB_7153	MB_7144	MB_7185	MB_7199	MB_7158	MB_7163
WT	WT	WT	WT	MUT	WT	WT	MUT	WT
WT	WT	WT	WT	MISS	WT	WT	MISS	WT
				R282W			R175H	
ERpHER2n	HER2p	ERpHER2n	ERpHER2n	ERpHER2n	ERpHER2n	ERpHER2n	ERnHER2n	ERpHER2n
Died of Other Causes	Living	Died of Other Causes	Living	Died of Other Causes	Died of Disease	Died of Other Causes	Died of Disease	Died of Disease
195.8	186.3666667	187.8666667	142.8	102.6333333	125.0333333	76	46.43333333	52.63333333
NO	NO	NO	NO	NO	NO	NO	YES	YES

MB_7215	MB_7200	MB_7193	MB_7188	MB_7198	MB_7256	MB_7258	MB_7194	MB_7254
WT	WT	WT	WT	MUT	MUT	MUT	WT	WT
WT	WT	WT	WT	NULL	MISS	NULL	WT	WT
				K291*	F134C	L194Pfs*13		
ERpHER2n	ERpHER2n	ERpHER2n	ERpHER2n	ERpHER2n	HER2p	ERnHER2n	ERpHER2n	ERpHER2n
Died of Other Causes	Died of Other Causes	Died of Other Causes	Died of Other Causes	Died of Disease	Died of Disease	Died of Disease	Died of Disease	Living
208.9666667	97.6	87.46666667	188.1666667	16.73333333	34.7	15.06666667	46.66666667	192.2
NO	NO	NO	NO	NO	YES	YES	YES	NO

MB_7262	MB_2990	MB_2993	MB_3031	MB_2904	MB_2846	MB_7154	MB_7229	MB_7151	MB_7228	MB_7218
MUT	WT	WT	WT	MUT	MUT	MUT	WT	MUT	WT	WT
MISS	WT	WT	WT	NULL	MISS	MISS	WT	NULL	WT	WT
G262V				R110Lfs*13	Y234N	R273H		R196*		
ERpHER2n	ERpHER2n	ERnHER2n	HER2p	ERnHER2n	ERpHER2n	ERnHER2n	ERpHER2n	ERnHER2n	ERpHER2n	ERpHER2n
Died of Other Causes	Living	Living	Died of Disease	Died of Disease	Died of Disease	Died of Other Causes	Died of Other Causes	Living	Living	Living
104.0333333	260	187.0333333	35.63333333	125.6	149.7	108.4666667	111.6	144.4333333	239.1666667	165.4333333
NO	NO	NO	YES	YES	NO	NO	NO	NO	NO	NO

MB_7147	MB_7219	MB_7165	MB_7150	MB_3436	MB_3462	MB_3417	MB_2721	MB_3092	MB_2821	MB_2850
WT	WT	MUT	MUT	WT	WT	WT	WT	WT	WT	WT
WT	WT	MISS R175H	MISS R175H	WT	WT	WT	WT	WT	WT	WT
ERpHER2n	ERpHER2n	ERnHER2n	ERpHER2n	ERpHER2n	ERpHER2n	ERpHER2n	ERpHER2n	ERpHER2n	ERnHER2n	ERnHER2n
Died of Other Causes	Living	Living	Living	Died of Disease	Living	Living	Living	Died of Other Causes	Died of Other Causes	Died of Disease
168.6	165.2	147.1666667	128.9666667	50.76666667	234.2333333	103.4666667	251.6333333	152.3333333	136.0666667	9.433333333
NO	NO	YES	NO	YES	NO	NO	NO	NO	NO	YES

MB_2820	MB_2823	MB_2792	MB_7273	MB_7283	MB_7284	MB_7279	MB_7233	MB_7287	MB_7250	MB_7238
WT	WT	WT	MUT	MUT	WT	MUT	WT	MUT	MUT	MUT
WT	WT	WT	MISS	NULL	WT	MISS	WT	MISS	NULL	NULL
			E258Q	E349*		L330R		R273H	A83Gfs*66	X224_splice
ERpHER2n	HER2p	ERpHER2n	HER2p	ERpHER2n	ERpHER2n	ERpHER2n	ERpHER2n	HER2p	HER2p	ERpHER2n
Living	Living	Died of Disease	Living	Died of Disease	Died of Other Causes	Died of Disease	Died of Other Causes	Died of Disease	Died of Disease	Died of Disease
254.6333333	269.9	110.7666667	208.2	28.86666667	203	21.3	222.0333333	96.96666667	51	23.26666667
NO	NO	NO	YES	YES	NO	NO	NO	NO	NO	NO

MB_7285	MB_7260	MB_7264	MB_7268	MB_7276	MB_7212	MB_7267	MB_7266	MB_7216	MB_7195
MUT	MUT	MUT	WT	WT	WT	MUT	WT	WT	WT
MISS	NULL	NULL	WT	WT	WT	NULL	WT	WT	WT
T211A	G325*	X125_splice				C229Yfs*10			
ERpHER2n	HER2p	ERpHER2n	ERpHER2n	ERpHER2n	ERpHER2n	ERnHER2n	ERpHER2n	ERpHER2n	ERpHER2n
Died of Disease	Living	Died of Disease	Living	Living	Died of Other Causes	Died of Other Causes	Died of Disease	Died of Other Causes	Died of Other Causes
109	31.16666667	42.96666667	227.4666667	185.3333333	52.5	195.3666667	68.16666667	207.4666667	80.43333333
NO	YES	NO	NO	NO	NO	YES	NO	NO	NO

MB_7201	MB_7217	MB_2778	MB_2815	MB_2833	MB_3614	MB_7052	MB_7124	MB_7122	MB_7133	MB_7084	MB_7101
MUT	WT	WT	WT	MUT	WT	MUT	WT	MUT	WT	MUT	WT
MISS	WT	WT	WT	MISS	WT	NULL	WT	MISS	WT	NULL	WT
K132N				R175H		P177_C182del		R248W		I50*	
ERnHER2n	ERpHER2n	ERpHER2n	ERpHER2n	ERnHER2n	ERpHER2n	ERnHER2n	ERpHER2n	ERpHER2n	ERpHER2n	HER2p	ERpHER2n
Living	Died of Other Causes	Died of Disease	Living	Living	Living	Living	Living	Died of Other Causes	Died of Disease	Living	Living
163.4333333	90.4	181.2333333	257.5666667	259.7666667	235.4	106.1333333	139.6	29.9	23.83333333	116.5333333	124.2666667
NO	NO	NO	NO	YES	NO	NO	NO	NO	NO	NO	NO

MB_7116	MB_7131	MB_7090	MB_7095	MB_7094	MB_7087	MB_7077	MB_7072	MB_7041	MB_7044	MB_7022	MB_7070
WT	WT	MUT	WT	WT	MUT	WT	MUT	WT	WT	WT	MUT
WT	WT	NULL	WT	WT	NULL	WT	NULL	WT	WT	WT	NULL
		E294*			X261_splice		E349*				N239_S240del
ERpHER2n	ERpHER2n	ERnHER2n	ERpHER2n	ERpHER2n	ERnHER2n	ERpHER2n	ERpHER2n	ERpHER2n	ERpHER2n	ERpHER2n	ERpHER2n
Died of Disease	Living	Living	Living	Living	Died of Other Causes	Living	Died of Disease	Died of Other Causes	Living	Living	Living
33.36666667	134.7333333	109.7666667	115.6333333	113.3666667	5.066666667	101.2666667	52.33333333	101.9666667	86.83333333	85.5	108.0666667
NO	NO	YES	NO	NO	YES	NO	NO	NO	NO	NO	NO

MB_7079	MB_7025	MB_7062	MB_7036	MB_7046	MB_4839	MB_5559	MB_5463	MB_4744	MB_4769	MB_4484
WT	MUT	WT	MUT	WT	WT	MUT	WT	WT	WT	WT
WT	NULL	WT	MISS	WT	WT	MISS	WT	WT	WT	WT
	Q165*		Y236S			R280G				
ERpHER2n	ERnHER2n	ERpHER2n	ERnHER2n	HER2p	ERpHER2n	ERpHER2n	ERpHER2n	ERpHER2n	ERnHER2n	ERpHER2n
Living	Living	Living	Living	Died of Other Causes	Living	Died of Disease	Died of Other Causes	Died of Disease	Died of Disease	Died of Disease
91.06666667	80.23333333	95.86666667	120.4333333	146.4	299.4	18.83333333	58.13333333	153	21.7	52.3
NO	NO	NO	YES	NO	NO	YES	NO	NO	NO	NO

MB_5558	MB_4750	MB_4793	MB_4843	MB_5563	MB_5546	MB_5513	MB_5656	MB_5530	MB_5553
MUT	WT	MUT	WT	WT	WT	MUT	WT	WT	WT
MISS	WT	NULL	WT	WT	WT	NULL	WT	WT	WT
G266V		I195Tfs*52				R209Kfs*6			
HER2p	ERpHER2n	ERnHER2n	ERpHER2n	ERpHER2n	ERpHER2n	ERpHER2n	ERpHER2n	ERpHER2n	ERpHER2n
Living	Died of Disease	Died of Other Causes	Living	Died of Other Causes	Living	Died of Disease	Died of Other Causes	Living	Died of Other Causes
25.03333333	37.73333333	83.36666667	265.1333333	117.6666667	233.2666667	36.63333333	126.4	185.9333333	154.5
YES	NO	NO	NO	NO	NO	NO	NO	NO	NO

MB_4836	MB_4828	MB_4697	MB_4679	MB_4033	MB_4692	MB_4675	MB_4653	MB_4663	MB_4660
WT	WT	WT	MUT	MUT	WT	WT	MUT	MUT	MUT
WT	WT	WT	NULL	MISS	WT	WT	MISS	MISS	MISS
			Y107*	E271K			L257P	Y220C	I255F
ERpHER2n	ERpHER2n	ERpHER2n	ERnHER2n	ERpHER2n	ERpHER2n	ERpHER2n	ERpHER2n	HER2p	ERnHER2n
Died of Disease	Died of Disease	Died of Other Causes	Died of Other Causes	Died of Disease	Died of Other Causes	Living	Died of Disease	Living	Died of Disease
23.43333333	42.5	118	292.6666667	15.2	107.2666667	263.0333333	48.13333333	239.3333333	39.86666667
NO	NO	NO	NO	NO	NO	NO	NO	NO	YES

MB_5275	MB_4654	MB_2618	MB_2718	MB_2643	MB_2629	MB_2645	MB_2626	MB_6263	MB_6305	MB_6185
WT	WT	WT	MUT	WT	WT	MUT	WT	WT	MUT	WT
WT	WT	WT	NULL	WT	WT	MISS	WT	WT	NULL	WT
			R342*			R175H			X126_splice	
ERpHER2n	ERpHER2n	ERpHER2n	ERnHER2n	ERnHER2n	ERpHER2n	ERpHER2n	HER2p	ERpHER2n	ERnHER2n	ERpHER2n
Died of Other Causes	Died of Other Causes	Died of Disease	Living	Died of Disease	Living	Died of Disease	Living	Living	Died of Disease	Died of Other Causes
75.96666667	64.3	89.1	278.2666667	52.96666667	279.1	44.76666667	193.7	75.86666667	89.53333333	197.7333333
NO	NO	NO	YES	NO	NO	NO	NO	NO	YES	NO

MB_6187	MB_6248	MB_6330	MB_6184	MB_6329	MB_6283	MB_6359	MB_6337	MB_6256
WT	MUT	WT	MUT	MUT	WT	MUT	MUT	WT
WT	MISS	WT	MISS	MISS	WT	MISS	MISS	WT
	R248W		R280K	R337H		Q317R	G266V	
ERpHER2n	ERnHER2n	HER2p	ERpHER2n	ERpHER2n	ERpHER2n	ERpHER2n	HER2p	ERpHER2n
Died of Other Causes	Living	Living	Died of Disease	Died of Other Causes	Died of Other Causes	Died of Disease	Died of Other Causes	Died of Other Causes
37.76666667	182.8333333	140.3333333	185.7	81.5	92.4	85.03333333	110.8666667	127.8333333
NO	YES	YES	YES	NO	NO	NO	YES	NO

MB_6201	MB_6207	MB_6194	MB_6281	MB_6322	MB_6223	MB_6190	MB_6306	MB_6188
WT	WT	MUT	MUT	WT	MUT	WT	WT	MUT
WT	WT	MISS	NULL	WT	MISS	WT	WT	MISS
		R280K	L265Gfs*34		G245D			A159P
ERpHER2n	ERpHER2n	ERnHER2n	ERpHER2n	ERpHER2n	ERnHER2n	ERpHER2n	ERpHER2n	ERnHER2n
Died of Other Causes	Died of Other Causes	Died of Disease	Died of Other Causes	Died of Other Causes	Living	Died of Other Causes	Died of Other Causes	Living
222.7	118.3	52.46666667	3.5	80.36666667	221.9333333	24.63333333	64.6	23.76666667
NO	NO	YES	NO	NO	YES	NO	NO	NO

MB_6318	MB_6228	MB_6226	MB_6225	MB_6280	MB_6253	MB_6098	MB_6017	MB_6019	MB_6163
MUT	MUT	WT	MUT	MUT	WT	MUT	WT	MUT	WT
NULL	MISS	WT	NULL	NULL	WT	NULL	WT	MISS	WT
Q331*	R249S		I254Sfs*91	X126_splice		S215Cfs*6		R248Q	
ERnHER2n	ERnHER2n	ERpHER2n	ERpHER2n	ERnHER2n	ERpHER2n	ERnHER2n	ERpHER2n	ERpHER2n	ERpHER2n
Died of Other Causes	Living	Died of Other Causes	Living	Died of Disease	Living	Died of Disease	Died of Other Causes	Died of Other Causes	Died of Other Causes
109.2333333	200.1	35.36666667	117.8666667	27.86666667	194.3666667	10.63333333	70.9	13.53333333	88.66666667
NO	NO	NO	NO	YES	NO	YES	NO	NO	NO

MB_6157	MB_6179	MB_6105	MB_6047	MB_6001	MB_6050	MB_6169	MB_4548	MB_4564
MUT	MUT	WT	MUT	MUT	WT	MUT	MUT	WT
MISS	MISS	WT	MISS	MISS	WT	MISS	MISS	WT
R248W	G245S		C242F	F270L		H193Y	C238R	
HER2p	ERpHER2n	HER2p	ERpHER2n	ERpHER2n	ERpHER2n	HER2p	HER2p	ERpHER2n
Died of Disease	Died of Disease	Died of Other Causes	Died of Disease	Died of Other Causes	Living	Died of Other Causes	Living	Died of Other Causes
15.86666667	58.46666667	192.2	37.86666667	135.3	192.2	84.73333333	322.8333333	210.4333333
NO	NO	NO	NO	NO	NO	NO	YES	NO

MB_4591	MB_4557	MB_4601	MB_4598	MB_4578	MB_4593	MB_5186	MB_4974	MB_4978	MB_5086
WT	WT	MUT	WT	WT	WT	MUT	MUT	WT	WT
WT	WT	NULL	WT	WT	WT	NULL	NULL	WT	WT
		X261_splice				R196*	G108Vfs*15		
ERpHER2n	ERpHER2n	HER2p	ERpHER2n	ERpHER2n	ERpHER2n	HER2p	ERnHER2n	ERpHER2n	ERpHER2n
Died of Other Causes	Living	Died of Disease	Died of Disease	Died of Other Causes	Living	Living	Died of Other Causes	Living	Died of Other Causes
41.16666667	279.8	56.26666667	119.3666667	197.3333333	140.2333333	203.2666667	176.1	198.4333333	84.23333333
NO	NO	YES	NO	NO	NO	NO	NO	NO	NO

MB_5150	MB_5345	MB_4696	MB_4695	MB_4714	MB_4705	MB_4882	MB_4879	MB_4881
WT	WT	MUT	WT	MUT	WT	WT	MUT	MUT
WT	WT	NULL	WT	MISS	WT	WT	MISS	MISS
		X307_splice		R248Q			P278S	C242Y
ERpHER2n	ERpHER2n	ERnHER2n	ERpHER2n	ERnHER2n	ERpHER2n	ERpHER2n	HER2p	ERnHER2n
Died of Disease	Died of Disease	Living	Died of Other Causes	Died of Other Causes	Died of Other Causes	Died of Other Causes	Died of Other Causes	Living
110.8333333	216.0333333	255.2666667	21.3	83.66666667	213.1	266.1	79.96666667	274.2
NO	NO	NO	NO	YES	NO	NO	NO	NO

MB_4846	MB_4870	MB_4224	MB_4845	MB_4904	MB_4865	MB_4876	MB_4860	MB_5081	MB_4897	MB_4925
WT	WT	WT	WT	WT	MUT	WT	MUT	MUT	WT	WT
WT	WT	WT	WT	WT	MISS	WT	MISS	MISS	WT	WT
					R175H		R175H	V173M		
HER2p	ERpHER2n	ERpHER2n	ERpHER2n	ERnHER2n	ERnHER2n	ERpHER2n	ERpHER2n	ERpHER2n	ERpHER2n	ERpHER2n
Died of Disease	Living	Died of Disease	Died of Disease	Died of Other Causes	Living	Died of Disease	Living	Died of Disease	Living	Died of Other Causes
101.6333333	229.0666667	65.5	25.46666667	129.2333333	229.9	150.6	234.6	58	197.4333333	102.7666667
NO	NO	NO	NO	YES	NO	NO	NO	NO	NO	NO

MB_4883	MB_4896	MB_4987	MB_4416	MB_4374	MB_4407	MB_5039	MB_4119	MB_4091	MB_5079
WT	MUT	WT	MUT	WT	MUT	WT	WT	WT	WT
WT	MISS	WT	NULL	WT	MISS	WT	WT	WT	WT
	G245V		V122Cfs*27		R248Q				
ERpHER2n	HER2p	ERpHER2n	ERnHER2n	ERpHER2n	ERnHER2n	HER2p	ERpHER2n	ERpHER2n	ERpHER2n
Living	Died of Disease	Died of Other Causes	Died of Disease	Died of Other Causes	Died of Disease	Died of Disease	Died of Disease	Died of Other Causes	Living
268.9	64.86666667	221.7666667	241.6	42.56666667	83.36666667	62.13333333	67.8	281.3666667	199.9333333
NO	NO	NO	YES	NO	NO	NO	NO	NO	NO

MB_5073	MB_5105	MB_4254	MB_5174	MB_4742	MB_5239	MB_5251	MB_4996	MB_4282	MB_4603
WT	WT	MUT	MUT	WT	WT	WT	WT	MUT	WT
WT	WT	NULL	MISS	WT	WT	WT	WT	NULL	WT
		R282Gfs*63	G245V					Q317*	
ERpHER2n	ERpHER2n	ERnHER2n	HER2p	ERpHER2n	ERpHER2n	ERpHER2n	ERpHER2n	ERpHER2n	ERpHER2n
Died of Other Causes	Died of Disease	Living	Died of Other Causes	Died of Other Causes	Living	Died of Disease	Died of Other Causes	Living	Living
194.1666667	204.4333333	178.4	151.2	83.13333333	192.1333333	112.4666667	99.76666667	34.1	239.2333333
NO	NO	NO	NO	NO	NO	NO	NO	NO	NO

MB_4621	MB_4853	MB_4362	MB_4602	MB_4222	MB_4893	MB_4859	MB_4212	MB_4873	MB_4942	MB_4949
MUT	WT	WT	WT	WT	MUT	WT	WT	WT	MUT	MUT
NULL	WT	WT	WT	WT	NULL	WT	WT	WT	NULL	MISS
R213*					R213*				X126_splice	R175H
ERnHER2n	ERpHER2n	ERpHER2n	ERpHER2n	ERpHER2n	ERnHER2n	ERnHER2n	ERpHER2n	ERpHER2n	ERnHER2n	ERpHER2n
Living	Living	Living	Died of Disease	Died of Disease	Living	Died of Other Causes	Living	Died of Disease	Died of Other Causes	Living
271.8666667	265.9333333	267.2666667	122.8	55.23333333	176.7	157.8	330.3666667	117.6666667	124.1333333	216.9666667
YES	NO	NO	NO	NO	YES	NO	NO	NO	NO	NO

MB_4928	MB_5098	MB_5313	MB_4145	MB_5122	MB_5278	MB_4213	MB_4126	MB_4957
MUT	WT	MUT	MUT	WT	WT	WT	MUT	WT
NULL	WT	MISS	MISS	WT	WT	WT	MISS	WT
V157_R158del		R273G	H179Q				Y220C	
ERnHER2n	ERpHER2n	ERpHER2n	ERpHER2n	ERpHER2n	ERpHER2n	ERpHER2n	ERpHER2n	ERpHER2n
Living	Died of Other Causes	Died of Disease	Died of Other Causes	Died of Other Causes	Died of Disease	Died of Disease	Died of Other Causes	Died of Disease
220.0333333	186.4	46.53333333	217.6333333	125.7	99.96666667	84.5	174.8	197.6666667
NO	NO	NO	NO	NO	NO	NO	NO	NO

MB_5470	MB_5317	MB_5308	MB_4976	MB_5277	MB_5110	MB_4046	MB_4012	MB_4141	MB_4001
WT	WT	WT	WT	WT	WT	WT	WT	WT	WT
WT	WT	WT	WT	WT	WT	WT	WT	WT	WT
ERpHER2n	ERpHER2n	ERpHER2n	ERpHER2n	ERpHER2n	ERpHER2n	ERpHER2n	HER2p	ERpHER2n	ERpHER2n
Died of Disease	Died of Disease	Died of Disease	Died of Other Causes	Died of Disease	Died of Disease	Died of Other Causes	Died of Other Causes	Died of Disease	Died of Disease
42.06666667	53.63333333	104.7666667	224.1	221.0666667	21.9	298.0333333	277.3666667	147.8333333	49.66666667
NO	NO	NO	NO	NO	NO	NO	NO	NO	NO

MB_4792	MB_4738	MB_4264	MB_4794	MB_4782	MB_5008	MB_4426	MB_4390	MB_4434	MB_4368
MUT	WT	WT	WT	WT	MUT	MUT	WT	WT	WT
MISS	WT	WT	WT	WT	NULL	NULL	WT	WT	WT
G245C					X225_splice	P153Afs*28			
ERnHER2n	ERpHER2n	ERpHER2n	ERpHER2n	ERpHER2n	ERnHER2n	ERpHER2n	ERpHER2n	ERpHER2n	ERpHER2n
Died of Disease	Living	Died of Other Causes	Died of Other Causes	Living	Living	Died of Other Causes	Died of Other Causes	Died of Disease	Died of Disease
40.63333333	176.0666667	163.2	45.5	251.0666667	223.3	170.9	164.3333333	148.1	119.4666667
YES	NO	NO	NO	NO	YES	NO	NO	NO	NO

MB_4442	MB_4418	MB_4381	MB_4395	MB_4630	MB_6010	MB_6021	MB_6059	MB_6154	MB_6097	MB_6107
WT	WT	WT	WT	WT	MUT	WT	MUT	WT	WT	WT
WT	WT	WT	WT	WT	MISS	WT	MISS	WT	WT	WT
					R175H		R175G			
ERpHER2n	ERpHER2n	ERpHER2n	ERpHER2n	ERpHER2n	ERpHER2n	ERpHER2n	ERpHER2n	ERpHER2n	ERpHER2n	ERpHER2n
Living	Living	Died of Disease	Died of Disease	Died of Other Causes	Living	Living	Living	Living	Died of Other Causes	Died of Other Causes
296.8666667	307.6333333	61.46666667	139.6	95.83333333	218.2333333	301.2333333	278.4666667	195.3	159.7	123.3333333
NO	NO	NO	NO	NO	NO	NO	YES	NO	NO	NO

MB_6023	MB_6152	MB_6018	MB_6082	MB_6029	MB_5295	MB_5386	MB_5092	MB_6068	MB_6039
WT	MUT	WT	WT	WT	MUT	WT	WT	MUT	MUT
WT	MISS	WT	WT	WT	MISS	WT	WT	NULL	NULL
	P278S				G245D			E294*	X307_splice
ERpHER2n	ERnHER2n	ERpHER2n	HER2p	ERpHER2n	ERnHER2n	ERpHER2n	ERpHER2n	ERnHER2n	ERpHER2n
Living	Living	Died of Disease	Died of Disease	Died of Disease	Died of Other Causes	Living	Died of Other Causes	Died of Other Causes	Died of Other Causes
232.9666667	228.9	143.1666667	102.5	278.3666667	75.7	192.2	174.5666667	182.6	73.13333333
NO	NO	NO	YES	NO	NO	NO	NO	NO	NO

MB_6022	MB_6125	MB_6053	MB_6135	MB_6042	MB_6058	MB_6077	MB_6052	MB_5368	MB_4189
WT	WT	MUT	MUT	WT	MUT	WT	WT	WT	MUT
WT	WT	NULL	NULL	WT	MISS	WT	WT	WT	MISS
		C135Afs*35	K305Sfs*40		Y220C				G245S
ERpHER2n	ERpHER2n	ERpHER2n	ERpHER2n	ERpHER2n	ERnHER2n	ERpHER2n	ERnHER2n	ERpHER2n	ERpHER2n
Died of Disease	Died of Other Causes	Living	Died of Other Causes	Living	Died of Disease	Died of Other Causes	Living	Died of Other Causes	Died of Other Causes
52.06666667	153.9	250.8	78.6	263.4333333	22.66666667	170.2666667	255.3666667	100.1333333	355.2
NO	NO	NO	NO	NO	YES	NO	NO	NO	NO

MB_5335	MB_4008	MB_5134	MB_4139	MB_5130	MB_5135	MB_4880	MB_5054	MB_5057	MB_4098	MB_4760	MB_5102
MUT	WT	WT	WT	MUT	MUT	MUT	MUT	MUT	WT	WT	MUT
NULL	WT	WT	WT	MISS	NULL	NULL	MISS	NULL	WT	WT	MISS
T125Vfs*23				R280T	G108Vfs*13	Y107*	R248Q	N239*			Y220C
ERnHER2n	ERpHER2n	ERpHER2n	ERpHER2n	ERpHER2n	ERnHER2n	ERnHER2n	HER2p	ERnHER2n	ERpHER2n	ERpHER2n	ERnHER2n
Died of Disease	Died of Disease	Living	Died of Other Causes	NA	Died of Disease	Living	Living	Died of Disease	Living	Died of Disease	Died of Disease
25.23333333	44.73333333	196.5333333	165.4	255	37.36666667	210.9666667	187.7	56.33333333	176.3666667	124.8	30.36666667
NO	NO	NO	NO	NO	NO	NO	NO	NO	NO	NO	YES

MB_5420	MB_5458	MB_5492	MB_5407	MB_5394	MB_5410	MB_5435	MB_5406	MB_4329	MB_4005	MB_6288	MB_6272
WT	MUT	WT	WT	WT	WT	WT	WT	WT	MUT	MUT	MUT
WT	MISS	WT	WT	WT	WT	WT	WT	WT	MISS	NULL	NULL
	E285K								R175H	E198*	T125T
HER2p	HER2p	ERpHER2n	ERpHER2n	HER2p	ERpHER2n	ERpHER2n	ERpHER2n	ERpHER2n	ERpHER2n	ERpHER2n	ERnHER2n
Living	Living	Died of Disease	Living	Living	Living	Died of Other Causes	Died of Disease	Died of Disease	Living	Died of Disease	Died of Disease
193.9	183.4333333	23.36666667	205.6	193.6	202.7666667	104	97.43333333	168.2666667	188.3666667	100.3	22.4
NO	YES	NO	NO	NO	NO	NO	NO	NO	NO	NO	YES

MB_6218	MB_6246	MB_6217	MB_6308	MB_6271	MB_6245	MB_6233	MB_4343	MB_4281	MB_4283
WT	MUT	WT	WT	MUT	WT	WT	MUT	MUT	WT
WT	NULL	WT	WT	MISS	WT	WT	NULL	MISS	WT
	P322Hfs*23			C135F			R342*	L194R	
ERpHER2n	HER2p	ERpHER2n	ERpHER2n	ERpHER2n	ERnHER2n	ERpHER2n	ERpHER2n	ERpHER2n	ERpHER2n
Died of Disease	Died of Other Causes	Died of Other Causes	Died of Other Causes	Living	Living	Living	Died of Other Causes	Died of Other Causes	Living
147.7666667	52.9	211.5333333	151.9333333	157.7333333	177.6333333	201.1666667	198.6	260.0333333	174.8333333
NO	YES	YES	NO	NO	NO	NO	NO	NO	NO

MB_4341	MB_4303	MB_4274	MB_4292	MB_4278	MB_5399	MB_5364	MB_3526	MB_6138	MB_6049
MUT	MUT	WT	WT	WT	WT	WT	MUT	WT	MUT
NULL	MISS	WT	WT	WT	WT	WT	MISS	WT	NULL
W146*	D281A						R282W		R196*
HER2p	ERnHER2n	ERpHER2n	ERpHER2n	ERpHER2n	ERpHER2n	ERpHER2n	ERpHER2n	ERpHER2n	HER2p
Died of Disease	Died of Other Causes	Died of Disease	Died of Other Causes	Died of Other Causes	Died of Disease	Died of Disease	Died of Disease	Died of Disease	Living
33.7	89.03333333	59.93333333	335.6	244.2	106.8	125.7666667	86.06666667	25.63333333	264.2333333
YES	NO	NO	NO	NO	NO	NO	NO	NO	NO

MB_6055	MB_6108	MB_6044	MB_6181	MB_6100	MB_6085	MB_6113	MB_6079	MB_6103	MB_6016
MUT	WT	MUT	WT	MUT	WT	MUT	WT	WT	WT
MISS	WT	MISS	WT	NULL	WT	MISS	WT	WT	WT
R248W		G334V		G302Rfs*4		D259Y			
ERnHER2n	ERpHER2n	ERpHER2n	ERpHER2n	HER2p	HER2p	HER2p	ERpHER2n	ERpHER2n	ERpHER2n
Living	Living	Died of Other Causes	Died of Other Causes	Died of Disease	Living	Died of Disease	Died of Disease	Living	Died of Other Causes
200.4333333	193.9666667	219.1	54.93333333	10.06666667	23.9	124.5666667	239.1666667	239.4	143.5333333
YES	NO	NO	NO	YES	YES	YES	NO	NO	NO

MB_6149	MB_6131	MB_6007	MB_6147	MB_6114	MB_6167	MB_6146	MB_6092	MB_6160	MB_6075
WT	MUT	MUT	WT	MUT	WT	MUT	WT	MUT	WT
WT	NULL	MISS	WT	MISS	WT	NULL	WT	MISS	WT
	X224_splice	R175H		Y234C		R158Pfs*12		R248G	
ERpHER2n	HER2p	ERpHER2n	ERpHER2n	HER2p	ERpHER2n	ERpHER2n	ERpHER2n	HER2p	ERpHER2n
Died of Other Causes	Living	Died of Other Causes	Died of Other Causes	Living	Died of Other Causes	Died of Disease	Died of Other Causes	Living	Died of Disease
74.93333333	257.6666667	70.53333333	238.0666667	66.83333333	122.8	89.6	2.3	247.3666667	182.3333333
NO	NO	NO	NO	YES	NO	NO	NO	YES	NO

MB_6011	MB_6071	MB_6214	MB_6334	MB_6346	MB_6319	MB_6200	MB_6336	MB_6211	MB_6232	MB_6234
WT	MUT	WT	MUT	WT	WT	MUT	MUT	WT	WT	WT
WT	MISS	WT	NULL	WT	WT	NULL	MISS	WT	WT	WT
	R110H		E198*			R213*	R273H			
ERpHER2n	ERpHER2n	ERpHER2n	HER2p	ERpHER2n	ERpHER2n	ERpHER2n	ERnHER2n	ERpHER2n	ERpHER2n	ERpHER2n
Died of Disease	Died of Disease	Died of Other Causes	Living	Living	Living	Died of Disease	Died of Disease	Died of Other Causes	Died of Other Causes	Living
211.1333333	57.4	174.5	128.1	281.5	154.7	30.06666667	17.2	42.3	85	213.3666667
NO	NO	NO	NO	NO	YES	NO	YES	NO	NO	NO

MB_6300	MB_5533	MB_5543	MB_4236	MB_4234	MB_4270	MB_4235	MB_4250	MB_5467
WT	MUT	MUT	WT	WT	MUT	WT	MUT	WT
WT	NULL	NULL	WT	WT	MISS	WT	MISS	WT
	E171*	Y107_F109del			R175H		R175H	
ERpHER2n	ERpHER2n	ERpHER2n	ERpHER2n	ERpHER2n	HER2p	ERpHER2n	ERpHER2n	ERpHER2n
Died of Other Causes	Living	Died of Other Causes	Died of Other Causes	Died of Other Causes	Died of Disease	Died of Disease	Died of Other Causes	Living
63.83333333	158.0333333	34.43333333	203.7	253.5333333	98.7	335.7333333	100.1666667	186.1
NO	NO	NO	NO	NO	YES	NO	NO	NO

MB_4323	MB_4318	MB_4333	MB_4339	MB_4322	MB_4351	MB_5063	MB_4360	MB_4375	MB_4798
WT	WT	MUT	WT	WT	MUT	MUT	MUT	WT	WT
WT	WT	MISS	WT	WT	MISS	MISS	MISS	WT	WT
		R175H			V157F	Y163C	G262V		
ERpHER2n	ERpHER2n	ERpHER2n	ERpHER2n	ERpHER2n	ERnHER2n	HER2p	HER2p	ERpHER2n	ERpHER2n
Died of Other Causes	Died of Other Causes	Died of Other Causes	Died of Disease	Died of Other Causes	Died of Disease	Living	Died of Disease	Living	Living
126.6333333	17.13333333	207.9666667	173.6	196.4666667	68.13333333	215.1666667	11.86666667	291.1666667	257.1666667
NO	NO	NO	NO	NO	NO	YES	YES	NO	NO

MB_4746	MB_4838	MB_4829	MB_4802	MB_5426	MB_5444	MB_5431	MB_5447	MB_5434	MB_5231	MB_5339
WT	MUT	WT	WT	MUT	WT	MUT	WT	WT	MUT	WT
WT	MISS	WT	WT	NULL	WT	NULL	WT	WT	NULL	WT
	C238S			X187_splice		A83Pfs*35			S149Pfs*21	
ERpHER2n	ERpHER2n	ERpHER2n	ERpHER2n	HER2p	ERpHER2n	ERpHER2n	ERpHER2n	ERpHER2n	HER2p	ERpHER2n
Living	Died of Other Causes	Died of Other Causes	Died of Disease	Died of Disease	Living	Died of Disease	Died of Disease	Died of Disease	Died of Disease	Living
244.4333333	200.7666667	176.2666667	43.9	34.63333333	240.4333333	15.86666667	148.8666667	45.16666667	16.56666667	195.8666667
YES	NO	NO	NO	YES	NO	NO	NO	NO	NO	NO

MB_5208	MB_5428	MB_5440	MB_5425	MB_5298	MB_5334	MB_5326	MB_4059	MB_6230	MB_6150
MUT	WT	MUT	WT	MUT	WT	WT	WT	WT	WT
MISS	WT	MISS	WT	MISS	WT	WT	WT	WT	WT
S127F		N239D		M246R					
ERnHER2n	ERpHER2n	ERnHER2n	ERpHER2n	ERnHER2n	ERpHER2n	ERpHER2n	ERpHER2n	ERpHER2n	ERpHER2n
Living	Living	Living	Died of Disease	Died of Disease	Died of Other Causes	Died of Other Causes	Died of Other Causes	Died of Disease	Died of Other Causes
151.9	150.4666667	196.6333333	49.46666667	84.9	85.4	96.2	172	74.8	77.66666667
NO	NO	YES	NO	YES	NO	NO	NO	NO	NO

MB_6014	MB_6145	MB_6012	MB_6024	MB_6124	MB_6171	MB_6060	MB_6008	MB_6036	MB_6144
WT	WT	WT	WT	WT	WT	WT	WT	MUT	MUT
WT	WT	WT	WT	WT	WT	WT	WT	MISS	MISS
								R175H	R175H
ERpHER2n	ERpHER2n	ERpHER2n	ERpHER2n	ERpHER2n	ERpHER2n	ERpHER2n	ERpHER2n	HER2p	ERnHER2n
Died of Other Causes	Died of Disease	Died of Other Causes	Died of Disease	Died of Other Causes	Living	Died of Other Causes	Died of Disease	Living	Living
191.9333333	34.33333333	144.4333333	252.2666667	115.6333333	220.3	38.03333333	69.1	287.9333333	254.9333333
NO	NO	NO	NO	NO	NO	NO	NO	NO	NO

MB_5346	MB_5373	MB_6178	MB_6063	MB_6062	MB_6069	MB_6141	MB_6118	MB_6065	MB_6006
MUT	WT	MUT	WT	MUT	WT	WT	WT	WT	WT
MISS	WT	NULL	WT	MISS	WT	WT	WT	WT	WT
V173G		E51Lfs*73		E285K					
ERnHER2n	ERpHER2n	ERnHER2n	HER2p	ERnHER2n	ERpHER2n	ERpHER2n	ERpHER2n	ERpHER2n	ERpHER2n
Living	Living	Died of Disease	Died of Disease	Living	Died of Other Causes	Died of Other Causes	Living	Died of Other Causes	Died of Other Causes
194.3	2.533333333	33.13333333	15.6	282.3666667	258.1333333	78.86666667	264.6	188.5333333	63.56666667
YES	NO	NO	YES	NO	NO	NO	NO	NO	NO

MB_6048	MB_6116	MB_4310	MB_4300	MB_4417	MB_4079	MB_4317	MB_4350	MB_4332	MB_4324
MUT	MUT	WT	MUT	MUT	MUT	MUT	MUT	MUT	WT
NULL	NULL	WT	NULL	MISS	MISS	MISS	NULL	MISS	WT
C124Afs*46	E62*		R209Kfs*6	R280T	V274L	A159V	R306*	K132N	
HER2p	HER2p	ERpHER2n	ERpHER2n	ERnHER2n	ERpHER2n	HER2p	HER2p	ERnHER2n	ERpHER2n
Died of Other Causes	Living	Died of Other Causes	Living	Died of Other Causes	Died of Other Causes	Living	Died of Disease	Living	Died of Disease
168.2	193.1333333	265.5666667	189.0333333	16.7	351	177.9	31.06666667	307.9333333	87.23333333
NO	NO	NO	NO	NO	NO	NO	YES	NO	NO

MB_4306	MB_7226	MB_7205	MB_7160	MB_7159	MB_7167	MB_7207	MB_7237	MB_7241	MB_7236	MB_7168	MB_7162
WT	WT	MUT	MUT	MUT	WT	MUT	WT	WT	WT	WT	WT
WT	WT	MISS	MISS	NULL	WT	MISS	WT	WT	WT	WT	WT
		R273H	H179D	T102Ifs*46		P250L					
ERpHER2n	ERpHER2n	ERnHER2n	ERpHER2n	ERnHER2n	ERpHER2n	HER2p	ERpHER2n	ERpHER2n	ERpHER2n	ERpHER2n	ERpHER2n
Died of Other Causes	Died of Disease	Living	Living	Died of Disease	Living	Died of Disease	Died of Disease	Living	Died of Other Causes	Living	Living
234.7	53.36666667	166.0333333	148.0666667	21.93333333	141.0333333	55.73333333	23.93333333	171.1	201.4666667	150.5	140.2
NO	YES	YES	NO	YES	NO	YES	NO	NO	NO	NO	NO

MB_7235	MB_4331	MB_4353	MB_4354	MB_4328	MB_4348	MB_6030	MB_6133	MB_6083
WT	WT	WT	WT	MUT	WT	WT	WT	WT
WT	WT	WT	WT	MISS	WT	WT	WT	WT
				P151T				
ERpHER2n	ERpHER2n	ERpHER2n	ERnHER2n	ERpHER2n	HER2p	ERpHER2n	ERpHER2n	ERpHER2n
Living	Died of Other Causes	Died of Disease	Died of Disease	Died of Other Causes	Died of Disease	Died of Other Causes	Died of Disease	Died of Other Causes
170.8	235.5666667	128.5333333	15.36666667	199.2666667	226.2	145.3666667	61.8	164.0333333
NO	NO	NO	NO	NO	NO	NO	NO	NO

MB_6026	MB_6168	MB_6101	MB_6156	MB_6051	MB_6182	MB_6080	MB_6143	MB_6164	MB_0551	MB_0391
MUT	WT	WT	MUT	WT	MUT	MUT	MUT	WT	WT	WT
MISS	WT	WT	MISS	WT	MISS	MISS	MISS	WT	WT	WT
C176S			F134C		C238Y	R175H	R248Q			
ERpHER2n	ERpHER2n	ERpHER2n	HER2p	ERpHER2n	HER2p	ERpHER2n	ERnHER2n	ERpHER2n	ERpHER2n	HER2p
Died of Other Causes	Died of Other Causes	Died of Other Causes	Died of Disease	Died of Disease	Living	Died of Disease	Living	Living	Died of Disease	Living
49.73333333	225.7	77.5	58.93333333	186.8333333	208.8	54.1	255.6	205.9	51.96666667	142.4666667
NO	NO	NO	NO	NO	NO	NO	NO	NO	NO	YES

MB_0367	MB_5171	MB_5199	MB_5177	MB_5178	MB_5280	MB_5148	MB_4342	MB_5225	MB_4024	MB_6314
WT	WT	MUT	WT	WT	WT	WT	WT	MUT	MUT	WT
WT	WT	MISS	WT	WT	WT	WT	WT	NULL	MISS	WT
		V173L						Q52Pfs*5	G244C	
ERpHER2n	ERpHER2n	HER2p	ERpHER2n	ERpHER2n	ERpHER2n	ERnHER2n	ERpHER2n	ERnHER2n	ERnHER2n	HER2p
Living	Died of Disease	Living	Died of Other Causes	Living	Died of Disease	Died of Other Causes	Died of Other Causes	Died of Disease	Living	Living
142.4333333	185	204.4	65.23333333	184.3333333	77.86666667	245.2333333	232.7666667	43.26666667	124.1	134.8333333
NO	NO	NO	NO	NO	NO	NO	NO	NO	NO	NO

MB_6257	MB_6358	MB_6242	MB_6237	MB_6183	MB_6254	MB_6231	MB_6204	MB_6229	MB_5154
WT	MUT	MUT	MUT	WT	WT	WT	MUT	WT	MUT
WT	NULL	NULL	MISS	WT	WT	WT	MISS	WT	MISS
	N345_L348delinsM	X225_splice	R175H				H179R		G245C
ERpHER2n	HER2p	ERnHER2n	ERnHER2n	ERpHER2n	ERpHER2n	ERpHER2n	ERpHER2n	ERpHER2n	HER2p
Living	Died of Disease	Living	Died of Other Causes	Died of Other Causes	Died of Disease	Died of Other Causes	Died of Other Causes	Died of Disease	Living
91	37.86666667	165.4333333	105.2	79.13333333	60.9	72.56666667	56.76666667	0.1	170.8333333
NO	NO	YES	NO	NO	NO	NO	NO	NO	NO

MB_5175	MB_5173	MB_5162	MB_5164	MB_7298	MB_7299	MB_7293	MB_3978	MB_7288	MB_2923	MB_0402
MUT	MUT	WT	WT	WT	WT	MUT	MUT	WT	WT	WT
MISS	MISS	WT	WT	WT	WT	MISS	NULL	WT	WT	WT
R248Q	R248W					I255S	N200Ifs*47			
ERpHER2n	ERnHER2n	ERnHER2n	HER2p	ERpHER2n	ERpHER2n	ERpHER2n	HER2p	ERpHER2n	HER2p	ERpHER2n
Died of Other Causes	Died of Disease	Living	Living	Died of Other Causes	Died of Other Causes	Living	Died of Disease	Died of Disease	Died of Disease	Living
194.7	22.13333333	164.3333333	80.5	86.23333333	201.9	199.2333333	100.8666667	27.06666667	14.1	99.73333333
NO	NO	NO	NO	NO	NO	NO	NO	NO	YES	NO

MB_0381	MB_0592	MB_5271	MB_5235	MB_4931	MB_5332	MB_7045	MB_7049	MB_7031	MB_7061	MB_7050	MB_7078
MUT	WT	MUT	MUT	MUT	MUT	MUT	WT	MUT	WT	MUT	WT
MISS	WT	NULL	MISS	NULL	NULL	NULL	WT	MISS	WT	NULL	WT
D208V		P34Vfs*12	C141Y	X10_splice	E204*	R306*		V173L		Q167*	
HER2p	ERpHER2n	ERpHER2n	ERnHER2n	ERnHER2n	HER2p	ERnHER2n	ERnHER2n	ERnHER2n	ERpHER2n	HER2p	ERnHER2n
Living	Living	Living	Died of Disease	Died of Disease	Died of Disease	Died of Disease	Living	Living	Died of Other Causes	Died of Disease	Died of Disease
130.2	74.56666667	167.5	204.2	30.36666667	98.83333333	94.93333333	102.0333333	85.36666667	96.76666667	65.13333333	4.166666667
YES	NO	NO	NO	NO	NO	NO	NO	YES	NO	YES	NO

MB_7054	MB_7048	MB_7058	MB_7056	MB_7038	MB_7086	MB_0177	MB_0435	MB_0399	MB_0388	MB_0351
MUT	MUT	WT	WT	WT	WT	WT	WT	WT	WT	WT
MISS	MISS	WT	WT	WT	WT	WT	WT	WT	WT	WT
I195T	R110L									
ERnHER2n	ERpHER2n	ERpHER2n	ERpHER2n	ERnHER2n	ERpHER2n	ERpHER2n	ERnHER2n	ERnHER2n	HER2p	ERpHER2n
Died of Disease	Living	Living	Living	Living	Living	Died of Other Causes	Living	Died of Other Causes	Living	Died of Other Causes
30.43333333	88.46666667	122.5	117	81.03333333	123.5333333	45.6	110.5333333	135.3333333	97.56666667	195.3333333
YES	NO	NO	NO	YES	NO	NO	NO	YES	YES	NO

MB_0615	MB_0265	MB_0116	MB_0472	MB_0191	MB_0231	MB_0393	MB_0553	MB_0587	MB_0611	MB_0640	MB_0114
MUT	MUT	WT	WT	MUT	WT	WT	WT	MUT	MUT	WT	WT
MISS	MISS	WT	WT	MISS	WT	WT	WT	NULL	MISS	WT	WT
Y234C	S241Y			H193R				R196*	R273C		
HER2p	ERnHER2n	ERpHER2n	ERpHER2n	ERnHER2n	ERpHER2n	ERpHER2n	HER2p	ERpHER2n	ERpHER2n	ERpHER2n	ERpHER2n
Living	Living	Living	Living	Died of Other Causes	Living	Died of Disease	Died of Other Causes	Living	Living	Living	Living
34.76666667	189.1333333	122.2666667	27.4	15.3	19.6	20.83333333	85.73333333	91.26666667	104.5333333	61.33333333	13.4
NO	NO	YES	NO	NO	NO	YES	NO	YES	NO	NO	NO

MB_0222	MB_0284	MB_0459	MB_0128	MB_0639	MB_0318	MB_0417	MB_6238	MB_6286	MB_6328	MB_6284
WT	MUT	MUT	WT	MUT	WT	WT	WT	MUT	WT	WT
WT	MISS	MISS	WT	MISS	WT	WT	WT	MISS	WT	WT
	P250L	Y163C		R248Q				G245C		
ERpHER2n	ERnHER2n	ERpHER2n	ERpHER2n	ERnHER2n	ERnHER2n	ERpHER2n	ERpHER2n	ERpHER2n	ERpHER2n	ERpHER2n
Living	Living	Died of Other Causes	Living	Living	Living	Died of Disease	Died of Disease	Died of Other Causes	Living	Died of Other Causes
212.7	0	35.6	137.8	75.4	168.7	6.266666667	172.9666667	49.53333333	126.6666667	88.33333333
YES	NO	NO	NO	YES	NO	NO	NO	NO	NO	NO

MB_6363	MB_6224	MB_6297	MB_6239	MB_6208	MB_6273	MB_6344	MB_6312	MB_6212	MB_6302	MB_6327	MB_6287
WT	MUT	WT	WT	WT	MUT	MUT	WT	WT	WT	WT	WT
WT	NULL	WT	WT	WT	NULL	NULL	WT	WT	WT	WT	WT
	A84_S90del				W91*	R306*					
HER2p	HER2p	ERpHER2n	ERpHER2n	ERpHER2n	ERpHER2n	ERpHER2n	ERpHER2n	ERpHER2n	ERpHER2n	ERpHER2n	ERpHER2n
Died of Disease	Died of Disease	Living	Living	Living	Living	Died of Disease	Living	Living	Died of Other Causes	Died of Disease	Died of Disease
16.7	35.46666667	170.8666667	200.3333333	221.2	189.4333333	152.9333333	162.7666667	224.2333333	125.8666667	18.26666667	35.2
YES	YES	NO	YES	NO	YES	NO	NO	NO	NO	NO	YES

MB_6251	MB_0489	MB_0342	MB_7289	MB_3253	MB_7291	MB_7243	MB_3466	MB_7286	MB_7292
MUT	MUT	WT	WT	MUT	MUT	WT	WT	WT	WT
NULL	MISS	WT	WT	MISS	MISS	WT	WT	WT	WT
R213*	L111P			V272M	R273H				
ERnHER2n	ERnHER2n	ERpHER2n	ERpHER2n	ERpHER2n	HER2p	ERpHER2n	ERpHER2n	ERpHER2n	ERpHER2n
Died of Disease	Living	Died of Disease	Died of Disease	Died of Disease	Died of Disease	Died of Other Causes	Died of Other Causes	Died of Other Causes	Died of Disease
14.7	90.56666667	125.3333333	126.6666667	55.83333333	6.833333333	148.8	234.1333333	158.6333333	78.46666667
YES	YES	NO	NO	NO	NO	NO	NO	NO	NO

MB_7275	MB_7249	MB_7295	MB_7297	MB_7294	MB_7296	MB_3402	MB_5113	MB_5450	MB_7032	MB_7037	MB_7040
MUT	WT	WT	WT	MUT	MUT	WT	WT	MUT	WT	WT	WT
MISS	WT	WT	WT	NULL	MISS	WT	WT	MISS	WT	WT	WT
E171G				X331_splice	M237I			C141Y			
HER2p	ERpHER2n	ERpHER2n	ERpHER2n	ERpHER2n	HER2p	ERpHER2n	ERpHER2n	ERnHER2n	ERpHER2n	ERpHER2n	ERpHER2n
Died of Disease	Died of Other Causes	Living	Died of Disease	Died of Disease	Died of Disease	Died of Disease	Living	Living	Living	Living	Living
44.4	58.66666667	196.8666667	175.9666667	82.73333333	44.73333333	50.03333333	175.6333333	190.2	71.46666667	86.4	71.83333333
YES	NO	NO	NO	NO	NO	YES	NO	NO	NO	NO	NO

MB_7020	MB_7042	MB_7043	MB_7028	MB_7029	MB_7027	MB_7060	MB_7034	MB_5191	MB_5214	MB_5204
MUT	WT	WT	WT	MUT	MUT	WT	WT	WT	WT	WT
NULL	WT	WT	WT	NULL	MISS	WT	WT	WT	WT	WT
E349_G356del				Q331*	R342P					
HER2p	ERpHER2n	ERpHER2n	ERpHER2n	ERpHER2n	HER2p	ERpHER2n	ERpHER2n	ERpHER2n	ERpHER2n	ERpHER2n
Died of Disease	Living	Living	Living	Living	Died of Disease	Died of Other Causes	Living	Died of Other Causes	Died of Other Causes	Died of Other Causes
71.63333333	88.5	91.53333333	89.8	76.86666667	36.96666667	112.6666667	75.1	142.1666667	107.3666667	191.9333333
YES	NO	NO	NO	NO	YES	NO	NO	NO	NO	NO

MB_5155	MB_5190	MB_5614	MB_5592	MB_0437	MB_0028	MB_0309	MB_0100	MB_5648	MB_5572
WT	MUT	MUT	WT	WT	MUT	WT	MUT	WT	WT
WT	MISS	NULL	WT	WT	MISS	WT	MISS	WT	WT
	R273C	X187_splice			C242R		G245S		
ERnHER2n	HER2p	ERpHER2n	ERpHER2n	ERpHER2n	ERpHER2n	ERpHER2n	ERnHER2n	ERpHER2n	ERnHER2n
Living	Living	Died of Disease	Died of Other Causes	Living	Died of Other Causes	Died of Other Causes	Died of Disease	Died of Other Causes	Living
259.9333333	242.5666667	116.4333333	26.33333333	90.56666667	36.56666667	42.33333333	8.066666667	199.9666667	178.6333333
YES	NO	NO	NO	NO	NO	NO	YES	NO	NO

MB_5655	MB_5577	MB_5585	MB_5620	MB_5652	MB_0537	MB_0544	MB_0468	MB_0333	MB_0205
MUT	MUT	WT	WT	MUT	WT	WT	WT	MUT	WT
NULL	MISS	WT	WT	MISS	WT	WT	WT	MISS	WT
I255del	V216M			P190T				p.R273C	
ERnHER2n	ERnHER2n	ERpHER2n	ERpHER2n	ERpHER2n	ERpHER2n	ERpHER2n	ERpHER2n	ERnHER2n	ERpHER2n
Living	Died of Disease	Died of Disease	Died of Disease	Died of Other Causes	Living	Died of Other Causes	Died of Other Causes	Died of Disease	Living
191.8	55.4	53.9	42.06666667	104.6666667	113.5666667	79.33333333	129.4333333	26.56666667	144.4666667
NO	NO	NO	NO	NO	YES	YES	YES	YES	NO

MB_0233	MB_0210	MB_0200	MB_0244	MB_5588	MB_5628	MB_5633	MB_0169	MB_0253	MB_0226	MB_0278
MUT	WT	MUT	WT	MUT	MUT	WT	WT	WT	WT	MUT
MISS	WT	NULL	WT	MISS	NULL	WT	WT	WT	WT	MISS
R280S		R196*		R248W	P153Afs*28					R248Q
ERpHER2n	ERnHER2n	ERnHER2n	ERpHER2n	ERpHER2n	HER2p	ERnHER2n	ERpHER2n	ERpHER2n	ERpHER2n	ERnHER2n
Died of Other Causes	Living	Died of Disease	Living	Died of Disease	Died of Disease	Died of Disease	Died of Other Causes	Living	Living	Died of Disease
72.43333333	144.9333333	128.7	149.7333333	81.13333333	42.6	35.03333333	145.5	152.3	57.63333333	5.833333333
NO	NO	NO	NO	NO	NO	YES	NO	NO	NO	YES

MB_0266	MB_0577	MB_0531	MB_0245	MB_5641	MB_5640	MB_0534	MB_0230	MB_0109	MB_0304	MB_0369	MB_0627
WT	WT	WT	WT	WT	WT	WT	MUT	MUT	MUT	WT	MUT
WT	WT	WT	WT	WT	WT	WT	NULL	MISS	MISS	WT	MISS
							I255del	C277F	H193L		R280T
ERpHER2n	ERpHER2n	ERpHER2n	ERpHER2n	ERpHER2n	ERpHER2n	ERpHER2n	HER2p	ERpHER2n	ERpHER2n	ERpHER2n	ERnHER2n
Died of Other Causes	Living	Living	Living	Living	Living	Living	Living	Died of Other Causes	Died of Disease	Living	Living
90.66666667	65.4	163.8666667	164.7	181.8666667	172.3	124.1	200.3333333	112.4	111.5333333	144.3333333	0.766666667
NO	NO	YES	NO	NO	NO	YES	NO	NO	NO	YES	NO

MB_0492	MB_0147	MB_0442	MB_0111	MB_0634	MB_0280	MB_0490	MB_0178	MB_0242	MB_5624	MB_5593
WT	WT	WT	WT	WT	WT	WT	MUT	WT	MUT	MUT
WT	WT	WT	WT	WT	WT	WT	NULL	WT	NULL	NULL
							X225_splice		L45Cfs*78	-125fs
ERpHER2n	ERpHER2n	ERpHER2n	ERpHER2n	ERnHER2n	ERpHER2n	ERpHER2n	ERpHER2n	ERpHER2n	ERnHER2n	HER2p
Died of Other Causes	Died of Disease	Died of Disease	Living	Living	Died of Disease	Living	Died of Disease	Living	Living	Died of Disease
81.13333333	51.76666667	103.4666667	127.1	17.76666667	172.9	101.9666667	104.4666667	199.1	136	92.5
NO	NO	NO	NO	NO	NO	NO	NO	NO	YES	YES

MB_4010	MB_5605	MB_5636	MB_4529	MB_5566	MB_0268	MB_0275	MB_0127	MB_0045	MB_0414	MB_0259
WT	WT	WT	WT	MUT	MUT	WT	MUT	WT	MUT	MUT
WT	WT	WT	WT	NULL	NULL	WT	NULL	WT	NULL	MISS
				R282Pfs*62	R306*		Q317Sfs*28		E171Gfs*3	R273H
ERpHER2n	ERpHER2n	ERpHER2n	ERpHER2n	ERnHER2n	ERpHER2n	ERpHER2n	ERnHER2n	ERnHER2n	ERnHER2n	ERnHER2n
Died of Other Causes	Living	Died of Other Causes	Died of Other Causes	Living	Died of Disease	Living	Living	Living	Living	Died of Disease
11.7	123.7	108.3	126.4666667	234.4	28.5	186.1	132.0666667	164.9	76.63333333	11.86666667
NO	NO	NO	NO	YES	NO	NO	YES	YES	NO	YES

MB_0396	MB_0874	MB_0179	MB_0526	MB_0586	MB_0508	MB_0288	MB_0269	MB_0582	MB_0901	MB_0000
MUT	MUT	WT	WT	WT	WT	WT	WT	MUT	MUT	WT
MISS	MISS	WT	WT	WT	WT	WT	WT	MISS	NULL	WT
H179R	R280T							P151H	N131Cfs*27	
ERnHER2n	ERnHER2n	ERnHER2n	ERpHER2n	ERpHER2n	ERpHER2n	HER2p	ERnHER2n	ERnHER2n	ERnHER2n	ERpHER2n
Living	Died of Disease	Died of Disease	Died of Other Causes	Died of Other Causes	Living	Died of Disease	Living	Died of Other Causes	Living	Living
60.66666667	16.6	17.93333333	139.6333333	77.23333333	114.3333333	63.8	22.23333333	15.53333333	136.1666667	140.5
YES	YES	YES	NO	NO	NO	YES	YES	NO	YES	NO

MB_0535	MB_0469	MB_0478	MB_0499	MB_0170	MB_0464	MB_0421	MB_0623	MB_0241	MB_0163	MB_0149
WT	MUT	WT	WT	WT	MUT	MUT	WT	MUT	MUT	MUT
WT	MISS	WT	WT	WT	MISS	NULL	WT	MISS	NULL	MISS
	R175H				E285K	L194_I195insSIL		A159V	S106Rfs*41	R175H
ERpHER2n	ERpHER2n	ERpHER2n	ERnHER2n	ERpHER2n	ERnHER2n	HER2p	ERpHER2n	ERpHER2n	ERnHER2n	ERnHER2n
Living	Living	Living	Living	Died of Other Causes	Died of Disease	Died of Other Causes	Living	Died of Disease	Died of Other Causes	Died of Other Causes
199.1333333	131.2666667	132.3	131.9	93.36666667	39.3	92.86666667	111.0666667	73.13333333	98.1	51.7
NO	YES	NO	NO	NO	YES	NO	NO	NO	NO	NO

MB_0350	MB_0512	MB_0020	MB_0046	MB_0134	MB_0495	MB_0470	MB_0872	MB_0661	MB_0339
MUT	WT	WT	MUT	WT	MUT	WT	WT	WT	WT
MISS	WT	WT	MISS	WT	MISS	WT	WT	WT	WT
R248G			C135R		G245S				
ERnHER2n	ERpHER2n	ERnHER2n	HER2p	ERpHER2n	ERnHER2n	ERnHER2n	ERpHER2n	ERpHER2n	ERpHER2n
Died of Disease	Living	Died of Disease	Died of Other Causes	Died of Disease	Died of Disease	Living	Died of Other Causes	Died of Disease	Died of Other Causes
46.06666667	121.5333333	22.4	14.13333333	12.93333333	71.8	88.23333333	152.0666667	20	26.73333333
NO	NO	YES	NO	NO	YES	NO	NO	NO	NO

MB_0264	MB_0175	MB_0303	MB_0172	MB_0125	MB_0340	MB_0234	MB_0487	MB_0305	MB_0502	MB_0663	MB_0130
WT	WT	WT	MUT	WT	MUT	WT	WT	WT	WT	MUT	WT
WT	WT	WT	MISS	WT	NULL	WT	WT	WT	WT	NULL	WT
			R110P		X261_splice					C238*	
ERpHER2n	ERpHER2n	ERnHER2n	ERpHER2n	ERpHER2n	ERnHER2n	ERpHER2n	ERpHER2n	ERpHER2n	ERnHER2n	HER2p	HER2p
Died of Disease	Living	Living	Living	Living	Living	Died of Other Causes	Living	Died of Disease	Living	Living	Living
43.1	72.36666667	60.13333333	138.1	1.266666667	164.7333333	94.23333333	86.9	63.5	82.1	55.36666667	153.5666667
NO	NO	NO	YES	NO	NO	NO	NO	YES	YES	YES	NO

MB_0289	MB_0129	MB_0343	MB_0458	MB_0570	MB_0488	MB_0655	MB_0235	MB_0484	MB_0436	MB_0877
WT	WT	WT	WT	MUT	WT	WT	WT	MUT	MUT	MUT
WT	WT	WT	WT	NULL	WT	WT	WT	MISS	MISS	MISS
				Q192*				R248G	R273H	R248Q
ERnHER2n	HER2p	ERpHER2n	ERpHER2n	ERpHER2n	ERpHER2n	ERpHER2n	ERpHER2n	HER2p	ERnHER2n	ERpHER2n
Died of Other Causes	Died of Other Causes	Died of Disease	Living	Living	Living	Living	Died of Disease	Living	Died of Other Causes	Died of Disease
71.6	38.56666667	91.13333333	134.5	272.2	112.9333333	111.0666667	142.5666667	82.63333333	75.5	14.43333333
NO	YES	NO	YES	NO	NO	NO	YES	YES	NO	NO

MB_0097	MB_0897	MB_0095	MB_0227	MB_0353	MB_0522	MB_0543	MB_0646	MB_0293	MB_0552	MB_0260
WT	WT	WT	WT	MUT	MUT	WT	WT	WT	WT	WT
WT	WT	WT	WT	MISS	MISS	WT	WT	WT	WT	WT
				Q136H	R248W					
ERpHER2n	ERpHER2n	ERpHER2n	ERpHER2n	ERpHER2n	HER2p	ERpHER2n	ERpHER2n	ERpHER2n	HER2p	ERpHER2n
Living	Died of Other Causes	Died of Other Causes	Living	Died of Other Causes	Living	Living	Died of Disease	Living	Died of Disease	Died of Other Causes
98.7	30.96666667	49.76666667	111.8666667	41.83333333	2.5	154.7666667	15.16666667	64.36666667	68.06666667	169.8333333
NO	NO	NO	NO	NO	NO	NO	NO	NO	YES	NO

MB_0256	MB_0626	MB_0211	MB_0124	MB_0510	MB_0365	MB_0290	MB_0113	MB_0620	MB_0282	MB_0228	MB_0578	MB_0479
WT	MUT	MUT	WT	WT	MUT	WT	WT	WT	WT	WT	WT	MUT
WT	MISS	MISS	WT	WT	NULL	WT	WT	WT	WT	WT	WT	NULL
	F134L	R248Q			X332_splice							Q136*
ERpHER2n	ERpHER2n	ERnHER2n	ERpHER2n	ERpHER2n	HER2p	ERpHER2n	HER2p	ERpHER2n	ERpHER2n	ERpHER2n	ERpHER2n	HER2p
Living	Died of Disease	Died of Disease	Living	Living	Died of Disease	Living	Living	Living	Living	Died of Disease	Living	Living
200.7	35.53333333	44.8	118.2	128.4	87.23333333	199.5333333	43.16666667	112.8	194.2333333	10.83333333	110.1	132.7666667
NO	NO	NO	YES	NO	NO	NO	YES	YES	YES	NO	YES	YES

MB_0263	MB_0509	MB_0279	MB_0168	MB_0588	MB_0462	MB_0262	MB_0554	MB_0418	MB_0193	MB_0652
WT	MUT	WT	WT	WT	MUT	MUT	WT	WT	WT	MUT
WT	MISS	WT	WT	WT	MISS	NULL	WT	WT	WT	MISS
	R248G				I195T	N131del				R248Q
ERpHER2n	ERpHER2n	ERpHER2n	ERpHER2n	ERnHER2n	HER2p	ERpHER2n	ERpHER2n	ERpHER2n	ERpHER2n	HER2p
Died of Other Causes	Living	Died of Other Causes	Living	Living	Living	Died of Other Causes	Died of Other Causes	Living	Living	Died of Disease
176.0333333	115.3	168.3333333	122.7666667	119.7333333	132.7666667	145.3	111.1666667	102.0666667	19	96.23333333
NO	NO	NO	NO	NO	NO	NO	NO	NO	NO	NO

MB_0638	MB_0188	MB_0617	MB_7225	MB_7141	MB_7234	MB_7089	MB_7030	MB_7004	MB_7119	MB_7263
WT	WT	WT	MUT	WT	WT	MUT	WT	WT	MUT	MUT
WT	WT	WT	MISS	WT	WT	MISS	WT	WT	NULL	MISS
			P278R			P151A			R110Pfs*39	D352Y
ERpHER2n	ERnHER2n	ERnHER2n	ERnHER2n	ERpHER2n	ERpHER2n	ERnHER2n	ERnHER2n	ERpHER2n	ERnHER2n	ERpHER2n
Died of Other Causes	Died of Disease	Died of Other Causes	Died of Disease	Living	Living	Living	Died of Other Causes	Living	Living	Died of Other Causes
103.6333333	31.3	92.83333333	55	138.5666667	222.2	118.9	31.33333333	50.46666667	128.3666667	164.9666667
NO	YES	NO	YES	NO	NO	YES	NO	NO	YES	NO

MB_0174	MB_7182	MB_7039	MB_3797	MB_7112	MB_0308	MB_7230	MB_5452	MB_6195	MB_6317	MB_5460
MUT	WT	MUT	WT	WT	WT	WT	WT	WT	MUT	WT
NULL	WT	MISS	WT	WT	WT	WT	WT	WT	MISS	WT
G154Afs*16		R342P							Y205N	
ERnHER2n	ERpHER2n	ERnHER2n	ERpHER2n	ERpHER2n	ERpHER2n	ERpHER2n	ERpHER2n	ERpHER2n	ERpHER2n	ERpHER2n
Living	Died of Disease	Died of Disease	Living	Died of Disease	Living	Living	Died of Other Causes	Living	Died of Other Causes	Living
78.76666667	49.46666667	49.2	228.3333333	64.7	183.2666667	182.6	16.16666667	202.2333333	29.23333333	218.6333333
YES	YES	NO	NO	YES	NO	NO	NO	NO	NO	NO

MB_5464	MB_5552	MB_5547	MB_6189	MB_6122	MB_6192	MB_4820	MB_5527	MB_5167	MB_5465	MB_5453	MB_5471
WT	WT	MUT	WT	MUT	MUT	WT	WT	WT	MUT	WT	MUT
WT	WT	MISS	WT	NULL	NULL	WT	WT	WT	NULL	WT	MISS
		I255T		R213*	E287Ifs*63				R213*		R248Q
ERpHER2n	ERpHER2n	ERnHER2n	ERpHER2n	ERpHER2n	ERpHER2n	ERpHER2n	HER2p	ERpHER2n	ERnHER2n	ERnHER2n	ERpHER2n
Died of Other Causes	Died of Disease	Living	Living	Living	Died of Other Causes	Died of Disease	Living	Living	Died of Disease	Died of Disease	Living
116.5333333	34.7	98.56666667	240.2	260.2	42.63333333	57.3	180.5666667	208.4	18.8	57.3	185.7666667
NO	NO	YES	NO	YES	NO	NO	YES	NO	NO	YES	NO

MB_5127	MB_4313	MB_4823
WT	WT	MUT
WT	WT	NULL
		p.?
ERpHER2n	ERpHER2n	HER2p
Died of Other Causes	Died of Disease	Died of Other Causes
191.4666667	300.7	282.3
NO	NO	NO
