# Supplementary material for: Transcriptional profiling reveals a subset of human breast tumors that retain wt TP53 but display mutant p53‐associated features
Source: Mol Oncol. 2020 Jun 23;14(8):1640–52. doi: 10.1002/1878-0261.12736 (PMC7400784; doi:10.1002/1878-0261.12736)
Supplement: Supplementary file 5 — Appendix S5. Mutations identified in other sequenced genes. [file MOL2-14-1640-s005.pdf]

Appendix S5

Mutations in other cancer-related genes identified by sequencing in the PM tumors

METABRIC.ID	gene	vaf	reads	TP53.LOH
MB-0054	PIK3CA	0.27	37	NO
MB-0054	ERBB3	0.31	71	NO
MB-0054	NCOR2	0.55	20	NO
MB-0054	MTAP	0.5	58	NO
MB-0066	PTEN	0.225	80	NO
MB-0066	MAP2K4	0.196	56	NO
MB-0066	PIK3CA	0.149	94	NO
MB-0066	CASP8	0.109	46	NO
MB-0131	PIK3CA	0.333	105	YES
MB-0131	KMT2C	0.349	189	YES
MB-0131	NRAS	0.271	107	YES
MB-0131	KMT2C	0.313	281	YES
MB-0131	GATA3	0.373	161	YES
MB-0131	CTCF	0.591	181	YES
MB-0143	KRAS	0.383	162	NO
MB-0143	SIK2	0.328	61	NO
MB-0143	RYR2	0.375	256	NO
MB-0143	ASXL1	0.38	213	NO
MB-0143	CDH1	0.608	166	NO
MB-0143	NCOR2	0.375	40	NO
MB-0143	TBX3	0.383	133	NO
MB-0143	PIK3CA	0.336	211	NO
MB-0143	MUC16	0.167	186	NO
MB-0143	TBX3	0.362	130	NO
MB-0147	CLK3	0.631	65	YES
MB-0147	PIK3R1	0.31	129	YES
MB-0147	EP300	0.25	20	YES
MB-0147	CDH1	0.667	24	YES
MB-0173	FOXO3	0.386	140	YES
MB-0173	DNAH2	0.383	227	YES
MB-0173	UTRN	0.471	257	YES
MB-0173	RYR2	0.408	157	YES
MB-0195	PRPS2	0.353	85	NO
MB-0195	PALLD	0.407	81	NO
MB-0195	TBX3	0.398	161	NO
MB-0195	BAP1	0.709	127	NO
MB-0195	SGCD	0.471	206	NO
MB-0197	SIAH1	0.19	100	NO
MB-0197	SHANK2	0.225	173	NO
MB-0197	STMN2	0.128	430	NO
MB-0197	NCOA3	0.143	28	NO
MB-0197	ROS1	0.169	71	NO
MB-0197	PTPRM	0.151	146	NO
MB-0197	ROS1	0.169	71	NO
MB-0311	MALAT1	0.493	71	NO
MB-0311	COL6A3	0.543	138	NO
MB-0311	AHNAK	0.611	18	NO
MB-0311	MUC16	0.206	155	NO
MB-0311	AFF2	0.122	164	NO
MB-0311	UBR5	0.543	105	NO
MB-0311	MUC16	0.194	170	NO

MB-0311	NCOR1	0.647	17	NO
MB-0311	MALAT1	0.22	100	NO
MB-0311	MAP2K4	0.297	74	NO
MB-0311	JAK1	0.469	81	NO
MB-0311	PIK3CA	0.451	164	NO
MB-0311	RYR2	0.149	208	NO
MB-0311	MALAT1	0.516	126	NO
MB-0311	AFF2	0.214	192	NO
MB-0311	AGMO	0.462	80	NO
MB-0311	SMARCC1	0.263	19	NO
MB-0311	STMN2	0.122	98	NO
MB-0311	DNAH5	0.395	43	NO
MB-0311	ARID2	0.158	38	NO
MB-0393	PIK3CA	0.327	104	YES
MB-0393	SF3B1	0.053	75	YES
MB-0393	PIK3CA	0.254	185	YES
MB-0532	MUC16	0.118	718	YES
MB-0532	PIK3CA	0.254	232	YES
MB-0532	CHD1	0.137	168	YES
MB-0532	DNAH5	0.5	72	YES
MB-0532	CDKN1B	0.21	157	YES
MB-0532	TP53	0.155	155	YES
MB-0532	TBX3	0.108	287	YES
MB-0532	BRCA1	0.488	254	YES
MB-0532	MLL2	0.462	675	YES
MB-0532	AHNAK2	0.409	820	YES
MB-0532	CBFB	0.267	30	YES
MB-0532	PALLD	0.524	82	YES
MB-0532	MLL2	0.447	622	YES
MB-0532	PPP2R2A	0.454	163	YES
MB-0532	TP53	0.143	140	YES
MB-0532	TP53	0.145	145	YES
MB-0532	THSD7A	0.515	270	YES
MB-0542	MEN1	0.433	127	NO
MB-0542	MYO3A	0.439	262	NO
MB-0542	HERC2	0.497	191	NO
MB-0542	TG	0.505	182	NO
MB-0542	SMARCC2	0.425	200	NO
MB-0542	ERBB3	0.486	140	NO
MB-0542	TG	0.471	278	NO
MB-0542	RYR2	0.491	169	NO
MB-0542	PRKG1	0.56	75	NO
MB-0542	NRG3	0.424	184	NO
MB-0542	NPNT	0.409	186	NO
MB-0542	NPNT	0.497	197	NO
MB-0542	CDH1	0.412	51	NO
MB-0542	PIK3CA	0.467	75	NO
MB-0542	DTWD2	0.5	88	NO
MB-0543	PDE4DIP	0.2	40	NO
MB-0666	PIK3CA	0.189	37	NO
MB-2634	SBNO1	0.392	194	NO
MB-2634	ARID1A	0.439	41	NO

MB-2634	PRR16	0.294	201	NO
MB-2634	SYNE1	0.613	31	NO
MB-2634	TP53	0.789	109	NO
MB-2634	LIFR	0.417	151	NO
MB-2634	SBNO1	0.421	57	NO
MB-2634	MLL2	0.566	76	NO
MB-2634	PTPRD	0.281	32	NO
MB-2634	SYNE1	0.539	167	NO
MB-3389	LAMA2	0.175	80	NO
MB-3389	CHD1	0.404	94	NO
MB-3389	ARID2	0.379	124	NO
MB-3389	MYO1A	0.253	75	NO
MB-3389	GATA3	0.579	114	NO
MB-3389	FAM20C	0.851	134	NO
MB-3389	USP28	0.2	35	NO
MB-3466	MLLT4	0.108	185	YES
MB-3466	LAMA2	0.123	195	YES
MB-3466	AKAP9	0.276	199	YES
MB-3466	AFF2	0.18	100	YES
MB-3466	DNAH2	0.363	201	YES
MB-3466	MALAT1	0.242	360	YES
MB-3466	ZFP36L1	0.151	192	YES
MB-3466	ERBB3	0.254	71	YES
MB-3466	NOTCH1	0.146	192	YES
MB-3466	PTPRD	0.329	146	YES
MB-3466	KDM3A	0.312	77	YES
MB-3466	MUC16	0.11	463	YES
MB-3466	SIAH1	0.254	59	YES
MB-3466	NRG3	0.306	235	YES
MB-3466	SMAD2	0.173	208	YES
MB-3466	MALAT1	0.312	157	YES
MB-3466	PTPN22	0.185	92	YES
MB-3466	NPNT	0.181	293	YES
MB-3466	TG	0.277	173	YES
MB-3466	LAMB3	0.255	506	YES
MB-3466	KDM3A	0.135	170	YES
MB-3466	TBL1XR1	0.112	251	YES
MB-3466	HIST1H2BC	0.244	176	YES
MB-3600	MUC16	0.126	309	NO
MB-3600	PTPRD	0.376	165	NO
MB-3600	USP9X	0.444	169	NO
MB-3600	CTNNA1	0.667	21	NO
MB-3600	BIRC6	0.243	37	NO
MB-3600	SETD1A	0.488	82	NO
MB-3600	ATR	0.5	44	NO
MB-3600	FOXP1	0.529	70	NO
MB-3600	GATA3	0.285	242	NO
MB-3600	ROS1	0.365	104	NO
MB-3600	MAP3K1	0.421	164	NO
MB-3600	RUNX1	0.438	208	NO
MB-3600	FANCD2	0.187	182	NO
MB-3600	AHNAK	0.207	300	NO

MB-3600	KMT2C	0.208	72	NO
MB-3600	TG	0.185	634	NO
MB-3600	LAMA2	0.257	183	NO
MB-4148	NCOR2	0.614	153	NO
MB-4148	NCOR2	0.445	110	NO
MB-4148	PTPRD	0.64	150	NO
MB-4148	SYNE1	0.556	81	NO
MB-4148	ALK	0.261	218	NO
MB-4148	HERC2	0.56	134	NO
MB-4148	FOXP1	0.432	162	NO
MB-4148	CHD1	0.482	114	NO
MB-4148	AKT2	0.64	175	NO
MB-4148	SHANK2	0.467	105	NO
MB-4148	EP300	0.475	118	NO
MB-4148	BIRC6	0.667	18	NO
MB-4148	TG	0.331	353	NO
MB-4148	SYNE1	0.533	272	NO
MB-4148	SETDB1	0.318	274	NO
MB-4148	GPR124	0.194	31	NO
MB-4148	AHNAK	0.482	328	NO
MB-4148	NF1	0.443	122	NO
MB-4235	SMAD4	0.458	48	YES
MB-4235	MYH9	0.196	46	YES
MB-4235	SYNE1	0.437	87	YES
MB-4235	PDE4DIP	0.213	253	YES
MB-4235	PIK3CA	0.076	158	YES
MB-4235	MUC16	0.43	179	YES
MB-4235	CBFB	0.154	26	YES
MB-4235	MAP3K1	0.108	166	YES
MB-4278	UBR5	0.115	87	YES
MB-4278	KMT2C	0.126	111	YES
MB-4278	AHNAK2	0.203	153	YES
MB-4278	ARID5B	0.486	148	YES
MB-4278	PIK3R1	0.13	46	YES
MB-4278	UBR5	0.481	27	YES
MB-4278	MAP3K1	0.122	82	YES
MB-4278	KDM3A	0.5	88	YES
MB-4278	KDM6A	0.127	71	YES
MB-4278	LAMB3	0.101	159	YES
MB-4278	GATA3	0.083	121	YES
MB-4278	APC	0.523	128	YES
MB-4278	NCOR1	0.159	82	YES
MB-4278	COL22A1	0.154	78	YES
MB-4278	AGMO	0.104	67	YES
MB-4278	PIK3CA	0.05	181	YES
MB-4278	CACNA2D3	0.448	125	YES
MB-4278	BRCA2	0.125	104	YES
MB-4278	RYR2	0.139	72	YES
MB-4278	MUC16	0.117	154	YES
MB-4278	EP300	0.133	30	YES
MB-4278	MUC16	0.107	214	YES
MB-4278	SYNE1	0.252	135	YES

MB-4278	MALAT1	0.139	72	YES
MB-4278	NF2	0.444	99	YES
MB-4278	DNAH2	0.138	29	YES
MB-4278	NCOR1	0.4	150	YES
MB-4278	MYO1A	0.434	136	YES
MB-4278	FRMD3	0.488	125	YES
MB-4278	HERC2	0.143	112	YES
MB-4767	PBRM1	0.674	46	NO
MB-4767	DNAH2	0.723	47	NO
MB-4767	CHEK2	0.111	90	NO
MB-4767	NCOR1	0.378	90	NO
MB-4767	CACNA2D3	0.292	65	NO
MB-4767	MEN1	0.474	57	NO
MB-4767	LAMA2	0.568	74	NO
MB-4767	COL6A3	0.137	102	NO
MB-4767	SETD2	0.308	117	NO
MB-4767	PDE4DIP	0.318	245	NO
MB-4767	NOTCH1	0.5	40	NO
MB-4767	BIRC6	0.469	98	NO
MB-4834	KMT2C	0.415	82	YES
MB-4834	ARID1B	0.362	152	YES
MB-4834	EP300	0.768	112	YES
MB-4834	L1CAM	0.777	282	YES
MB-4834	LIFR	0.452	93	YES
MB-4834	SHANK2	0.646	457	YES
MB-4834	FANCA	0.446	175	YES
MB-4834	ERBB4	0.505	182	YES
MB-4834	THADA	0.432	370	YES
MB-4834	ROS1	0.3	20	YES
MB-4834	RPGR	0.59	78	YES
MB-4834	COL22A1	0.254	71	YES
MB-4834	SYNE1	0.621	174	YES
MB-4834	NOTCH1	0.494	89	YES
MB-4834	RPGR	0.528	176	YES
MB-4834	STAB2	0.488	291	YES
MB-4834	NCOA3	0.52	331	YES
MB-4834	SBNO1	0.5	54	YES
MB-4834	SMARCC1	0.445	229	YES
MB-4834	MLLT4	0.172	87	YES
MB-4834	SMAD2	0.15	266	YES
MB-4834	MUC16	0.202	382	YES
MB-4834	MAGEA8	0.203	182	YES
MB-4834	GPR124	0.474	135	YES
MB-4834	DNAH5	0.405	311	YES
MB-4834	ASXL1	0.505	412	YES
MB-4834	PIK3R1	0.462	264	YES
MB-4834	UBR5	0.363	91	YES
MB-4834	LAMB3	0.446	352	YES
MB-4834	PDE4DIP	0.191	787	YES
MB-4834	RYR2	0.48	325	YES
MB-4834	MAP3K1	0.475	158	YES
MB-4834	PALLD	0.643	14	YES

MB-4834	RYR2	0.169	267	YES
MB-4834	TAF1	0.218	78	YES
MB-4834	COL6A3	0.536	84	YES
MB-4876	FOXO3	0.423	26	YES
MB-4876	PBRM1	0.529	68	YES
MB-4876	HERC2	0.528	286	YES
MB-4876	SETD2	0.585	135	YES
MB-4876	CACNA2D3	0.511	139	YES
MB-4876	ARID1B	0.524	254	YES
MB-4876	RYR2	0.595	79	YES
MB-4876	DNAH5	0.484	289	YES
MB-4876	KDM6A	0.333	24	YES
MB-4876	EP300	0.487	197	YES
MB-4876	SETD2	0.5	146	YES
MB-4937	DNAH2	0.326	181	NO
MB-4937	TAF4B	0.463	339	NO
MB-4937	USP28	0.735	68	NO
MB-4937	ASXL2	0.268	254	NO
MB-4937	NDFIP1	0.508	120	NO
MB-4937	COL6A3	0.114	272	NO
MB-4937	FOXO3	0.357	56	NO
MB-4937	CHEK2	0.324	173	NO
MB-4937	SMAD2	0.783	46	NO
MB-4937	EP300	0.661	56	NO
MB-4937	AHNAK	0.609	338	NO
MB-4937	CDH1	0.368	182	NO
MB-4937	AHNAK	0.609	340	NO
MB-4937	NRAS	0.133	60	NO
MB-4937	APC	0.572	138	NO
MB-4937	PTPRD	0.409	127	NO
MB-5001	SYNE1	0.244	45	NO
MB-5001	SETD2	0.276	145	NO
MB-5001	GATA3	0.263	137	NO
MB-5001	SGCD	0.327	147	NO
MB-5001	TBL1XR1	0.292	48	NO
MB-5001	HRAS	0.406	202	NO
MB-5001	AFF2	0.492	189	NO
MB-5001	COL6A3	0.328	137	NO
MB-5001	PTEN	0.333	75	NO
MB-5001	LIFR	0.231	238	NO
MB-5001	PPP2R2A	0.384	73	NO
MB-5001	CLK3	0.412	68	NO
MB-5001	ARID5B	0.338	133	NO
MB-5001	CACNA2D3	0.744	43	NO
MB-5001	FAM20C	0.529	104	NO
MB-5001	DCAF4L2	0.19	311	NO
MB-5001	KDM3A	0.321	109	NO
MB-5001	MUC16	0.512	217	NO
MB-5001	ARID1A	0.472	36	NO
MB-5001	AHNAK	0.672	189	NO
MB-5001	PALLD	0.476	103	NO
MB-5001	NRAS	0.447	161	NO

MB-5001	HERC2	0.217	46	NO
MB-5188	TAF4B	0.529	272	NO
MB-5188	FLT3	0.174	23	NO
MB-5188	TAF1	0.259	54	NO
MB-5188	ROS1	0.32	75	NO
MB-5188	SIK2	0.486	173	NO
MB-5188	SYNE1	0.447	141	NO
MB-5188	ARID1A	0.519	241	NO
MB-5188	MALAT1	0.122	197	NO
MB-5188	EP300	0.446	130	NO
MB-5188	ROS1	0.466	73	NO
MB-5188	LAMA2	0.269	119	NO
MB-5188	EP300	0.485	268	NO
MB-5350	AHNAK2	0.574	197	NO
MB-5350	ALK	0.661	112	NO
MB-5350	AHNAK2	0.596	161	NO
MB-5350	AHNAK2	0.545	66	NO
MB-5350	ARID5B	0.555	128	NO
MB-5350	THSD7A	0.366	41	NO
MB-5350	PTEN	0.445	119	NO
MB-5350	MUC16	0.471	174	NO
MB-5350	AHNAK2	0.639	191	NO
MB-5350	STAB2	0.492	122	NO
MB-5350	TBX3	0.153	85	NO
MB-5350	USH2A	0.114	123	NO
MB-5350	CDH1	0.092	119	NO
MB-5467	NA	NA	NA	NO
MB-5521	RYR2	0.391	115	YES
MB-5521	FOXP1	0.121	91	YES
MB-5521	DCAF4L2	0.466	174	YES
MB-5521	CASP8	0.429	35	YES
MB-5521	LDLRAP1	0.587	150	YES
MB-5521	DNAH11	0.106	104	YES
MB-5521	PTPRM	0.444	207	YES
MB-5521	AHNAK	0.428	243	YES
MB-5521	NF1	0.048	145	YES
MB-5521	FAM20C	0.188	16	YES
MB-5521	SYNE1	0.153	85	YES
MB-5521	MEN1	0.124	145	YES
MB-5521	GLDC	0.127	79	YES
MB-5521	PIK3CA	0.06	84	YES
MB-5521	DNAH11	0.112	152	YES
MB-5521	MALAT1	0.493	211	YES
MB-5521	SBNO1	0.425	214	YES
MB-5521	NDFIP1	0.135	52	YES
MB-5521	STK11	0.571	182	YES
MB-5521	NEK1	0.628	43	YES
MB-5521	GATA3	0.083	132	YES
MB-5521	MLL2	0.504	129	YES
MB-5521	PRKG1	0.459	122	YES
MB-5521	ACVRL1	0.594	32	YES
MB-5521	PIK3R1	0.538	130	YES

MB-5521	SYNE1	0.593	54	YES
MB-5552	TP53	0.129	85	NO
MB-5552	ASXL1	0.486	313	NO
MB-5552	TP53	0.165	109	NO
MB-5552	SF3B1	0.123	81	NO
MB-5552	COL12A1	0.335	194	NO
MB-5552	ALK	0.421	216	NO
MB-5552	PDE4DIP	0.106	492	NO
MB-5552	CLRN2	0.41	173	NO
MB-5552	USH2A	0.162	191	NO
MB-5552	HERC2	0.432	139	NO
MB-5552	NT5E	0.367	166	NO
MB-5552	PTEN	0.152	66	NO
MB-5552	EGFR	0.477	128	NO
MB-5552	THSD7A	0.432	111	NO
MB-5552	DNAH11	0.536	330	NO
MB-5552	SBNO1	0.25	20	NO
MB-5575	PTPN22	0.4	90	NO
MB-5575	COL12A1	0.726	124	NO
MB-5575	ASXL1	0.643	252	NO
MB-5575	DNAH2	0.171	82	NO
MB-5575	USP28	0.3	10	NO
MB-5575	PIK3R1	0.329	82	NO
MB-5575	CACNA2D3	0.328	116	NO
MB-5575	COL22A1	0.122	131	NO
MB-5575	COL22A1	0.122	131	NO
MB-5575	NPNT	0.333	96	NO
MB-5575	COL22A1	0.464	181	NO
MB-5575	TP53	0.162	74	NO
MB-5653	HERC2	0.237	38	NO
MB-5653	ARID1A	0.218	307	NO
MB-5653	MAP3K1	0.589	95	NO
MB-5653	SMAD4	0.471	191	NO
MB-5653	AHNAK2	0.506	433	NO
MB-5653	LAMA2	0.197	147	NO
MB-5653	NOTCH1	0.484	153	NO
MB-5653	SBNO1	0.491	224	NO
MB-5653	PALLD	0.543	243	NO
MB-5653	MYH9	0.533	45	NO
MB-5653	ARID1A	0.223	305	NO
MB-5653	PIK3CA	0.189	106	NO
MB-5653	RYR2	0.549	133	NO
MB-5653	NF1	0.456	250	NO
MB-5653	SYNE1	0.435	177	NO
MB-5653	SYNE1	0.218	293	NO
MB-6060	GPS2	0.696	135	NO
MB-6060	BRCA2	0.53	83	NO
MB-6060	ATR	0.122	98	NO
MB-6060	FOXP1	0.543	35	NO
MB-6060	USH2A	0.678	115	NO
MB-6060	DNAH5	0.37	92	NO
MB-6060	BRCA2	0.53	83	NO

MB-6060	NF1	0.138	29	NO
MB-6060	PIK3CA	0.481	189	NO
MB-6060	EP300	0.45	60	NO
MB-6060	FRMD3	0.482	56	NO
MB-6060	AHNAK2	0.177	379	NO
MB-6060	SYNE1	0.629	175	NO
MB-6060	MUC16	0.121	199	NO
MB-6060	COL6A3	0.609	174	NO
MB-6060	MLL2	0.444	90	NO
MB-6060	KMT2C	0.553	38	NO
MB-6060	PDE4DIP	0.301	103	NO
MB-6060	DNAH2	0.122	41	NO
MB-6060	HERC2	0.167	48	NO
MB-6060	USP9X	0.131	61	NO
MB-6060	BRCA1	0.416	101	NO
MB-6060	L1CAM	0.486	109	NO
MB-6060	UBR5	0.744	156	NO
MB-6060	SGCD	0.67	97	NO
MB-6060	PDE4DIP	0.34	241	NO
MB-6060	TP53	0.464	112	NO
MB-6060	SGCD	0.173	52	NO
MB-6060	LIFR	0.494	168	NO
MB-6060	CACNA2D3	0.722	18	NO
MB-6060	USH2A	0.781	32	NO
MB-6060	MAP3K10	0.417	36	NO
MB-6060	SHANK2	0.652	267	NO
MB-6060	THSD7A	0.438	16	NO
MB-6077	KMT2C	0.112	260	YES
MB-6077	AHNAK	0.509	273	YES
MB-6077	L1CAM	0.536	84	YES
MB-6077	FANCA	0.571	191	YES
MB-6077	PIK3CA	0.204	206	YES
MB-6077	PDE4DIP	0.233	511	YES
MB-6077	PTEN	0.148	264	YES
MB-6077	RUNX1	0.444	160	YES
MB-6077	KMT2C	0.387	181	YES
MB-6077	HERC2	0.489	225	YES
MB-6077	KDM3A	0.135	37	YES
MB-6077	PTEN	0.164	165	YES
MB-6077	MYH9	0.342	231	YES
MB-6208	AHNAK2	0.124	129	YES
MB-6208	PDE4DIP	0.149	221	YES
MB-6208	AHNAK	0.493	144	YES
MB-6208	FANCD2	0.125	64	YES
MB-6208	AHNAK2	0.41	205	YES
MB-6208	AHNAK2	0.128	125	YES
MB-6208	USH2A	0.368	19	YES
MB-6208	CDKN2A	0.48	25	YES
MB-6208	AHNAK2	0.15	273	YES
MB-6208	CDKN1B	0.605	43	YES
MB-6208	FANCD2	0.337	190	YES
MB-6208	AHNAK	0.42	69	YES

MB-6208	SYNE1	0.607	168	YES
MB-6208	NCOA3	0.7	110	YES
MB-6208	LAMA2	0.277	94	YES
MB-6208	PIK3CA	0.263	209	YES
MB-6208	AHNAK2	0.124	129	YES
MB-6208	AHNAK2	0.477	222	YES
MB-6208	USH2A	0.652	92	YES
MB-6208	AHNAK2	0.184	141	YES
MB-6208	ASXL2	0.478	138	YES
MB-6284	USP9X	0.28	157	NO
MB-6284	SIK1	0.542	190	NO
MB-6284	FOXP1	0.264	106	NO
MB-6284	BIRC6	0.406	101	NO
MB-6284	USP28	0.691	110	NO
MB-6284	UTRN	0.338	74	NO
MB-6284	PALLD	0.5	58	NO
MB-6284	TAF1	0.4	45	NO
MB-6284	PIK3R1	0.286	119	NO
MB-6284	COL12A1	0.5	80	NO
MB-6284	BIRC6	0.495	99	NO
MB-6284	MLLT4	0.571	70	NO
MB-6284	SMARCC2	0.522	67	NO
MB-6287	NCOR2	0.503	183	PROBABLY
MB-6287	CHD1	0.435	115	PROBABLY
MB-6287	TBX3	0.261	46	PROBABLY
MB-6287	ALK	0.155	110	PROBABLY
MB-6287	NF1	0.407	59	PROBABLY
MB-6287	KDM3A	0.543	92	PROBABLY
MB-6287	AKAP9	0.41	178	PROBABLY
MB-6287	CDH1	0.357	56	PROBABLY
MB-6287	NCOR2	0.358	81	PROBABLY
MB-6287	PIK3CA	0.266	214	PROBABLY
MB-6287	MALAT1	0.413	167	PROBABLY
MB-6287	SEPT7P2	0.333	45	PROBABLY
MB-6287	LAMB3	0.8	60	PROBABLY
MB-6287	NDFIP1	0.491	159	PROBABLY
MB-6287	MYO3A	0.354	127	PROBABLY
MB-6287	RASGEF1B	0.549	71	PROBABLY
MB-6287	ERBB3	0.446	168	PROBABLY
MB-7011	COL6A3	0.405	205	NO
MB-7011	MYH9	0.21	62	NO
MB-7011	RYR2	0.8	65	NO
MB-7011	DNAH11	0.189	106	NO
MB-7011	AHNAK2	0.102	255	NO
MB-7011	DNAH5	0.667	30	NO
MB-7011	LAMA2	0.169	89	NO
MB-7011	AFF2	0.2	225	NO
MB-7011	RB1	0.294	17	NO
MB-7011	LAMA2	0.279	154	NO
MB-7011	ROS1	0.472	72	NO
MB-7011	AFF2	0.199	226	NO
MB-7011	ARID2	0.857	63	NO

MB-7043	COL6A3	0.49	192	NO
MB-7043	SYNE1	0.485	99	NO
MB-7043	ERBB4	0.167	108	NO
MB-7043	GLDC	0.455	101	NO
MB-7043	DNAH11	0.478	134	NO
MB-7043	AKT1	0.19	137	NO
MB-7043	ERBB2	0.629	213	NO
MB-7043	USH2A	0.207	29	NO
MB-7043	USP28	0.368	38	NO
MB-7043	SF3B1	0.063	63	NO
MB-7043	LAMB3	0.517	118	NO
MB-7043	TG	0.578	102	NO
MB-7043	USH2A	0.117	180	NO
MB-7043	BIRC6	0.103	116	NO
MB-7092	FRMD3	0.692	52	NO
MB-7092	ATR	0.521	328	NO
MB-7092	SHANK2	0.12	251	NO
MB-7092	ARID1A	0.129	70	NO
MB-7092	AHNAK	0.393	247	NO
MB-7092	SBNO1	0.227	203	NO
MB-7092	MALAT1	0.105	171	NO
MB-7092	TG	0.477	199	NO
MB-7092	AKT1	0.472	322	NO
MB-7092	ERBB4	0.384	305	NO
MB-7092	DNAH5	0.511	184	NO
MB-7092	TBL1XR1	0.452	155	NO
MB-7092	PRKCZ	0.48	298	NO
MB-7092	UTRN	0.447	94	NO
MB-7092	APC	0.44	25	NO
MB-7092	GATA3	0.22	218	NO
MB-7092	ERBB4	0.51	104	NO
MB-7133	DNAH2	0.686	51	YES
MB-7133	TG	0.517	176	YES
MB-7133	NOTCH1	0.556	153	YES
MB-7133	TG	0.296	115	YES
MB-7133	ARID5B	0.543	70	YES
MB-7133	GLDC	0.747	79	YES
MB-7133	GLDC	0.31	116	YES
MB-7133	PTPN22	0.397	58	YES
MB-7133	GATA3	0.218	262	YES
MB-7133	PTPRM	0.174	46	YES
MB-7133	TAF1	0.19	184	YES
MB-7133	ASXL2	0.629	62	YES
MB-7133	FOXO1	0.375	152	YES
MB-7133	DNAH11	0.571	84	YES
MB-7292	MYO1A	0.541	37	NO
MB-7292	ERBB4	0.553	76	NO
MB-7292	MUC16	0.523	130	NO
MB-7292	PRKACG	0.474	114	NO
MB-7292	STAB2	0.496	135	NO
MB-7292	NCOR2	0.561	66	NO
MB-7292	AKAP9	0.512	121	NO

MB-7292	MALAT1	0.463	149	NO
MB-7292	FANCA	0.4	70	NO
MB-7299	DNAH5	0.357	56	YES
MB-7299	PRKG1	0.429	63	YES
MB-7299	STAB2	0.169	65	YES
MB-7299	MUC16	0.55	149	YES
MB-7299	NOTCH1	0.663	101	YES
MB-7299	CHD1	0.143	28	YES
MB-7299	USH2A	0.621	87	YES
MB-7299	FOXO3	0.41	61	YES
MB-7299	PDE4DIP	0.111	459	YES
MB-7299	EP300	0.205	88	YES
MB-7299	EP300	0.266	154	YES
MB-7299	RYR2	0.658	38	YES
MB-7299	FANCA	0.481	185	YES
MB-7299	PTPRM	0.615	78	YES
MB-7299	BRIP1	0.361	83	YES
